# Exploring children’s exposure to voice assistants and their ontological conceptualizations of life and technology

**DOI:** 10.1007/s00146-022-01555-3

**Published:** 2022-10-19

**Authors:** Janik Festerling, Iram Siraj, Lars-Erik Malmberg

**Affiliations:** Department of Education, University of Oxford, 15 Norham Gardens, Oxford, OX2 6PY UK

**Keywords:** Alexa, Child–technology interaction, Entity, Google assistant, Human–technology interaction, New ontological category hypothesis, Ontology, Voice assistants

## Abstract

Digital Voice Assistants (DVAs) have become a ubiquitous technology in today’s home and childhood environments. Inspired by (Bernstein and Crowley, J Learn Sci 17:225–247, 2008) original study (*n* = 60, age 4–7 years) on how children’s ontological conceptualizations of life and technology were systematically associated with their real-world exposure to robotic entities, the current study explored this association for children in their middle childhood (*n* = 143, age 7–11 years) and with different levels of DVA-exposure. We analyzed correlational survey data from 143 parent–child dyads who were recruited on ‘Amazon Mechanical Turk’ (MTurk). Children’s ontological conceptualization patterns of life and technology were measured by asking them to conceptualize nine prototypical organically living and technological entities (e.g., humans, cats, smartphones, DVAs) with respect to their biology, intelligence, and psychology. Their ontological conceptualization patterns were then associated with their DVA-exposure and additional control variables (e.g., children’s technological affinity, demographic/individual characteristics). Compared to biology and psychology, intelligence was a less differentiating factor for children to differentiate between organically living and technological entities. This differentiation pattern became more pronounced with technological affinity. There was some evidence that children with higher DVA-exposure differentiated more rigorously between organically living and technological entities on the basis of psychology. To the best of our knowledge, this is the first study exploring children’s real-world exposure to DVAs and how it is associated with their conceptual understandings of life and technology. Findings suggest although psychological conceptualizations of technology may become more pronounced with DVA-exposure, it is far from clear such tendencies blur ontological boundaries between life and technology from children’s perspective.

**Supplementary Information:**

The online version contains supplementary material available at 10.1007/s00146-022-01555-3.

## Introduction

Within home and childhood environments, a global socio-technical change has occurred over the last decade through the growing presence of commercially available Digital Voice Assistants (DVAs) like Amazon’s ‘Alexa’, Apple’s ‘Siri’, or Google’s ‘Google Assistant’ (Vlahos 2019). For educational and developmental research, DVAs are not only relevant in terms of their socio-technical omnipresence across the globe, but also in terms of their ontological nature as experienced by today’s children (e.g., due to DVAs’ conceptual parallels with organically living entities, such as their capacity to emulate peculiar qualities of human beings like the autonomous use of human language and speech; Festerling and Siraj 2020, 2021; Harwood and Eaves 2020; Nass and Brave 2005).

Therefore, and similar to robotic technologies (e.g., Gaudiello et al. 2015), it can be hypothesized that being exposed to DVAs significantly reshapes both the way today’s children construct their understandings of technology in and of itself, and the way they conceive technology in relation to other things in their environment. This study aims to contribute to the empirical investigation of this basic hypothesis.

The original hypothesis that the growing presence of digital technologies could change how humans have traditionally conceptualized the environment according to basic ontological qualities dates back to Turkle's (1984/2005, 2017) and Papert's (1980) seminal work on the socio-technical role of technology in human development. In short, this work suggested technology impacts thought. More recently, Kahn et al. (2006, 2007, 2009, 2011, 2012) formalized this line of reasoning with the ‘new ontological category hypothesis’ (NOCH), stating increasingly sophisticated technologies may cut across traditional conceptualizations of mutually exclusive ontological categories (e.g., organically living vs. technological entities). In other words, children growing up with such technologies may “see, conceptualize, and interact with them as a unified entity, and not merely a combinatorial set of its constituent properties” (Kahn et al. 2013, p.35). From an empirical perspective, the challenge about NOCH remains its wide argumentative scope how there is a general socio-technical change at the macro-level of society which, in turn, is associated with developmental changes in affected cohorts. However, if NOCH was supported, the socio-technical change at the macro-level of society would become manifest not only *across* different cohorts but also within a single cohort and *across* different levels of individual exposure to the socio-technical change of interest. In other words, if the growing socio-technical presence of DVAs was systematically related to the way today’s children conceptualize their environments according to basic ontological qualities, individual differences in children’s environments (i.e., how much an individual child is exposed to DVAs) should ceteris paribus be related to individual differences in children’s ontological conceptualizations of their environments.

Recent research suggests children growing up with DVAs can indeed have very nuanced understandings of technology’s ontological uniqueness (e.g., accuracy, speed, programmability, assistive purpose, lack of common sense) vis-à-vis humans’ ontological uniqueness (e.g., common sense, moral value, non-programmability; Festerling and Siraj 2020; Xu and Warschauer 2020). But, in the context of this study, the additional question would be whether such nuances are systematically associated with children’s exposure to technology. A unique and, up to this point, unreplicated attempt to investigate this from a cross-sectional perspective was Bernstein and Crowley’s (2008) study (*n* = 60, age 4–7 years) on children’s ontological conceptualizations of prototypical organically living and technological entities (e.g., humans, cats, plants, computers, robots/rovers), and how these conceptualization patterns differed with children’s individual ‘real-world’ exposure to robotic technologies (i.e., exposure in terms of children’s robot-related interest, knowledge, experience etc.).^1^ One of their main empirical findings was children with higher exposure to robotic technologies seemed to have different conceptual understandings of robots/rovers compared to children with lower exposure (e.g., children with higher exposure to robotic technologies discriminated between organically living entities and robots/rovers more on the basis of psychology and less on the basis of intelligence).

This lead to the hypothesis that in future cohorts “children raised increasingly amid intelligent technologies will grow up thinking differently about some [ontological] concepts that developmental psychologists have previously considered universal and inevitable” (Bernstein and Crowley 2008, p.242). Most importantly (and as the following discussion will show in more detail), the ontological concepts referred to in this context draw on the most basic constituents of life, namely biology, intelligence and psychology.

As Bernstein and Crowley (2008) mentioned in their original study, at least 2 million early generation robotic vacuum cleaners so-called ‘Roomba’ were sold to households in 2008 (including homes of two children who participated in their study). Therefore, in light of hundreds of millions of DVAs populating childhood environments across the globe in the early 2020s, the overdue question is to what extent these state-of-the-art manifestations of intelligently behaving technologies could have nurtured what Bernstein and Crowley (2008) originally found. This was our motivational starting point for the current study, and we addressed this question for a sample of children born a decade later than those in Bernstein and Crowley’s (2008) study—and who grew up in even further technologized home and childhood environments, as exemplified by DVAs’ socio-technical omnipresence across the globe.^2^

In the following, we begin with a review of research on children’s ontological understandings of life and technology and recent research on DVAs’ presence in today’s home and childhood environments.

## Alexa, are you alive? Children’s ontological conceptualizations of life and technology

As Westall and Brack (2018) note, “there [are] as many definitions of life as there are people trying to define it” (p.3). Yet, even without scientists agreeing on what exactly life is, people have an intuitive understanding of what it means (Zimmer 2021), and for any given entity, one would expect this intuitive judgement (living vs. non-living) to be consistent with other ontological qualities used to conceptualize the same entity (Gelman 1988). However, an array of research has shown how children systematically tend to use certain life-like qualities to conceptualize technological entities while refraining from using other life-like qualities (e.g., Beran et al. 2011; Bernstein and Crowley 2008; Hughes et al. 1987; Jipson and Gelman 2007; Kahn et al. 2006, 2012; Melson et al. 2005; Okita and Schwartz 2006; Saylor et al. 2010; Scaife and Van Duuren 1995). This is in line with recent experimental research showing how children systematically place a robot somewhere between humans and computers, even after children are taught the ontological ‘truth’ about the robot’s lack of human psychological capacities as part of the experimental condition (van Straten et al. 2020), or Xu and Warschauer’s (2020) exploratory research on children’s tendency to attribute both animate and inanimate properties to DVAs.

One interpretation of such findings is that children’s seemingly ‘contradictory’ understandings of technology should eventually converge to an *a *priori definable and metaphysically ‘true’ end-state as they grow older and learn the ‘truth’ about technology—an end-state, in which, for example, a programmable technological entity cannot share substantial psychological similarities with organically living entities, such as humans (e.g., van Straten et al. 2020). An alternative interpretation of such findings—in line with NOCH—is that children’s ‘contradictory’ understandings of technology reflect unique and developmentally stable patterns, therefore prefiguring future stances towards technology (e.g., Severson and Carlson 2010; Turkle, 1984/2005, Turkle 2005). In other words, there may be no a priori definable and metaphysically ‘true’ end-state for how one should conceptualize technology vis-à-vis organically living entities, and no developmentally inferior or superior way of doing so (Festerling and Siraj 2021).

But exposure to technology could prompt children to develop more nuanced understandings of prototypical entities in their environments, such as the ontological differences between technological entities, on the one hand, and organically living entities, on the other hand. Such nuances can already be found in in the empirical literature on children’s engagements with technology. For example, some children in Turkle’s (1984/2005, 2017) ethnographic studies provided well-argued reasons why the programmable nature of technological entities would make them more reliable, consistent and trustworthy than humans.

When it comes to DVAs, exploratory qualitative findings by Festerling and Siraj (2020) on children’s open engagements with DVAs suggest children seem to appreciate the instant social gratification and excitement they experience with DVAs (see also Oranç and Ruggeri 2021), and they also associate DVAs with relative ontological strengths. For example, children systematically conceptualized DVAs to have higher accuracy levels and faster response times for knowledge-related domains of intelligence (e.g., provision of facts) and explained their conceptualization patterns by DVAs’ connectedness to the internet and their programmable nature (Festerling and Siraj 2020). This is in line with other empirical findings in the literature on children’s differentiated perceptions of computers as data-based knowledge sources (e.g., Rücker and Pinkwart 2016; Wang et al. 2019), or how first-hand experience in building and programming robots can yield more nuanced ontological conceptualization patterns (e.g., Gaudiello et al. 2015). Furthermore, Oranç and Küntay (2020) found even when children think a robot is intelligent enough to answer questions related to mechanical or electronic topics, for biological and psychological questions (e.g., ‘Why do humans sleep?’, ‘Why do people help each other?’), children still prefer humans as knowledge sources. Similarly, Festerling and Siraj (2020) found children associate other domains of intelligence with humans (e.g., conversational comprehension, common sense, creativity), which is further in line with Xu et al. (2021) experimental findings on how children seem to elevate the intelligibility of their speech according to DVAs’ perceived conversational weaknesses. Lastly, Yip et al. (2019) found although children expect DVAs to make them laugh in response to certain commands (e.g., commands to make farting noises), DVAs which would have the psychological ability to laugh themselves were thought of as being utterly disturbing.

Taken together, these empirically observable nuances in children’s conceptualizations of technological entities suggest children can have very nuanced understandings and expectations regarding the ontological nature of technology. But what kind of ontological qualities should be considered when studying children’s conceptualization patterns across a diverse range of prototypical entities? According to Sternberg et al. (1981), humans’ implicit understandings of ‘intelligence’ are related to cognitive domains (e.g., task-oriented problem solving, possession of knowledge, using language and speech) as well as non-cognitive domains (e.g., being sensitive with others, behaving politely, taking responsibility). This is in line with Herrmann et al. (2007) evolutionary account of intelligence, suggesting that instead of assuming there is a model of ‘general intelligence’ in nature, species differ in their combined mastery of cognition (e.g., spatial memory, discrimination of quantities, understanding causality) and social cognition (e.g., recognition of intentions, possession of attentional states). Therefore, and in line with Bernstein and Crowley (2008), this study refers to ontological qualities generally related to cognition as ‘intelligence’, and ontological qualities generally related to social cognition as ‘psychology’. These two ontological dimensions are complemented with a third ontological dimension referred to as ‘biology’, which stands for ontological qualities related to entities’ organic aliveness (e.g., metabolism, mortality, growth).

In sum, for operational purposes in the context of this study, the above-mentioned ontological dimensions (biology, intelligence, psychology) define the concept of ‘life’ as an open ‘ontological space’ in which entities can be placed anywhere alongside other entities depending on how different ontological qualities within these dimensions are used to characterize them. Importantly, this is not meant to be a conclusive definition of the concept of ‘life’—only a practical way of capturing different understandings and applications of it in the context of this study.

## Alexa, how have you changed our lives*?* An account of children’s exposure to DVAs in today’s home and childhood environments

The earliest sphere of human development, the family home, sets the first primary stage for children to develop conceptual understandings of their increasingly technologized environments (Papert 1980), before their horizons begin to widen during middle childhood when they enter other social environments such as pre-schools, primary schools, neighborhood environments or other family homes (Bronfenbrenner and Morris 2006; Huston and Ripke 2006). Over the last decade, commercial DVAs have become a ubiquitous technology in these broader home and childhood environments. As voice-enabled dual-purpose devices, DVAs motivate user engagements through the (1) usefulness of utilitarian functionalities (e.g. access to streaming services, control of smart home applications, functionalities related to communication and productivity, online shopping, information search), and (2) the enjoyment of hedonic functionalities (e.g. interactive games, basic conversational capabilities, pre-programmed personalities Moussawi et al. 2020; Wu and Lu 2013). In the growing body of research investigating how families and their children engage with DVAs in everyday life, utilitarian functionalities often dominate empirical findings, such as streaming media content (e.g. music, audiobooks, podcasts, news), information search (e.g. general knowledge seeking, weather forecasts, cooking recipes), seeking daily practical assistance (e.g. setting alarms, setting ambient sounds before going to bed, setting routines), or controlling smart home devices (e.g. lights, thermostats; Ammari et al. 2019; Festerling 2019; Garg and Sengupta 2020; Lopatovska and Williams 2018; Lovato et al. 2019; Porcheron et al. 2018; Sciuto et al. 2018). Despite drops in average usage intensities often occurring after an initial period of excitement and experimentation, longitudinal insights also show how some families establish very stable usage habits with little variation in intensity over time (Garg and Sengupta 2020; Sciuto et al. 2018). This is not surprising, given DVAs are often placed in the middle of daily family life, both spatially and socially. For example, popular locations for DVAs within households include living rooms, kitchens or dining rooms (Ammari et al. 2019). Therefore, it is also not surprising that semi-naturalistic observations of daily family life have revealed how DVAs become “embedded in the life of the home” (Porcheron et al. 2018, p.9, emphasis in original), for example by serving as a means for new family rituals and bonding activities, or as a source of competition and rivalry in more stressed situations (Beirl et al. 2019). Hence, and in contrast to other modern technologies which are often blamed to isolate users (e.g., smartphones), DVAs’ communally accessible voice interfaces have been found to enhance social harmony in families, similar to the effect of a new pet (Lee et al. 2020). In addition, voice-only communication has previously been found to enhance psychological connections (e.g., empathetic accuracy) between human engagement partners (Kraus 2017), which may similarly apply to voice-only communication with technological entities (e.g., DVAs).

In summary, DVAs are a means to an end as well as an end in themselves, because they either serve as interfaces which allow families and their children to access functionalities which would also be accessible on other devices, or they serve as independent social engagement partners in their own right, usually placed in the middle of daily family life, both spatially and socially. Compared to many other technologies which characterize today’s home and childhood environments, this is what makes DVAs in interesting empirical case for an extension of Bernstein and Crowley’s (2008) original study. Importantly, especially due to the different age ranges under investigation in both studies and age-related developmental differences, we understand our study as an extension rather than a replication of Bernstein and Crowley (2008). Therefore, we also did not expect our results to be identical to the original study. This issue is discussed in more detail in the context of limitations and directions for future research.

## Research questions

From the above discussion, the following two main research questions were posed:

1. What general patterns emerge for children’s ontological conceptualizations of contemporary prototypical entities?
2. How are these ontological conceptualization patterns associated with children’s individual exposure to DVAs in their home and childhood environments?

In our attempt to address these main research questions empirically, we also explored the role of additional control variables in the context of DVA-exposure and ontological conceptualization patterns, such as children’s technological affinity and basic demographic characteristics (e.g., age, gender, parental education). These supplementary investigations are discussed alongside the main focus of our study.

## Methodology and research design

The study applied a correlational research design based on a cross-sectional sampling procedure and naturally occurring variation in children’s environmental DVA-exposure and ontological conceptualization patterns.

This study focused on children in the midst of their *middle* childhood (7–11 years). The reason was twofold. First, at the upper limit of the age range, children in this study were supposed to be young enough—from today’s perspective (i.e., as of the year 2020/2021)—to be part of humankind’s first ‘DVA-cohort’ (referring to the first cohort who grew up in a world populated with DVAs). Given the first commercial DVAs were released between 2011 and 2013 (Mutchler 2017), this corresponds to an upper limit of 11 years, because 11-year-old children in 2020/2021 were born right before DVAs’ initial commercial breakthrough. Second, cohort effects are interesting insofar as they reflect potentially persistent and developmentally stable patterns of human development within a particular cohort. Or, in the words of Turkle (1984/2005), “instead of thinking in terms of adult ideas ‘filtering down’ to children, it makes more sense to think of children’s resolutions [of technology] prefiguring new positions for the computer culture to come” (p.59). Therefore, the lower limit of the age range was 7 years, which developmentally corresponds to the mature beginning of middle childhood and children’s emerging bridges to adolescence and adulthood (Huston and Ripke 2006).

Upon ethical approval by the institutional review board and small-scale piloting of the data collection methods, parent–child dyads from the United States (US) were recruited using the crowdsourcing platform ‘Amazon Mechanical Turk’ (MTurk). Parent–child dyads completed a combined parent–child survey (i.e., survey part A completed by the parent and survey part B completed by the child).^3^ Although MTurk has become a popular data collection platform in social science research due to its effectiveness (e.g., data quality in terms of reliability and validity) and efficiency (e.g., resource expenditures per respondent; Buhrmester et al. 2011), use cases in developmental and educational research are still rare. A few studies have used MTurk to collect survey data from parents (e.g., Schneider et al. 2015; Sweeny et al. 2015), including surveys with families using DVAs (e.g., Ammari et al. 2019; Richards and Dignum 2019). But, to the best of our knowledge, there is only one study which has used MTurk to collect data from parent–child dyads (Tran et al. 2017). Facing various COVID-19 related restrictions and uncertainties for traditional means of data collection in developmental and educational research (e.g., school-based recruitment) at the time of the data collection, the decision was made to further probe the feasibility of MTurk for parent–child surveys as part of this study. As a consequence of this decision, our study became limited in the sense that MTurk samples in the US cannot be assumed to be representative of the general US population (Difallah et al. 2018), although it has also been suggested that most demographic deviations become negligible once basic demographic control variables (e.g., age, gender, education, ethnicity) are taken into account (e.g., Levay et al. 2016).

To address recent criticisms regarding low remuneration levels on MTurk (e.g., Hara et al. 2018; Samuel 2018), the remuneration for this study corresponded to an hourly wage of $10/h. To also disincentivize the inclusion of children for financial reasons, the base of the remuneration was limited to the pre-calculated average time parents were expected to spend on their part of the survey (∼14 min), yielding a total remuneration of $2.3 per valid response. To identify valid responses, five authenticity screening measures were applied: (1) minimum overall survey response time of 12 min (20th percentile cut-off value), (2) minimum average time of 8.5 s spent on each item of the ontological conceptualization task (20th percentile cut-off value), (3) response consistency of children’s age across part A and B of the survey, (4) response consistency of children’s gender across part A and B of the survey, and (5) a correctly solved attention measure (correctly solving a simple additive equation). To ensure high data quality, responses were only included in the subsequent analysis if none of these measures were violated. Arguably, the remaining uncertainty about the data quality (e.g., someone authentically pretending to be both a parent and a child by submitting a response which passes all five authenticity screening measures) is inherent to the nature and, therefore, the limitations of anonymous online data collection methods, in general.

In total, 280 responses were collected, which corresponds to the maximum sample size given the fixed resource constraints of this study. After the authenticity screening, 137 responses were excluded. Although this may seem like a high exclusion rate, it is in line with the exclusion rates found in previous studies using MTurk to collect data from parent–child dyads (see Tran et al. 2017). Of the remaining 143 parent–child dyads included in the subsequent analysis (*M* = 8.36 years, *SD* = 1.22), 40 children were 7 years old, 51 were 8 years old, 23 were 9 years old, 19 were 10 years old and 10 were 11 years old. In total, 55% of the children were boys (*n* = 78) and 46% girls (*n* = 65), while 54% of parental respondents were mothers (*n* = 77) and 46% were fathers (*n* = 66). Most parental respondents reported to have an undergraduate (*n* = 70) or postgraduate (*n* = 47) college degree. Furthermore, 90% of parents (*n* = 129) reported that English was the only language spoken at home, while 10% of parents (*n* = 14) reported there was at least one additional language spoken at home, including Spanish (*n* = 9), French (*n* = 6) and Mandarin (*n* = 4) (see online supplementary materials for further details).

### Data collection methods and measures

There were two empirical constructs of main interest in this study: children’s ontological conceptualizations of prototypical entities (dependent variable) and children’s DVA exposure (independent variable). Both are discussed in the following.

#### DVA-exposure

Similar to Bernstein and Crowley (2008), children’s DVA-exposure was measured using a point-based system counting different technology-related experiences, including primary engagements with DVAs (1 point + 1 point if DVAs were used regularly by the child, e.g., several times per week), general presence of DVAs at home (1 point + 1 point if DVAs were used on more than 1 device + 1 point if a DVA was installed in child’s room), presence of DVAs within the child’s closer circle of family and friends (1 point), and familiarity with DVAs through media (1 point). Importantly, due to the diverse hardware across commercial DVA-ecosystems (e.g., smart speakers, smart TVs, smartphones, smart home appliances, wearables, car entertainment systems), equating DVAs with smart speakers would mean to misconceive the unique nature of how DVAs enter into today’s home and childhood environments (Festerling and Siraj 2021). For this reason, our empirical investigation did not narrow ‘DVA-exposure’ to one kind of hardware (e.g., smart speakers).

The complete set of these DVA-exposure items was administered to parents (survey part A) and a shortened set of DVA-exposure items was administered to children (survey part B) as a control. With this point-based system, exposure scores were computed for each child ranging from 0 to 7 (see Appendix Fig. 3). Since DVA-exposure scores based on parental responses were strongly correlated with DVA-exposure scores based on children’s responses, *r*(138) = 0.73, *p* < 0.01, we only used parental DVA-exposure scores in the analysis.

#### Ontological conceptualization patterns

**Fig. 1:**
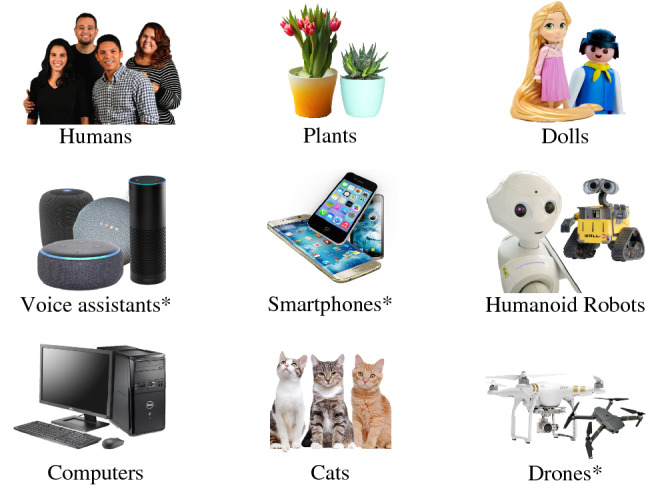
Entities for extended forced-choice conceptualization task. Notes. Figure shows pictures of entities used in the conceptualization task. The task uses nine different entities: three organically living entities (humans, cats, plants), five technological entities (humanoid robots, drones, DVAs, desktop computer, smartphones) and one other entity (dolls). Marked items (*) were either added (voice assistants) or adjusted (calculators → smartphones; rovers → drones) compared to Bernstein and Crowley’s (2008) original study

Children’s ontological conceptualization patterns were measured based on an adjusted version of Bernstein and Crowley’s (2008) original conceptualization task. For entities (see Fig. 1), the following adjustments were made: (1) all entities were visually presented in a plural rather than singular form (e.g., picture of several humans vs. picture of a single human) to probe more abstract conceptualizations of the entities presented; (2) two ‘old-fashioned’ technological entities (calculator and rover) from the original study were replaced by more modern entities (smartphones and drones); and (3) DVAs were included as a ninth entity.

Each ontological dimension (biology, intelligence, psychology) was measured using five forced-choice items (0 = ontological quality *not* assigned to entity; 1 = ontological quality assigned to entity).^4^ Compared to Bernstein and Crowley’s (2008) original conceptualization task, the following adjustments were made: (1) for biology, the original item on *reproduction* was replaced by an item on mortality; (2) for psychology, three items on empathy, moral value and moral consciousness were added to ensure equal item coverage for all three ontological dimensions; (3) for intelligence, the original item on situational awareness—already found to be problematic by Bernstein and Crowley (2008)—was replaced by a general item on ‘intelligence’, and the item on remembering was concretized by adding speech / language comprehension to also consider this important aspect of intelligence as part of the ontological dimension (see Table 1).

**Table 1:** Items for forced-choice conceptualization task

Attribute dimension	Ontological attribute	No	Item
(I) Biology (animate)	Aliveness	1	Tick the ones that are alive
	Growth	2	Tick the ones that can grow (what we mean is, if we looked at these things a long time from now, they would be bigger)
	Metabolism	3	Tick the ones that need food or water
	Movement	4	Tick the ones that can move by themselves
	Mortality	5	Tick the ones that die when they are old*
(II) Intelligence	Calculation	6	Tick the ones that can calculate something*
	Learning	7	Tick the ones that can learn how to do new things
	Planning	8	Tick the ones that could figure out themselves how to do something if you told them what to do
	Remembering	9	Tick the ones that can remember things (what we mean is, if you told them something today, they could remember it tomorrow)*
	Intelligence/Smartness	10	Tick the ones that can be intelligent or smart*
(III) Psychology	Emotionality	11	Tick the ones that can feel happy or sad
	Empathy	12	Tick the ones that could sense how you feel*
	Moral value	13	Tick the ones that could feel pain if you hit them*
	Moral consciousness	14	Tick the ones that would feel bad if they knew they did something wrong*
	Volition	15	Tick the ones that could make a decision if you gave them a choice

Table 1 shows the extension of Bernstein and Crowley’s (2008) original forced-choice conceptualization task according to three different attribute dimensions (i.e. [I] Biology, [II] Intelligence, [III] Psychology). In total, 15 different forced-choice items are used, each one referring to a separate ontological attribute within its dimension. Marked items (*) were either added (items 5, 11, 13, 14, 15) or adjusted in their wording (item 7: ‘[…] that can add numbers together’ → ‘[…] that can calculate something’). For the sake of completeness, a 16^th^ item on artificiality (‘Tick the ones that were made in factory’) from Bernstein and Crowley’s (2008) original conceptualization task was included in the survey but not used in the exploratory course of our data analysis. Therefore, it is not included in the table

For each item (e.g., ‘Tick the ones that are alive’), all entities were presented in a 3 × 3 matrix with corresponding tick boxes below each entity. The order of forced-choice items was randomized for each child, while the order of entities within each matrix remained constant throughout each questionnaire to facilitate response selection for children. Although a constant order of entities throughout an individual questionnaire could prompt children to routinize their response behaviors (thereby potentially becoming inattentive ‘fast-clickers’ selecting the same entities for all ontological conceptualization items), we considered this risk negligible due to the minimum average time we specified for ontological conceptualization items as part of the authenticity screening process (see above).

#### Technological affinity and other control variables

In addition to children’s DVA-exposure, a child-adjusted version of Karrer et al. (2009) ‘technological affinity questionnaire’ (TAQ) was used to measure children’s self-reported technological affinity, which is defined as a multi-dimensional personality trait expressing one’s general attitude towards and familiarity with technology.

Since the introduction of personal computers in the 1970s and 1980s, personality-related constructs on people’s attitudes towards technology (e.g., ‘computer attitudes’, ‘computer anxiety’, ‘computer aversion’, ‘computer self-efficacy’, ‘technology readiness’, see Attig et al. 2017 for an overview) have been found to be associated with various other characteristics in adults (e.g., Anthony et al. 2000; dos Santos and Santana 2018; Horstmann et al. 2018; Korukonda 2005, 2007; Nitsch and Glassen 2015; Powell 2013; Saleem et al. 2011) as well as children (e.g., Baloğlu and Çevik 2008; Chou 2001; Cooper 2006; King et al. 2002; Rees and Noyes 2007; Todman and Lawrenson 1992; Todman and Monaghan 1994). Therefore, the main reason for including the TAQ was to control for parallel associations between children’s technological affinity and their DVA-exposure (e.g., higher technological affinity associated with higher DVA-exposure), or between children’s technological affinity and their ontological perceptions of technology (e.g., more positive attitudes towards technology associated with more socially salient perceptions of technology; see Beran et al. 2011; Etzrodt and Engesser 2021). In line with Karrer et al. (2009) original TAQ, the child-adjusted version differentiates four dimensions of technological affinity (each measured with a five-item sub-scale): positive attitude towards technology, negative attitude towards technology, excitement about technology and technological competency. Prior to the main analysis, we examined the structural validity of the child-adjusted TAQ using the exploratory structural equation modeling (ESEM) framework by Asparouhov and Muthén (2009). In total, seven items were meaningfully re-assigned before the main analysis, and, after the re-assignment, all revised sub-scales had acceptable reliability estimates of internal consistency (positive attitude *α* = 0.78; negative attitude *α* = 0.87; excitement *α* = 0.81; competency *α* = 0.73; total TAQ *α* = 0.78) as indicated by the ordinal coefficient alpha (Zumbo et al. 2007; Zumbo and Kroc 2019; see online supplementary materials for further details on structural validity and re-assignment of items).

Together with other survey items (e.g., children’s and parents’ demographic characteristics), the above-discussed variables formed the basis of the parent–child survey. The survey was programmed using the online platform Qualtrics. Prior to the study, the survey was iteratively piloted and revised with a small number of individuals. Main revisions included the improvement of item wordings in the age-adjusted TAQ and the inclusion of text-to-speech audio files for longer descriptive text paragraphs in the survey.

## Results

The main analysis was conducted using *IBM SPSS Statistics 27.0*. Prior to the main analysis, the child-adjusted TAQ was examined using *Mplus*, version 8.5 (Muthén and Muthén 2017). Reliability estimates of internal consistency, as indicated by the ordinal coefficient alpha (Zumbo et al. 2007; Zumbo and Kroc 2019), were calculated using R (R Core Team 2022) and the ‘semTools’ package (Jorgensen et al. 2022). This section summarizes empirical results (see online supplementary materials for SPSS syntax and Mplus syntax).

### Children’s general ontological conceptualization patterns

Children’s responses to forced-choice items were used to compute biology, intelligence and psychology scores for each entity by adding up how many times a child selected an entity across all five biology, intelligence and psychology items (e.g., if a child selected ‘humans’ on 4 out of 5 biology items, the ‘human biology score’ for this child was equal to 4). Therefore, all scores ranging from 0 to 5 and were treated as continuous variables in the subsequent analysis (see Table 2 for full overview).

**Table 2:** Children’s ontological conceptualization patterns per entity

Entity	(I) Biology		(II) Intelligence		(III) Psychology		Correlations and ANOVA
							(I) × (II) = 0.68**
							(I) × (III) = 0.70**
							(II) × (III) = 0.60**
Humans	*M* = 3.73 (1.58)	α = 0.92	*M* = 2.91 (1.82)	*α* = 0.91	*M* = 3.83 (1.44)	*α* = 0.87	*F*(1.86, 264.21) = 39.51, *p* < 0.01
							(I) × (II) = 0.43**
							(I) × (III) = 0.61**
							(II) × (III) = 0.63**
Cats	*M* = 3.32 (1.84)	*α* = 0.94	*M* = 1.07 (1.35)	*α* = 0.85	*M* = 2.48 (1.59)	*α* = 0.84	*F*(1.78, 253.04) = 155.49, *p* < 0.01
							(I) × (II) = 0.12*
							(I) × (III) = 0.23**
							(II) × (III): *r*(141) = 0.47**
Plants	*M* = 2.55(1.23)	*α* = 0.63	*M* = 0.31 (0.84)	*α* = 0.93	*M* = 0.52 (0.85)	*α* = 0.68	*F*(1.60, 227.49) = 293.17, *p* < 0.01
							(I) × (II) = 0.18*
							(I) × (III) = 0.44**
							(II) × (III) = 0.37**
DVAs	*M* = 0.28 (0.65)	*α* = 0.79	*M* = 1.87 (1.62)	*α* = 0.82	*M* = 0.78 (1.06)	*α* = 0.80	*F*(1.55, 220.00) = 94.17, *p* < 0.01
							(I) × (II) = 0.22**
							(I) × (III) = 0.39**
							(II) × (III) = 0.28**
Smartphones	*M* = 0.32 (0.66)	α = 0.73	*M* = 2.48 (1.47)	α = 0.72	*M* = 0.62 (0.96)	α = 0.79	*F*(1.58, 224.01) = 219.01, *p* < 0.01
							(I) × (II) = 0.25**
							(I) × (III) = 0.44**
							(II) × (III) = 0.47**
Robots	*M* = 0.52 (0.80)	*α* = 0.80	*M* = 2.01 (1.50)	*α* = 0.75	*M* = 0.85 (1.15)	*α* = 0.82	*F*(1.76, 249.61) = 97.60, *p* < 0.01
							(I) × (II) = 0.02
							(I) × (III) = 0.32**
							(II) × (III) = 0.30**
Computers	*M* = 0.20 (0.51)	*α* = 0.73	*M* = 2.11 (1.45)	*α* = 0.74	*M* = 0.46 (0.79)	*α* = 0.75	*F*(1.42, 203.10) = 183.59, *p* < 0.01
							(I) × (II) = 0.11
							(I) × (III) = 0.47**
							(II) × (III) = 0.17*
Drones	*M* = 0.40 (0.65)	*α* = 0.69	*M* = 0.64 (0.97)	*α* = 0.77	*M* = 0.28 (0.69)	*α* = 0.83	*F*(1.58, 225.38) = 9.81, *p* < 0.01
							(I) × (II) = 0.37**
							(I) × (III) = 0.29**
							(II) × (III) = 0.43**
Dolls	*M* = 0.25 (0.67)	*α* = 0.87	*M* = 0.22 (0.54)	*α* = 0.75	*M* = 0.45 (0.81)	*α* = 0.74	*F*(1.82, 257.90) = 6.99, *p* < 0.01

For each of the 9 entities and respective ontological scores (*S*), Table 2 shows the frequencies, mean values (*M*), standard deviations (in parentheses) and reliability estimates of internal consistency (α) as indicated by the ordinal coefficient alpha (Zumbo et al. 2007; Zumbo and Kroc 2019). All scores range from *S* = 0 to *S* = 5. Right column shows Pearson correlations between (I) biology scores and (II) intelligence scores (first row), (I) biology scores and (III) psychology scores (second row), and (II) intelligence scores and (III) psychology scores (third row). *Indicates significant correlation coefficients at the 0.05 level (2-tailed). **Indicates significant correlation coefficients at the 0.01 level (2-tailed). ANOVA results refer to the within-subjects main effect of a repeated measure model with the three ontological scores as a within-subjects factor. For all entities, data failed to meet the sphericity assumption as indicated by Maulchy’s test of sphericity, so the Greenhouse–Geisser correction was applied for all models. Full sample (*n* = 143) was used for all statistics. No missing values in the sample

Correlation patterns of ontological scores within entities suggest children’s conceptualizations of an entity’s biology and psychology were more closely related compared to biology and intelligence, because correlations of the former score pair were consistently stronger across all entities compared to the latter score pair (see right column in Table 2). Furthermore, children conceptualized organically living entities more similarly in terms of psychology and intelligence compared to technological entities [see score correlations (II) × (III) in Table 2], because score correlations of the former entities (score correlation range: 0.47 < *r* < 0.63) were consistently stronger compared to the latter ones (score correlation range: 0.17 < *r* < 0.47).

Repeated measure ANOVA models were used to examine whether children systematically differentiated between biology, intelligence and psychology (within-subjects factor) when conceptualizing entities. Supplementary mixed repeated measure ANOVA models (3 × 2) were used to explore potential age and gender differences.^5^ For all entities, there were significant within-subjects main effects for children’s average ontological conceptualization levels (see Table 2), and planned within-subjects contrasts confirmed there were significant differences between all three ontological dimensions for all entities (with humans’ biology and psychology scores, and dolls’ biology and intelligence scores being the only exceptions due to non-significant differences). This suggests children systematically differentiated between biology, intelligence and psychology when conceptualizing technological entities. For drones, there was also a significant between-subjects main effect, *F*(1, 141) = 8.65, *p* < 0.01, and an interaction effect for children’s age, *F*(1.62, 227.84) = 7.25, *p* < 0.01, as older children (9–11 years) conceptualized the entity to have more intelligence compared to younger children (7–8 years).^6^ For DVAs, there was a significant interaction effect for children’s age, *F*(1.57, 221.44) = 6.95, *p* < 0.01, with older children conceptualizing the entity to have more intelligence compared to younger children. For all entities, there were no significant between-subjects or interaction effects with children’s gender. We also explored potential relationships between ontological scores and parents’ education and age (each measured with six ordinal categories) using Spearman’s rank correlation coefficient *ρ,* and only three of the 54 correlations were significant but also weak (*ρ* < 0.3) and without any meaningful pattern.

Lastly, children’s ontological conceptualizations of technological entities were not primarily driven by anthropomorphic appearances: for humanoid robots—the only visually anthropomorphized technology used in the study—children’s average conceptualizations of intelligence (*M* = 2.01, SD = 1.50) and psychology (*M* = 0.85, SD = 1.15) did not significantly differ from the level of the closest non-anthropomorphized technological entity in each ontological dimension (computers for intelligence, *M* = 2.11, SD = 1.45; DVAs for psychology, *M* = 0.78, SD = 1.06). For biology, there was a significant difference between humanoid robots (*M* = 0.52, SD = 0.80) and drones (*M* = 0.40, SD = 0.65), *t*(142) = 2.25, *p* < 0.05, but, at closer examination, this difference was only significant for younger children, *t*(90) = 2.61, *p* < 0.05, and not for older children *t*(51) = 0.39, *p* > 0.05. In addition, humanoid robots’ biology, intelligence and psychology scores were moderately (0.40 < *r* < 0.50) correlated with respective scores of all other technological entities (see Appendix Table 6).

### Exploring associations between children’s DVA-exposure and ontological conceptualization patterns

For each child, a continuous DVA-exposure score was computed (i.e., sum of all exposure points, ranging from 0 to 7) based on parental responses from part A of the survey. Following Bernstein and Crowley (2008), a median split of exposure scores was used to create two sub-groups: children with DVA-exposure scores below the median value of 5 (i.e., score range 0–5) were assigned to the *lower* exposure group (*n* = 87), and children above the median value (i.e., score range 6–7) were assigned to the *higher* exposure group (*n* = 56).Since a total number of 39 children had a DVA-exposure score of 5, which is equal to the median value, the median split did not result in identical group sizes. To make the analysis as exhaustive as possible, we used both median split group comparisons as well as DVA-exposure scores if possible.

#### Exploring DVA-exposure sub-groups and associations with technological affinity

In the higher DVA-exposure group (*M* = 8.38 years, SD = 1.14 years), 55% of the children were boys (*n* = 31) and 45% girls (*n* = 25), and in the lower DVA-exposure group (*M* = 8.31 years, *SD* = 1.37), 54% of the children were boys (*n* = 47) and 46% girls (*n* = 40). A chi-square test revealed no significant relationship between gender and DVA-exposure group, *χ*^2^(1,143) = 0.02, *p* > 0.05, indicating that boys were *not* more likely than girls to be in the higher exposure group. According to 2 (DVA-exposure group) × 2 (gender) two-way ANOVA results (equal variances assumed according to Levene’s test of equality of variances, *F*(3, 139) = 2.52, *p* > 0.05), children’s age did not significantly differ with respect to DVA-exposure groups, *F*(1, 139) = 0.27, *p* > 0.05, with respect to gender, *F*(1, 139) = 0.06, *p* > 0.05, and with respect to the interaction between DVA-exposure groups and gender, *F*(1, 139) = 3.71, *p* > 0.05 (see also Table 3 for mean level patterns). Furthermore, and in contrast to Bernstein and Crowley’s (2008) original study, exposure scores were also not correlated with age in the total sample, *r*(141) =  − 0.12, *p* > 0.05 (a moderate negative correlation with age was only found for boys, *r*(76) =  − 0.32, *p* < 0.01).

**Table 3:** Children’s age (grouped by gender and DVA-exposure)

Controls	Total sample (*n* = 143)			Higher DVA-exposure (*n* = 56)			Lower DVA-exposure (*n* = 87)		
	*M*	*n*	(SD)	*M*	*n*	(SD)	*M*	*n*	(SD)
Boys	8.37	78	(1.25)	8.06	31	(1.00)	8.57	47	(1.36)
Girls	8.34	65	(1.19)	8.52	25	(1.16)	8.23	40	(1.21)
Total	8.36	143	(1.22)	8.27	56	(1.09)	8.41	87	(1.30)

Table 3 shows the mean values (*M*) of children’s age for different categories, respective standard deviations (SD) and the number of observations in each category (*n*). According to *t* tests, mean differences between higher and lower DVA-exposure were not significant for boys, girls and in the total sample. No missing values in the sample

In the higher DVA-exposure group, most parents (71%) had either undergraduate (*n* = 24) or postgraduate (*n* = 16) college degrees, which was similar to the lower DVA-exposure group in which 89% had either undergraduate (*n* = 46) or postgraduate (*n* = 31) college degrees. However, Spearman’s rank correlation coefficient *ρ* did show a significant but weak rank correlation between girls’ DVA-exposure and parental education levels, *ρ*(63) = 0.26, *p* < 0.05, suggesting that higher levels of parental education were positively associated with the presence of DVAs in girls’ home and childhood environments. Furthermore, 54% of parental respondents in the higher DVA-exposure group reported to be the child’s mother (*n* = 30), which is similar to the relative share of mothers in the lower exposure group (54%, *n* = 47) (see online supplementary materials for full overview of parental characteristics).

Based on the child-adjusted TAQ, an overall technological affinity score was computed for each child (*M* = 2.45, *SD* = 0.49), as well as sub-scores for children’s positive attitude (*M* = 3.05, *SD* = 0.66) towards technology, children’s reversely coded negative attitude (*M* = 1.63, *SD* = 0.94) towards technology, children’s excitement (*M* = 2.95, SD = 0.70) about technology, and their technological *competency* (*M* = 2.87, SD = 0.77). In the total sample, children’s overall technological affinity,* r*(141) = 0.21, *p* < 0.05, positive attitude*, r*(141) = 0.26, *p* < 0.01, and excitement,* r*(141) = 0.17, *p* < 0.05, were weakly correlated with DVA-exposure scores, but, at closer examination, all three correlations were only significant for boys (technological affinity: *r*(76) = 0.29, *p* < 0.05; positive attitude:* r*(76) = 0.33, *p* < 0.01; excitement: *r*(76) = 0.25, *p* < 0.05). This is in line with *t* test results suggesting boys in the higher DVA-exposure sub-group had significantly higher mean levels of overall technological affinity*, t*(76) = 3.25, *p* < 0.01, positive attitude, *t*(76) = 2.80, *p* < 0.01, and excitement,* t*(76) = 2.11, *p* < 0.05, while there were no significant differences between DVA-exposure sub-groups for girls.

In summary, children with lower and higher DVA-exposure were mostly comparable regarding their technological affinity and basic demographic characteristics, the only exception being a slight association between boys’ DVA-exposure and technological affinity. Therefore, comparing children with different levels of DVA-exposure in their home and childhood environments can be considered an empirical comparison in its own right, rather than a spurious comparison of other underlying characteristics between respective families and their children. Based on this comparability, the remaining analysis focused on associations between children’s DVA-exposure and ontological conceptualization patterns (while still controlling for technological affinity, demographic characteristics and further interactions among these variables).

#### DVA-exposure, technological affinity, and ontological conceptualizations patterns

For the remaining analysis, biology, intelligence, and psychology scores were averaged across organically living entities (humans, cats, plants) and technological entities (DVAs, smartphones, drones, computers, robots). In line with this study’s above-discussed theoretical framing, this allowed us to conduct a high-level comparison of children’s general ontological conceptualization patterns for two meaningfully different forms of being, namely life and technology. In other words, the reason for averaging ontological scores across organically living and technological entities was not of methodological but of theoretical nature.

For organically living entities, children’s ontological conceptualization patterns were systematically associated with their technological affinity (see left half of Table 4): children with higher technological affinity tended to use more ontological qualities when conceptualizing organically living entities, and this general tendency was strongly driven by the reversely coded negative attitude sub-scale, less by the positive attitude and excitement sub-scales, and not at all by children’s technological competency. The pattern was partly similar for technological entities, but only with respect to children’s use of intelligence-related ontological qualities (see right half of Table 4). In other words, especially children who were less averse towards technology conceptualized organically living entities to have significantly more biology, intelligence and psychology, and they also conceptualized technological entities to have more intelligence. DVA-exposure shared a significant but weak correlation only with children’s psychology-related conceptualizations of technological entities, *r*(141) = 0.17, *p* < 0.05.

**Table 4:** Correlation between DVA-exposure, technological affinity, and ontological scores

Correlates	Average organically living entities			Average technological entities		
	Biology	Intelligence	Psychology	Biology	Intelligence	Psychology
	*M* = 3.20 (1.30)	*M* = 1.43 (0.94)	*M* = 2.28 (0.87)	*M* = 0.34 (0.48)	*M* = 1.82 (1.05)	*M* = 0.60 (0.73)
DVA-exposure	0.10	0.06	0.07	0.04	0.08	0.17*
Technological affinity (overall)	0.45**	0.54**	0.44**	0.01	0.38**	0.06
Technological affinity (positive attitude)	0.21**	0.31**	0.23**	− 0.01	0.13	0.01
Technological affinity (negative attitude)	0.50**	0.50**	0.48**	0.07	0.42**	0.08
Technological affinity (excitement)	0.13	0.26**	0.13	− 0.07	0.14	0.02
Technological affinity (competency)	− 0.07	− 0.03	− 0.07	− 0.10	− 0.04	− 0.01

Left half of the table shows Pearson correlations between average ontological scores of organically living entities (humans, cats, plants) and children’s DVA-exposure scores, overall technological affinity, as well as the three sub-scales of technological affinity (positive attitude, negative attitude, excitement, competency). The right half of the table shows the same Pearson correlations for technological entities (DVAs, smartphones, drones, computers, robots). *Indicates significant correlation coefficients at the 0.05 level (2-tailed). **Indicates significant correlation coefficients at the 0.01 level (2-tailed). Full sample (*n* = 143) was used for all statistics. No missing values in the sample

To examine these associations in more detail, we conducted six hierarchical regression analyses. The first three models focused on average ontological scores of organically living entities as the dependent variables (see upper part of Table 5), while the last three models focused on average ontological scores of technological entities (see lower part of Table 5). Within each hierarchical regression, children’s (I) DVA-exposure, (II) technological affinity and an (III) interaction term between (I) and (II) were added stepwise into the model (none of the interaction terms were significant). For all regressions, children’s gender and age were included as controls in the baseline model, but none of the baseline models were significant (see Appendix Tables 7 and 8 for further details).

**Table 5:** Summary of hierarchical regression models (average ontological scores)

Dependent variable	Model		*R*^*2*^	Δ*R*^*2*^	Δ*F*	*p*
Average organically living entities						
Biology	(I)	DVA-exposure	0.00	0.00	1.62	0.21
	(II)	+ Technological affinity	0.19	0.19	33.20	< 0.01
Intelligence	(I)	DVA-exposure	0.00	0.00	0.91	0.34
	(II)	+ Technological affinity	0.28	0.28	56.30	< 0.01
Psychology	(I)	DVA-exposure	0.00	0.00	1.05	0.31
	(II)	+ Technological affinity	0.19	0.19	32.77	< 0.01
Average technological entities						
Biology	(I)	DVA-exposure	0.00	0.00	0.29	0.59
	(II)	+ Technological affinity	0.00	0.00	0.01	0.92
Intelligence	(I)	DVA-exposure	0.01	0.01	1.60	0.21
	(II)	+ Technological affinity	0.15	0.14	21.96	< 0.01
Psychology	(I)	DVA-exposure	0.03	0.03	4.92	< 0.05
	(II)	+ Technological affinity	0.03	0.00	0.05	0.87

Upper part of the table summarizes regression results for variables influencing children’s average attributions of biology, intelligence and psychology to organically living entities (humans, cats, plants). Lower part of the table summarizes results for technological entities (DVAs, smartphones, drones, computers, robots). Variable (I) refers to children’s DVA-exposure score, and variable (II) refers to children’s overall technological affinity. For all regression models, children’s gender and age were included as controls in the baseline model (results of baseline models not reported in the table; none of the baseline models were significant). *R*^2^ and Δ*R*^2^ refer to adjusted *R*^2^ values*.* Full sample (*n* = 143) was used for all regressions. No missing values in the sample. See Appendix Tables 7 and 8 for further details on regression results

The regression results generally confirmed the previous pattern: for organically living entities, children’s technological affinity explained significant proportions of the variance in biology (19%), intelligence (28%) and psychology (19%) scores, with neither DVA-exposure nor the interaction between both factors playing a significant role in any of the models. For technological entities, children’s technological affinity explained a significant proportion of the variance in intelligence scores (14%), while DVA-exposure explained a significant but small proportion of the variance in psychology scores (3%).

In a supplementary series of hierarchical regression analyses (results not reported here), we could confirm that, for organically living entities, the predictive effects of technological affinity were mostly driven by the positive attitude and negative attitude sub-scales, while for technological entities, the predictive effect was driven by the negative attitude sub-scale only. We also explored potential interaction effects between children’s gender and age, on the one hand, and DVA-exposure and technological affinity, on the other hand, as well as interaction effects between parental education levels, on the one hand, and DVA-exposure and technological affinity, on the other hand. There were no significant and meaningful patterns in any of the models.

#### Children’s ontological discriminations between technological and living entities

The above analysis focused on children’s average use of biology, intelligence and psychology to conceptualize organically living entities, on the one hand, and technological entities, on the other hand. To compare how a child conceptually discriminated between these two groups of entities, we examined relative within-child differences in ontological conceptualisation patterns—relative in comparison to average ontological conceptualisation patterns of children in the sample—and to what extent these were associated with DVA-exposure and technological affinity. In other words, in this last part of the analysis, the main focus was to examine whether some children (e.g. children with higher DVA-exposure) placed technological entities and organically living entities further away from each other (in terms of respective deviances from ‘the average child’s’ ontological conceptualisations) compared to other children (e.g. children with lower DVA-exposure).

To examine this, the following approach was taken: first, ontological average scores of organically living and technological entities were *z*-score standardised at the entity group level (e.g. *z*-score standardisation of the average biology score of organically living entities, *z*-score standardisation of the average biology score of technological entities etc.), therefore yielding six standardised scores with a common mean of zero, a common standard deviation of one, but different underlying distributions.^7^ Second, to measure how a child conceptually discriminated between organically living and technological entities based on his/her relative deviance from average ontological conceptualisation patterns in the sample, absolute values of ontological score differences were computed for biology, intelligence and psychology (e.g. absolute value of the difference between the standardised average intelligence score of organically living entities and the standardised average intelligence score of technological entities).^8^ In other words, based on a child’s relative deviance from average ontological conceptualisation patterns in the sample, these difference scores reflected ‘how far apart’ a child placed technological entities and organically living entities in the common ontological space of biology, intelligence and psychology. In this sense, a child with a relatively higher difference score has placed technological entities and organically living entities further away from each other (in terms of respective deviances from ‘the average child’s’ ontological conceptualisations) compared to a child with a lower ontological difference score (see Appendix Table 9 for an exemplary visualization).

To analyze these difference scores, three types of repeated measures ANOVA/ANCOVA models were conducted. Repeated measures ANOVA/ANCOVA models were preferred over simple one-, two- or three-way models, because ontological scores were assumed to be nested in individuals (i.e., individuals’ overall ontological understanding of ‘life’). In other words, when it came to the question how children conceptually discriminated between both groups of entities (i.e., organically living and technological entities), repeated measures ANOVA/ANCOVA models applied to ontological difference scores allowed us to do both, analyze children’s ontological discrimination and consider the nested structure of the data.

**Fig. 2:**
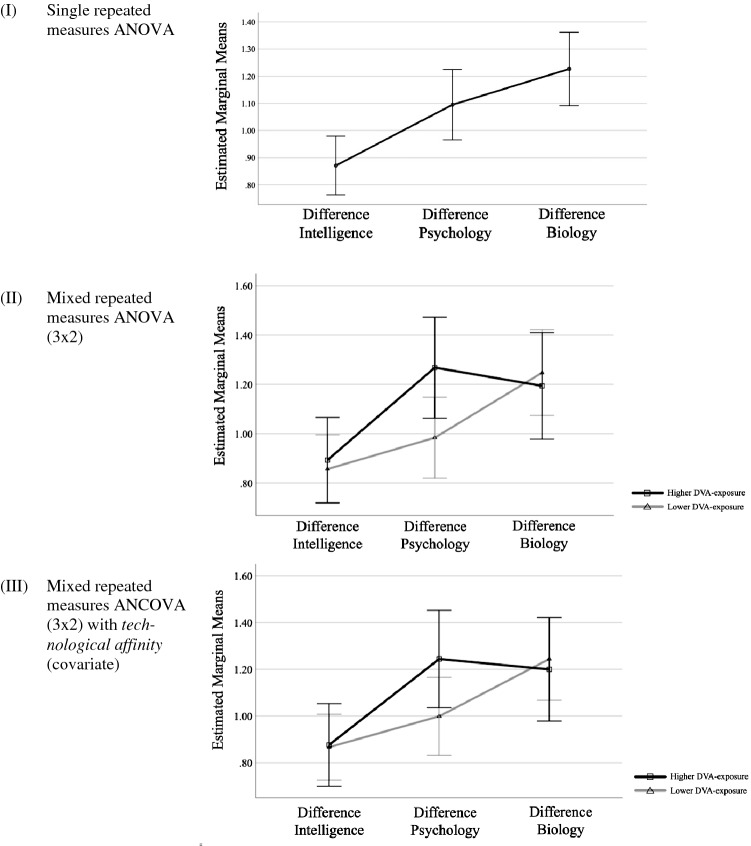
Repeated measures ANOVA/ANCOVA for ontological score differences. Notes. Figures show estimated marginal means from a repeated measures ANOVA/ANCOVA with ontological score differences between organically living entities (humans, cats, plants) and technological entities (DVAs, smartphones, drones, computers, robots) as within-subjects factors, and, for figure (II) and (III), DVA-exposure as between-subjects factors (lower vs. higher DVA-exposure). Each figure stands for a separate ANOVA/ANCOVA model. The error bars show 95% confidence intervals of estimated marginal means. Average ontological scores of organically living and technological entities were z-score standardized at the group level before the analysis. In figure (III), the covariate (technological affinity) is evaluated at the following value:* M*=2.45. Full sample (*n*=143) was used for all models. No missing values in the sample

First, a single repeated measures ANOVA was conducted, with the three difference scores as a within-subjects factor [see (I) in Fig. 2]. There was a significant within-subjects main effect, *F*(2, 284) = 8.86, *p* < 0.01, and planned within-subjects contrasts showed significant differences between intelligence and psychology (with the intelligence difference score having the lowest mean value), but no significant differences between psychology and biology. In other words, children’s relative deviances from the sample mean between organically living and technological entities were less strong with respect to intelligence (as indicated by lower mean value of the difference score) and stronger with respect to psychology as well as biology (as indicated by higher mean values of the difference scores).

Second, a mixed repeated measures ANOVA (3 × 2) was conducted, with the three difference scores as a within-subjects factor, and children’s DVA-exposure as a binary between-subjects factor [see (II) in Fig. 2]. In line with previous results, the analysis revealed a significant within-subjects main effect, *F*(2, 282) = 8.40, *p* < 0.01, but no significant within-subjects effect for the interaction term between difference scores and DVA-exposure, *F*(2, 282) = 2.01, *p* > 0.05, and also no significant between-subjects effect for DVA-exposure itself, *F*(1, 141) = 1.22, *p* > 0.05. At the main effect level, planned within-subjects contrasts showed significant differences between intelligence and psychology (with intelligence difference scores having the lowest mean value), but no significant differences between psychology and biology. Since mean value differences between higher and lower DVA-exposure appeared to be particularly strong for psychology, additional *t* tests were conducted. The results confirmed there were no significant differences in differences between DVA-exposure groups with respect to intelligence, *t*(141) =  − 0.33, *p* > 0.05, or biology, *t*(141) = 0.38, *p* > 0.05, but with respect to psychology, *t*(141) =  − 2.14, *p* < 0.05. In other words, for children with higher and lower DVA-exposure, relative deviances from the sample mean between organically living and technological entities were not significantly different with respect to intelligence and biology. But, compared to children with lower DVA-exposure, children with higher DVA-exposure discriminated relatively more between organically living and technological entities with respect to psychology (as indicated by higher mean values of psychology difference scores, therefore reflecting stronger relative deviances from the sample mean between organically living and technological entities).

Third, a mixed repeated measures ANCOVA (3 × 2) was conducted, with the three difference scores as a within-subjects factor, children’s DVA-exposure as a binary between-subjects factor, and children’s overall technological affinity as an additional covariate [see (III) in Fig. 2]. Although the pattern was similar compared to the previous model, there was no significant within-subjects main effect, *F*(2, 280) = 1.10, *p* > 0.05, no significant within-subjects effect for the interaction term between difference scores and DVA-exposure, *F*(2, 280) = 1.47, *p* > 0.05, and also no significant between-subjects effect for DVA-exposure itself, *F*(1, 140) = 0.74, *p* > 0.05. In this model, planned within-subjects contrasts showed no significant differences between any of the difference scores.

To confirm these results, we applied the previous hierarchical regression models (see Table 5) to the three difference scores. The results were partly consistent in the sense that children’s DVA-exposure (higher vs. lower) was only significantly associated with psychology difference scores, but the association became insignificant after controlling for children’s technological affinity (see Appendix Tables 10, 11, 12). In addition, all regression models showed significant associations between children’s gender and intelligence difference scores, even after controlling for children’s DVA-exposure and technological affinity. According to these results, boys discriminated relatively more (compared to girls) between organically living and technological entities with respect to intelligence (as indicated by higher mean values of intelligence difference scores, therefore reflecting stronger relative deviances from the sample mean between organically living and technological entities). Lastly, we also explored potential interaction effects between children’s gender, children’s age and parental education levels, on the one hand, and DVA-exposure and technological affinity, on the other hand, but there were no significant and meaningful patterns.

Taken together, these results suggest children, on average, discriminated relatively less between organically living and technological entities with respect to intelligence, and more with respect to psychology as well as biology (as indicated by comparisons of relative deviances from the sample mean between organically living and technological entities). However, this pattern changes when taking children’s DVA-exposure and technological affinity into account. In particular, there was some evidence that, compared to children with lower DVA-exposure, children with higher DVA-exposure discriminated relatively more between organically living and technological entities on the basis of psychology (as indicated by comparisons of relative deviances from the sample mean between organically living and technological entities), but the effect was not strong enough to be significant when children’s technological affinity was taken into account as well.

## Discussion

Our study makes several contributions to the literature, which are discussed in the following.

### Children’s general ontological conceptualization patterns

Even without scientists agreeing on what exactly life is (Westall and Brack 2018), people have an intuitive understanding of what it means (Zimmer 2021), and for any given entity, one would expect this intuitive judgement (living vs. non-living) to be consistent with other ontological qualities used to conceptualize the same entity (Gelman 1988). This is confirmed by the current study, showing how children’s ontological conceptualizations of organically living entities closely draw on intelligence-related *and* psychological qualities, further suggesting children associate the ontological quality of being intelligent with the ontological necessity of being psychological for prototypical forms of life (e.g., humans, animals, plants). In contrast, and in line with previous research showing how children selectively use ontological qualities when conceptualizing technological entities (e.g., Beran et al. 2011; Bernstein and Crowley 2008; Hughes et al. 1987; Jipson and Gelman 2007; Kahn et al. 2006, 2012; Melson et al. 2005; Okita and Schwartz 2006; Saylor et al. 2010; Scaife and Van Duuren 1995), children’s conceptualizations of technological entities were less uniform with respect to all ontological dimensions in the current study. For example, children’s use of psychological qualities to conceptualize technology is closely associated with their use of biological qualities, while children’s use of intelligence-related qualities seems to be much less reliant on biology. In that sense, and partly in line with Bernstein and Crowley’s (2008) study with a younger sample, children may generally perceive intelligence to be a more common ontological ground of life and technology, while the common ground of psychology is only established for those children who also perceive a biological overlap between life and technology. This is also reflected in children’s relative ontological discriminations (in terms of relative deviation patterns from the sample mean), which suggest children perceive the ontological quality of being intelligent as a less differentiating factor between life and technology compared to the quality of being biological or psychological.

One straight-forward interpretation of this finding would be that children in middle childhood still confuse ‘true’ ontological boundaries between life, such as humans, and technology, such as robots (e.g., van Straten et al. 2020). Following Bernstein & Crowley’s (2008) discussion, an alternative interpretation would be the following: children’s understanding of their increasingly technologized home and childhood environments accommodate both, (1) how today’s technology is programmed to perform cognitive tasks which would demonstrate the possession of intelligence when performed by organically living entities, such as humans (e.g., answering knowledge-based questions with higher levels of accuracy and speed; Festerling and Siraj 2020), and (2) how this same technology still differs in many respects to more psychological and biological forms of life—especially humans with their advanced conversational comprehension (e.g., Xu et al. 2021), their common sense and creativity (e.g., Festerling and Siraj 2020), their disposition to laugh (e.g., Yip et al. 2019), or their ability to answer psychological or biological questions (e.g., Oranç and Küntay 2020). This interpretation of our findings draws on Kahn et al. (2006, 2007, 2009, 2011, 2012) NOCH and its implicit assumption that there is no a priori definable and metaphysically ‘true’ end-state for how one should conceptualize technology vis-à-vis organically living entities. Therefore, our study contributes to the literature by showing how today’s children systematically disentangle the ontological dimensions of psychology and intelligence when conceptualizing entities of distinct kinds in their home and childhood environments. But, as previous research has also shown, this does not mean children entirely refrain from using psychological qualities when conceptualizing technological entities, such as DVAs (e.g., Garg and Sengupta 2020; Girouard‐Hallam et al. 2021; Hoffman et al. 2021), smart toys (e.g., Turkle 2017), or robots (e.g., van Straten et al. 2020). However, this study was able to show that, across a broad range of technological and organically living entities, children’s relative ontological conceptualisation patterns (in terms of relative deviation patterns from the sample mean) still tend to discriminate more rigorously on the basis of psychology and biology, and less on the basis of intelligence. But the second question which yet remains to be answered is whether exposure to certain kinds of technology could prompt children to develop more nuanced understandings of prototypical entities in their environments.

### Associations between children’s DVA-exposure and ontological conceptualization patterns

Children’s exposure to technology within their home and childhood environments is a matter of degree, not a yes/no phenomenon (Gaudiello et al. 2015). This applies to DVAs as well: families and their children may use DVAs across the entire household for various different purposes and establish very stable usage routines over time (Ammari et al. 2019; Garg and Sengupta 2020; Lopatovska and Williams 2018; Porcheron et al. 2018; Sciuto et al. 2018), which can even culminate in DVAs having a social harmonization effect similar to pets (Lee et al. 2020). To consider such degrees, the current study applied a point-based system accounting for different kinds of basic DVA-exposure which children may experience in their home and childhood environments, and, as the findings show, through this empirical lens children’s DVA-exposure continuously spreads from lower to higher levels.

Although, to the best of our knowledge, there is no empirical research (yet) suggesting DVA-exposure is unequally spread across or even within family households depending on children’s or parents’ demographic characteristics, the current study does show some positive associations mediated through parental education levels and favoring girls’ overall DVA-exposure. Therefore, DVAs may be spread differently among today’s children compared to, for example, robotic technologies, which have previously been found to be biased against girls in younger samples (e.g., Bernstein and Crowley 2008). Another noteworthy finding in this context is that children’s DVA-exposure is only weakly associated with their general attitudes towards, or competency with, technology. This is in line with previous arguments in the literature suggesting voice-enabled technologies may be able to reduce common interaction barriers for children to engage with technology (e.g., Lovato et al. 2019; Yuan et al. 2019), therefore making DVAs appealing to various levels of technological affinity. Taken together, comparing children with different levels of naturally occurring DVA-exposure is not a spurious comparison of underlying child- or family characteristics, or, in other words, a comparison which only reflects more general differences between respective families and their children. However, we also acknowledge that the socio-technical spread of DVAs in today’s home and childhood environments may indeed be a more complex than the account given of it in the current study.

For children’s general ontological conceptualizations of organically living as well as technological entities, the current study suggests children’s technological affinity plays a far more important role than their DVA-exposure. Given personality-related constructs on people’s attitudes towards technology have generally been found to be broadly associated with various other characteristics in adults (e.g., Anthony et al. 2000; dos Santos and Santana 2018; Horstmann et al. 2018; Korukonda 2005, 2007; Nitsch and Glassen 2015; Powell 2013; Saleem et al. 2011) as well as children (e.g., Baloğlu and Çevik 2008; Chou 2001; Cooper 2006; King et al. 2002; Rees and Noyes 2007; Todman and Lawrenson 1992; Todman and Monaghan 1994), this may not be surprising at first sight. However, within the construct of technological affinity, it is especially the aversion towards technology which predicts children’s conceptualization patterns—not their technological positivity or competency. In other words, children who are less averse towards technology conceptualize organically living entities to have more biological, intelligence-related, and psychological qualities, and they also conceptualize technological entities to have more intelligence-related qualities. This finding is robust to various controls (e.g., children’s age and gender, parental education) and thereby constitutes an important contribution to existing research, because it suggests children’s stance towards technology is not only associated with their perceptions of technology in and of itself (e.g., Beran et al. 2011) but also with their perceptions of organically living entities (e.g., Gaudiello et al. 2015). In conjunction with the previous discussion, and in complementation to recent discussions in the literature arguing that positive attitudes towards state-of-the-art technology become more prevalent in empirical research (Naneva et al. 2020), one interpretation of this finding would be that being less averse towards technology still constitutes a threshold for children to discriminate more rigorously between life and technology on the basis of psychology and biology, and less on the basis of intelligence.

Yet, when it comes to children’s relative ontological discriminations (in terms of relative deviation patterns from the sample mean), there is some evidence that, compared to children with lower DVA-exposure, children with higher DVA-exposure discriminate relatively more between life and technology on the basis of psychology. Therefore, and despite the positive association between children’s DVA-exposure and their pronounced use of psychology to conceptualize technological entities, it is far from clear that such tendencies blur ontological boundaries between life and technology from children’s perspective. With this finding, our study substantiates Bernstein and Crowley (2008) in the sense that with more exposure to technology, psychology becomes the fulcrum of children’s ontological differentiations between technological and organically living entities. But our study also suggests that with more exposure to technology, children’s perceptions of psychology may become more pronounced. Although this is generally in line with previous research showing how children may perceive technological entities as particularly reliable and trustworthy social engagement partners (e.g., Turkle, 1984/2005, 2017) offering instant social gratification (Festerling and Siraj 2020; Oranç and Ruggeri 2021) and even allowing for feelings of closeness (e.g., van Straten et al. 2020), the question remains why psychology interacted so differently with technological exposure in both studies. We cannot answer this conclusively, but—apart from age-related developmental differences between children in both studies—we hypothesize the reason could lie in the different natures of technological exposure under investigation. Research on children’s exposure to robotic technologies (e.g. Bernstein and Crowley 2008; Gaudiello et al. 2015; van Straten et al. 2020) tends to focus on ‘educationalized’ ways of engaging with technology (e.g., acquiring functional knowledge about robots’ inner working mechanisms, learning how to build and program robots). Arguably, such exposure implicitly lends itself to less psychological ways of conceptualizing technology by emphasizing the ‘true’ ontological chasm between ‘us and them’ (MacDorman et al. 2009). In contrast, children’s real-world DVA-exposure, as investigated in the current study, focuses on children’s overall engagement opportunities to experience DVAs within their real-world home and childhood environments—and not necessarily how much they are formally educated about DVAs. This may leave more scope for psychological ways of conceptualizing technology, not only because voice-only communication has previously been found to enhance psychological connections between social engagement partners (Kraus 2017), but also because children with higher DVA-exposure may have a more intense and direct social experience of how today’s technology can emulate certain qualities of human psychology (Festerling and Siraj 2020).

However, in this context, a major methodological limitation which our study inherits from Bernstein and Crowley (2008) is that we do not know whether children used certain ontological qualities—especially psychological qualities—according to what they thought of as metaphysically ‘true’ (e.g., ‘Even though it pretends, I know Alexa cannot really sense how I feel!’), or, in contrast, in terms of what they believe or want to be true (e.g., ‘I hope Alexa can sense how I feel!’). This is something future research could address by considering children’s own psychological motives (e.g., sociality motivation, Epley et al. 2007) and how these relate to their individual DVA-exposure and ontological conceptualization patterns. To do so from a methodological perspective, future research should consider going beyond the simple analysis of observed variables and model children’s ontological conceptualization patterns together with their socio-emotional motives as latent item-based variables within a structural equation modeling framework. This would also allow to address differences within ontological dimensions by analyzing children’s conceptualization patterns at the item-level.

Furthermore, according to our study children’s technological competency is not associated with their ontological conceptualization patterns in any way. However, this absence of evidence should not be interpreted as evidence of absence. Although we examined the structural validity and reliability of the TAQ before the main analysis (see online supplementary materials), its granularity to capture nuances in how competent children are with the functionalities of today’s technology can certainly be called into question. As previous research has shown (e.g., Gaudiello et al. 2015), children’s technological competency (e.g., building and programming technology) does influence how they conceptualize technological entities, and future research could address this issue in more detail for children’s exposure to DVAs.

Lastly, another obvious limitation of our study is related to its external validity: apart from general issues related to the limited representativeness of MTurk samples (Difallah et al. 2018), the narrow age range under investigation (7–11 years) weakens the developmental comparability of our findings to studies which investigated ontological conceptualization patterns for different age ranges. For example, one would not necessarily expect an 11-year-old child in our study to conceptualize a technological entity to be alive due to self-propelled movement only, while this pattern may seem quite plausible in the context of a 4-year-old child in Bernstein and Crowley’s (2008) original study. Following Brink et al. (2019) recent developmental study on the origins of the so-called ‘uncanny valley’ phenomenon, future studies could widen the age ranges under investigation (e.g., 3–18 years) to examine how children’s ontological conceptualization patterns develop with age and depending on their DVA-exposure. But one should also keep in mind that investigating the development of children’s conceptual understandings of life and technology may be heavily confounded by cohort effects and environmental trends, especially due to accelerating changes in the socio-technical environment as exemplified by DVAs (e.g., Harwood and Eaves 2020). Ideally, future research could implement more complex sequential designs (e.g., Schaie 1994) to disentangle these different effects in the context of DVAs and to identify their overall impact on human development.

### Electronic supplementary material

Below is the link to the electronic supplementary material.

Supplementary file1 (DOCX 133 kb)

## Figures and Tables

**Fig. 3:**
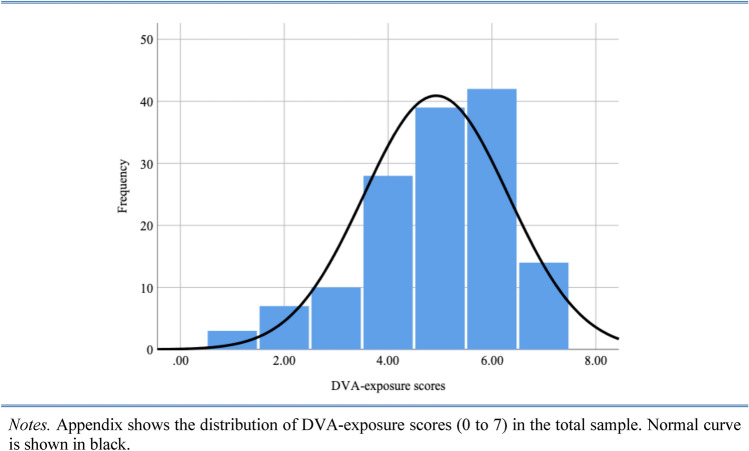
Distribution of DVA-exposure scores

## Data Availability

Due to the nature of this research, participants of this study did not agree for their data to be shared publicly, so supporting data is not available.

## Appendix

See Fig. 3, Tables 6, 7, 8, 9, 10, 11, 12.

**Table 6:** Correlation of ontological scores within and between technological entities

Ontological scores	(1)			(2)			(3)			(4)			(5)		
	(a)	(b)	(c)	(a)	(b)	(c)	(a)	(b)	(c)	(a)	(b)	(c)	(a)	(b)	(c)
(1) DVAs															
(a) Biology	1														
(b) Intelligence	0.18^*^	1													
(c) Psychology	0.44^**^	0.37**	1												
(2) Computers															
(a) Biology	0.50**	0.13	0.32**	1											
(b) Intelligence	-0.05	0.41**	0.19**	0.02	1										
(c) Psychology	0.29**	0.15	0.51**	0.32**	0.30**	1									
(3) Smartphones															
(a) Biology	0.47**	0.07	0.24**	0.44**	0.11	0.22**	1								
(b) Intelligence	0.10	0.50**	0.22**	0.21**	0.73**	0.27**	0.22**	1							
(c) Psychology	0.33**	0.22**	0.62**	0.31**	0.30**	0.64**	0.39**	0.28**	1						
(4) Humanoid robots															
(a) Biology	0.37**	0.20*	0.35**	0.28**	0.27**	0.29**	0.35**	0.20*	0.35**	1					
(b) Intelligence	-0.09	0.42**	0.06	0.03	0.53**	0.11	-0.05	0.46**	0.04	0.25**	1				
(c) Psychology	0.15	0.28**	0.48**	0.09	0.41**	0.41**	0.15	0.31**	0.42**	0.44**	0.47**	1			
(5) Drones															
(a) Biology	0.49**	0.10	0.39**	0.23**	0.11	0.26**	0.54**	0.15	0.38**	0.60**	0.02	0.34**	1		
(b) Intelligence	0.06	0.25**	0.15	0.03	0.28**	0.25**	0.13	0.34**	0.22**	0.15	0.43**	0.42**	0.11	1	
(c) Psychology	0.36**	0.13	0.49**	0.27**	0.20*	0.49**	0.33**	0.26**	0.61**	0.36**	0.07	0.45**	0.47**	0.17*	1

Material shows correlation of ontological scores within as well as across technological entities in the total sample (*n* = 143)

*Indicates significant correlation coefficients at the 0.05 level (2-tailed)

**Indicates significant correlation coefficients at the 0.01 level (2-tailed)

**Table 7:** Coefficients of hierarchical regression models (average ontological scores of organically living entities)

	Independent variables	Baseline model					Model (I)					Model (II)					Model (III)				
		*B*	SE	*β*	*t*	*p*	*B*	SE	*β*	*t*	*p*	*B*	SE	*β*	*t*	*p*	*B*	SE	*β*	*t*	*p*
Biology	Gender	0.20	0.22	0.08	0.92	0.36	0.22	0.22	0.08	1.01	0.32	0.13	0.20	0.05	0.67	0.50	0.13	0.20	0.05	0.63	0.53
	Age	0.04	0.09	0.03	0.40	0.69	0.05	0.09	0.05	0.55	0.58	0.03	0.08	0.03	0.39	0.70	0.03	0.08	0.03	0.36	0.72
	DVA-exposure						0.10	0.08	0.11	1.27	0.21	0.01	0.07	0.01	0.11	0.92	− 0.10	0.42	− 0.11	− 0.24	0.81
	Tech. affinity											1.19	0.21	0.45	5.76	< 0.00	0.95	0.94	0.36	1.01	0.31
	Interaction																0.04	0.17	0.16	0.26	0.80
Intelligence	Gender	− 0.01	0.16	− 0.01	− 0.08	0.94	0.00	0.16	0.00	− 0.01	0.99	− 0.08	0.13	− 0.04	− 0.59	0.56	− 0.08	0.14	− 0.04	− 0.60	0.55
	Age	0.10	0.06	0.14	1.62	0.11	0.22	0.06	0.15	1.73	0.09	0.10	0.05	0.12	1.75	0.08	0.10	0.06	0.12	1.72	0.09
	DVA-exposure						0.05	0.06	0.08	0.96	0.34	− 0.03	0.05	-0.04	− 0.55	0.58	− 0.07	0.28	− 0.10	− 0.24	0.81
	Tech. affinity											1.04	0.14	0.55	7.50	< 0.00	0.95	0.63	0.50	1.51	0.13
	Interaction																0.02	0.11	0.08	0.15	0.88
Psychology	Gender	0.02	0.15	0.01	0.15	0.88	0.03	0.15	0.02	0.22	0.83	− 0.03	0.13	− 0.02	− 0.20	0.84	− 0.02	0.13	− 0.01	− 0.18	0.86
	Age	0.09	0.06	0.12	1.47	0.14	0.10	0.06	0.13	1.58	0.12	0.08	0.05	0.12	1.53	0.13	0.08	0.06	0.12	1.53	0.13
	DVA-exposure						0.05	0.05	0.09	1.03	0.31	− 0.01	0.05	− 0.01	− 0.15	0.88	0.02	0.28	0.03	0.07	0.94
	Tech. affinity											79	0.14	0.44	5.72	< 0.00	0.85	0.63	0.48	1.36	0.18
	Interaction																− 0.01	0.11	− 0.06	− 0.10	0.92

Table shows unstandardized regression coefficients (*B*), standard errors (SE), standardized regression coefficients (*β*)*, t *values (*t*) and *p* values (*p*) for all independent variables and across all four hierarchical regression models. ‘Interaction’ refers to the interaction term between DVA-exposure and Tech. affinity (technological affinity). The dependent variables were average biology, intelligence or psychology scores of organically living entities (humans, cats, plants). Full sample (*n* = 143) was used for all regressions. No missing values in the sample

**Table 8:** Coefficients of hierarchical regression models (average ontological scores of technological entities)

	Independent variables	Baseline model					Model (I)					Model (II)					Model (III)				
		*B*	SE	*β*	*t*	*p*	*B*	SE	*β*	*t*	*p*	*B*	SE	*β*	*t*	*p*	*B*	SE	*β*	*t*	*p*
Biology	Gender	− 0.01	0.08	− 0.01	− 0.12	0.91	− 0.01	0.08	− 0.01	− 0.08	0.94	− 0.01	0.08	− 0.01	− 0.07	0.94	− 0.01	0.08	− 0.01	− 0.11	0.92
	Age	0.01	0.03	0.02	0.22	0.83	0.01	0.03	0.02	0.28	0.78	0.01	0.03	0.02	0.28	0.78	0.01	0.03	0.02	0.25	0.80
	DVA-exposure						0.02	0.03	0.05	0.54	0.59	0.02	0.03	0.05	0.54	0.59	− 0.03	0.18	− 0.09	− 0.18	0.85
	Tech. affinity											− 0.01	0.09	− 0.01	− 0.10	0.92	− 0.12	0.39	− 0.12	− 30	0.77
	Interaction																0.02	0.07	0.20	0.28	0.78
Intelligence	Gender	0.12	0.18	0.06	0.68	0.50	0.13	0.18	0.06	0.76	0.45	0.08	0.16	0.04	0.46	0.65	0.06	0.17	0.03	0.38	0.70
	Age	0.13	0.07	0.15	1.77	0.08	0.14	0.07	0.16	1.92	0.06	0.13	0.07	0.15	1.87	0.06	0.12	0.07	0.14	1.80	0.07
	DVA-exposure						0.08	0.06	0.11	1.27	0.21	0.02	0.06	0.02	0.29	0.77	− 0.17	0.35	− 0.23	− 0.49	0.62
	Tech. affinity											0.80	0.17	0.37	4.69	< 0.00	0.38	0.77	0.18	0.49	0.62
	Interaction																0.08	0.14	0.35	0.55	0.58
Psychology	Gender	0.19	0.12	0.13	1.53	0.13	0.21	0.12	0.14	1.70	0.09	0.20	0.12	0.14	1.68	0.10	0.21	0.12	0.14	1.68	0.10
	Age	− 0.01	0.05	− 0.01	− 0.10	0.92	0.01	0.05	0.01	0.16	0.87	0.01	0.05	0.01	0.16	0.88	0.01	0.05	0.01	0.17	0.87
	DVA-exposure						0.10	0.04	0.19	2.22	< 0.05	0.09	0.04	0.18	2.12	0.04	0.13	0.26	0.24	0.48	0.63
	Tech. affinity											0.02	0.13	0.01	0.16	0.87	0.09	0.57	0.06	0.15	0.88
	Interaction																− 0.01	0.10	− 0.08	− 0.12	0.91

Table shows unstandardized regression coefficients (*B*), standard errors (SE), standardized regression coefficients (*β*)*, t *values (*t*) and *p* values (*p*) for all independent variables and across all four hierarchical regression models. ‘Interaction’ refers to the interaction term between DVA-exposure and Tech. affinity (technological affinity). The dependent variables were average biology, intelligence or psychology scores of technological entities (DVAs, smartphones, drones, computers, robots). Full sample (*n* = 143) was used for all regressions. No missing values in the sample

**Table 9:** Exemplary visualization of ontological discrimination patterns

(i)	Exemplary visualization for a child with relatively stronger discrimination patterns (compared to child in [ii])	
(ii)	Exemplary visualization for a child with relatively weaker discrimination patterns (compared to child in [i])	

Appendix visualizes the underlying rationale of how to calculate ontological discrimination patterns. Figure (i) visualizes the fictitious example of a child with strong discrimination patterns (*∆I*_Liv-Tech_ > 1). In this example, the average intelligence level of technology for this child is *I*_Tech_ =  − 1.50 (i.e. 1.50 standard deviations below the standardized sample mean of technological entities, *M*_Tech_), and the average intelligence level of organically living entities for this child is *I*_Liv_ = 0.20 (i.e. 0.20 standard deviations above the standardized sample mean of organically living entities, *M*_Liv_). How much this child conceptually discriminates between technological entities and organically living entities on the basis of intelligence (and relative to the ‘average child’ in the sample) is indicated by the absolute value of the difference, *∆I*_Liv-Tech_ = 1.70. The interpretation for psychology (*P*) and biology (*B*) are analogous. According to the same rationale, figure (ii) visualizes the fictitious example of a child with weak discrimination patterns (*∆I*_Liv-Tech_ < 1). The threshold of *∆I*_Liv-Tech_ = 1 was randomly chosen for exemplary purposes only and does not have any implications for the analysis

**Table 10:** Summary of hierarchical regression models (ontological difference scores)

Dependent variable	Model		*R* ^2^	Δ*R*^2^	Δ*F*	*p*
Models with continuous DVA-exposure scores						
Difference biology	(I)	DVA-exposure score	0.00	0.00	0.66	0.41
	(II)	+ Technological affinity (overall)	0.00	0.00	0.19	0.66
Difference intelligence	(I)	DVA-exposure score	0.02	0.02	0.38	0.54
	(II)	+ Technological affinity (overall)	0.02	0.00	0.27	0.61
Difference psychology	(I)	DVA-exposure score	0.02	0.02	2.53	0.11
	(II)	+ Technological affinity (overall)	0.02	0.00	1.52	0.22
Models with median split DVA-exposure groups						
Difference biology	(I)	DVA-exposure group	0.00	0.00	0.08	0.78
	(II)	+ Technological affinity (overall)	0.00	0.00	0.03	0.86
Difference intelligence	(I)	DVA-exposure group	0.02	0.02	0.12	0.73
	(II)	+ Technological affinity (overall)	0.02	0.00	0.32	0.57
Difference psychology	(I)	DVA-exposure group	0.02	0.02	4.79	< 0.05
	(II)	+ Technological affinity (overall)	0.02	0.00	1.10	0.30

Table summarizes hierarchical regression models for variables influencing children’s conceptual discriminations between organically living entities (humans, cats, plants) and technological entities (DVAs, smartphones, drones, computers, robots), as indicated by their ontological difference scores. Variable (I) refers to children’s DVA-exposure score, and variable (II) refers to children’s overall technological affinity. Upper part of the table refers to regression models which are based on DVA-exposure scores. Lower part of the table refers to regression models which are based on median split DVA-exposure groups (lower vs. higher DVA-exposure). For all regression models, children’s gender and age were included as controls in the baseline model (results of baseline models not reported in the table). *R*^2^ and Δ*R*^2^ refer to adjusted *R*^2^ values*.* Full sample (*n* = 143) was used for all regressions. No missing values in the sample. See Appendix Tables 11 and 12 for further details on regression results

**Table 11:** Coefficients of hierarchical regression models (models with continuous DVA-exposure scores)

	Independent variables	Baseline model					Model (I)					Model (II)					Model (III)				
		*B*	SE	*β*	*t*	*p*	*B*	SE	*β*	*t*	*p*	*B*	SE	*β*	*t*	*p*	*B*	SE	*β*	*t*	*p*
Biology	Gender	− 0.11	0.14	− 0.07	− 0.78	0.44	− 0.10	0.14	− 0.06	− 0.72	0.47	− 0.09	0.14	− 0.06	− 0.68	0.50	− 0.11	0.14	− 0.07	− 0.81	0.42
	Age	0.08	0.06	0.12	1.48	0.14	0.09	0.06	0.13	1.57	0.12	0.09	0.06	0.13	1.58	0.12	0.08	0.06	0.12	1.46	0.15
	DVA-exposure sc						0.04	0.05	0.07	0.81	0.42	0.05	0.05	0.08	0.89	0.38	− 0.26	0.29	− 0.44	− 0.88	0.38
	Tech. affinity											− 0.06	0.14	− 0.04	− 0.44	0.66	− 0.73	0.65	− 0.44	− 1.22	0.26
	Interaction																0.12	0.12	0.72	1.05	0.29
Intelligence	Gender	0.25	0.11	0.19	2.36	< 0.00	0.26	0.11	0.20	2.39	< 0.00	0.26	0.11	0.19	2.33	< 0.00	0.26	0.11	0.20	2.36	< 0.00
	Age	0.04	0.04	0.07	0.84	0.40	0.04	0.04	0.08	0.90	0.37	0.04	0.04	0.07	0.88	0.38	0.04	0.05	0.08	0.92	0.36
	DVA-exposure sc						0.02	0.04	0.05	0.61	0.54	0.02	0.04	0.04	0.48	0.63	0.11	0.23	0.24	0.48	0.63
	Tech. affinity											0.06	0.11	0.04	0.51	0.61	0.26	0.51	0.20	0.50	0.61
	Interaction																-0.04	0.09	− 0.27	− 0.40	0.69
Psychology	Gender	0.06	0.13	0.04	0.45	0.65	0.07	0.13	0.05	0.56	0.58	0.06	0.13	0.04	0.47	0.64	0.08	0.13	0.05	0.57	0.57
	Age	0.05	0.05	0.08	0.92	0.36	0.06	0.05	0.09	1.11	0.27	0.06	0.05	0.09	1.07	0.29	0.06	0.05	0.10	1.15	0.25
	DVA − exposure sc						0.08	0.05	0.13	1.59	0.11	0.06	0.05	0.11	1.28	0.20	0.31	0.28	0.55	1.09	0.28
	Tech. affinity											0.17	0.14	0.11	1.23	0.22	0.70	0.62	0.44	1.14	0.26
	Interaction																− 0.10	0.11	− 0.61	− 0.89	0.38

Table shows unstandardized regression coefficients (*B*), standard errors (SE), standardized regression coefficients (*β*)*, t *values (*t*) and *p* values (*p*) for all independent variables and across all four hierarchical regression models. ‘DVA-exposure sc.’ refers to DVA-exposure scores*.* ‘Interaction’ refers to the interaction term between DVA-exposure and Tech. affinity (technological affinity). The dependent variables are ontological difference scores (biology, intelligence, psychology). Full sample (*n* = 143) was used for all regressions. No missing values in the sample

**Table 12:** Coefficients of hierarchical regression models (models with median split DVA-exposure groups)

	Independent variables	Baseline Model					Model (I)					Model (II)					Model (III)				
		*B*	SE	*β*	*t*	*p*	*B*	SE	*β*	*t*	*p*	*B*	SE	*β*	*t*	*p*	*B*	SE	*β*	*t*	*p*
Biology	Gender	− 0.11	0.14	− 0.07	− 0.78	0.44	− 0.11	0.14	− 0.07	− 0.77	0.44	− 0.10	0.14	− 0.06	− 0.76	0.45	− 0.14	0.14	− 0.09	− 1.02	0.31
	Age	0.08	0.06	0.12	1.48	0.14	0.08	0.06	0.12	1.46	0.15	0.08	0.06	0.12	1.46	0.15	0.07	0.06	0.11	1.33	0.18
	DVA-exposure gr						− 0.04	0.14	− 0.02	− 0.29	0.78	− 0.03	0.15	− 0.02	− 0.23	0.82	− 1.52	0.73	− 0.91	− 2.09	< 0.05
	Tech. affinity											− 0.03	0.14	− 0.02	− 0.18	0.85	− 0.36	0.21	− 0.22	− 1.68	0.10
	Interaction																0.60	0.29	0.98	2.09	< 0.05
Intelligence	Gender	0.25	0.11	0.19	2.36	< 0.05	0.25	0.11	0.19	2.34	< 0.05	0.25	0.11	0.19	2.30	< 0.05	0.25	0.11	0.19	2.28	< 0.05
	Age	0.04	0.04	0.07	0.84	0.40	0.04	0.04	0.07	0.85	0.39	0.04	0.04	0.07	0.84	0.40	0.04	0.04	0.07	0.83	0.41
	DVA-exposure gr						0.04	0.11	0.03	0.35	0.73	0.02	0.11	0.02	0.20	0.85	0.04	0.58	0.03	0.07	0.94
	Tech. affinity											0.06	0.11	0.05	0.57	0.57	0.07	0.17	0.05	0.40	0.69
	Interaction																− 0.01	0.23	− 0.02	− 0.03	0.97
Psychology	Gender	0.06	0.13	0.04	0.45	0.65	0.06	0.13	0.04	0.42	0.67	0.05	0.13	0.03	0.36	0.72	0.05	0.13	0.03	0.40	0.69
	Age	0.05	0.05	0.08	0.92	0.36	0.06	0.05	0.09	1.06	0.29	0.06	0.05	0.09	1.03	0.30	0.06	0.05	0.09	1.05	0.30
	DVA-exposure gr						0.29	0.13	0.18	2.19	< 0.05	0.26	0.14	0.16	1.86	0.07	0.47	0.70	0.29	0.67	0.51
	Tech. affinity											0.14	0.14	0.09	1.05	0.30	0.19	0.21	0.12	0.93	0.35
	Interaction																− 0.09	0.28	− 0.14	− 0.31	0.76

Table shows unstandardized regression coefficients (*B*), standard errors (SE), standardized regression coefficients (*β*)*, t *values (*t*) and *p* values (*p*) for all independent variables and across all four hierarchical regression models. ‘DVA-exposure gr.’ refers to DVA-exposure groups (based on median split), the reference group being children with lower DVA-exposure*.* ‘Interaction’ refers to the interaction term between DVA-exposure and Tech. affinity (technological affinity). The dependent variables are ontological difference scores (biology, intelligence, psychology). Full sample (*n* = 143) was used for all regressions. No missing values in the sample

## Footnotes

1 By ‘real-world exposure’ we mean children’s exposure within their own home and childhood environments, which contrasts with laboratory environments used in experimental studies (see Seaborn et al. 2021).

2 This is not to say that DVAs are the only socio-technical phenomenon which have affected home and childhood environment over the last decades (e.g., smartphones, tablets, smart watches, robots etc.). However, as we have discussed elsewhere in depth (e.g., Festerling 2020; Festerling and Siraj 2020, 2021), DVAs constitute a relevant research case in their own right and are therefore the focal point of this study.

3 Parents were instructed that they may stay in the same room while their child completes part B of the survey, but that they must allow their child to respond freely to the questions without interfering or ‘correcting’ him/her.

4 For the sake of completeness, our adjusted version of Bernstein and Crowley's (2008) original conceptualization task included one control item on entities’ ontological artificiality (‘Tick the ones that were made in factory’). However, the item was not used in the exploratory course of our data analysis and is therefore omitted from the discussion.

5 For all entities, data failed to meet the sphericity assumption as indicated by Mauchly’s test of sphericity, so the Greenhouse–Geisser correction was applied for all models.

6 This was also reflected in correlation patterns, where children’s age was significantly but weakly correlated with drones’ intelligence scores, *r*(141) = .26, *p* < .01. Other significant but weak correlations with children’s age were found for plants’ intelligence scores, *r*(141) = .22, *p* < .01, and dolls’ biology scores, *r*(141) = .27, *p* < .01. For all other 24 ontological scores across the 9 entities, there were no significant correlations with children’s age.

7 We also repeated the entire analysis in this section with *z*-score standardisations at the entity level (e.g. *z*-score standardisation of the human intelligence score, DVA-intelligence score etc.) before calculating average scores and relative differences. However, the overall patterns of the results were not meaningfully different.

8 We used absolute values of relative ontological score differences rather than real numbers (with positive and negative values), because with this analysis, we wanted to examine the magnitude of children’s relative ontological discriminations, and *not* whether children conceptualised technological entities to be more intelligent / biological / psychological than organically living entities, or vice versa (as it would have been indicated by positive and negative values). Addressing the latter question would imply a direct ranking of entities which goes beyond our data.
