# Supplementary material for: Exploring children’s exposure to voice assistants and their ontological conceptualizations of life and technology
Source: AI Soc. 2022 Oct 19:1–28. Online ahead of print. doi: 10.1007/s00146-022-01555-3 (PMC9580440; doi:10.1007/s00146-022-01555-3)
Supplement: Supplementary file 1 — Supplementary file1 (DOCX 133 kb) [file 146_2022_1555_MOESM1_ESM.docx]

| Supplementary Material I. Children’s ontological models of intelligence | | | | |
| --- | --- | --- | --- | --- |
|  | | Models of Intelligence | | |
|  |  | Model (I) | | Model (II) |
| Characteristics | | *Robot as Animal*  (*n*=13) | *Robot as Machine*  (*n*=21) | *Robot as Smart*  *Technology*  (*n*=26) |
| Exposure to technology | | Lower | Lower | Higher |
| Conceptualization patterns | *Robot biology* | Alive | Not alive | Alive / not alive |
|  | *Robot intelligence* | Higher | Lower | Higher |
|  | *Robot psychology* | Higher | Lower | Lower |
| Corresponding intelligence level of robot | | Cat | Calculator | Cat |
| *Notes.* Table summarizes Bernstein & Crowley's (2008) main findings on two different models of intelligence children applied for their ontological conceptualizations of robots. Model (I) refers to children whose conceptualizations of robotic technologies in terms of intelligence *and* psychology were consistent with their biological conceptualizations of robotic technologies (for this part of the analysis, the authors used the single item on ‘aliveness’ to determine children’s biological conceptualizations of entities). Children who applied this model of intelligence had *lower* levels of environmental exposure to robotic technologies. Model (II) refers to children whose ontological conceptualizations of robotic technologies in terms of intelligence and psychology were *not* systematically related to their biological conceptualizations. Children who applied this model of intelligence had *higher* levels of exposure to robotic technologies. Findings are based on a cross-sectional and non-random sample of children (*n*=60, age: 4–7 years) who completed a forced-choice conceptualization task (15 items, 8 entities) and a parental survey on children’s environmental exposure to robotic technologies.  *Source*. Developed from Bernstein & Crowley (2008) | | | | |

| Supplementary Material II. Parental characteristics (grouped by DVA-exposure) | | | |
| --- | --- | --- | --- |
| Characteristic | Total Sample  (*n*=143) | Higher DVA-Exposure  (*n=*56) | Lower DVA-Exposure  (*n=*87) |
| *Parental respondent* |  |  |  |
| *Mother* | 77 | 30 | 47 |
| *Father* | 66 | 26 | 40 |
| *Parental respondent age* |  |  |  |
| *24 years or younger* | 6 | 2 | 4 |
| *Between 25 and 34 years* | 70 | 27 | 43 |
| *Between 35 and 44 years* | 44 | 22 | 22 |
| *Between 45 and 54 years* | 16 | 4 | 12 |
| *Between 55 and 64 years* | 6 | 1 | 5 |
| *64 years or older* | 1 | (-) | 1 |
| *Parental respondent education* |  |  |  |
| *Less than high school* | 2 | 1 | 1 |
| *High school graduate* | 4 | 3 | 1 |
| *Undergraduate college degree* | 70 | 24 | 46 |
| *Postgraduate college degree* | 47 | 16 | 31 |
| *Professional degree* | 19 | 12 | 7 |
| *Doctorate* | 1 | (-) | 1 |
| *Notes.* Material shows frequencies of demographical characteristics grouped by children’s age, including the family relationship of the parental respondent, the parental respondent’s age, and the parental respondent’s educational background. No missing values in the sample. | | | |

| Supplementary Material III. Child-adjusted Technological Affinity Questionnaire (TAQ) | | |
| --- | --- | --- |
| TAQ sub-scales | Item label | Item |
| TAQ-Intro | (-) | We would like to know what you think of technology. Below, you find a list of sentences.  For each sentence, please tell us to what extent it applies to you by clicking: not at all 👎👎, not really 👎, a bit 🤔👍, quite a lot 👍, or very much 👍👍.  ℹ️ By technology we mean everything you can do with technological things, such as smartphones or tablets📱, laptops 💻, smart TVs 🖥️, smart watches⌚, robots 🤖 and so on. |
| (1) Negative Attitude | TAQ_Neg_1* | In my opinion technology makes daily life complicated |
|  | TAQ_Neg_2* | In my opinion there is too much technology around us |
|  | TAQ_Neg_3* | I think technology is a great threat for us as humans |
|  | TAQ_Neg_4* | I think technology is generally bad for us as humans |
|  | TAQ_Neg_5* | In my opinion humans do less things together because of technology |
| (2) Positive Attitude | TAQ_Pos_1 | In my opinion technology makes daily life easier |
|  | TAQ_Pos_2 | I think technology makes daily life more fun |
|  | TAQ_Pos_3 | In my opinion there should be even more technology around us in the future |
|  | TAQ_Pos_4 | Technology allows me to do more things on my own and without the help of others |
|  | TAQ_Pos_5 | Being surrounded by technology makes me feel more secure |
| (3) Excitement | TAQ_Exc_1 | I'm excited when I can try out new technological things |
|  | TAQ_Exc_2 | I'm often excited by some of the things that technology can do |
|  | TAQ_Exc_3 | At home, I often spend time learning more about technology and how it works |
|  | TAQ_Exc_4 | When I grow up, I want to work in something that is related to technology |
|  | TAQ_Exc_5* | I would get very bored if we went on a school trip that was only about technology |
| (4) Competency | TAQ_Comp_1 | I know more about technology than many of my classmates |
|  | TAQ_Comp_2 | It is easy for me to learn how to use a new technological thing |
|  | TAQ_Comp_3 | I'm interested in how to program computers or robots |
|  | TAQ_Comp_4 | I would feel confident to explain to someone else how a computer works |
|  | TAQ_Comp_5* | It often takes me a long time to learn how a new technological thing works |
| *Notes.* Appendix shows the child-adjusted Technological Affinity Questionnaire (TAQ), which was developed from Karrer et al.'s (2009) original questionnaire on technological affinity. The questionnaire consists of four different sub-scales: children’s (1) negative attitude towards technology, children’s (2) positive attitude towards technology, children’s (3) excitement about technology and children’s technological (4) competency. Item labels with * indicate reverse coded items. The item order was randomized for each participant in the survey. | | |

| Supplementary Material IV. Structural validity and reliability of child-adjusted TAQ |
| --- |
| The administered version of the TAQ consisted of four sub-scales (*positive attitude*, *negative attitude*, *excitement*, and *competency*) with a total number of 20 items using 5-point Likert scales (see Supplementary Material II).  Prior to the main analysis, we examined the structural validity of the child-adjusted TAQ using the Exploratory Structural Equation Modeling (ESEM) framework by Asparouhov & Muthén (2009). ESEM has been found to be a promising alternative to Confirmatory Factor Analysis (CFA) by integrating the flexibility of exploratory factor analysis (e.g., Marsh et al., 2014; Tóth-Király et al., 2017), which is especially useful when researchers have weaker hypotheses about the measurement of interest (Kline, 2016) or when cross-loadings need to be expected due to conceptually related constructs and impure nature of items in social sciences (Morin et al., 2016; Tóth-Király et al., 2017). In our case, we did have some a priori expectations for the factor structure of the child-adjusted TAQ based on Karrer et al.'s (2009) original questionnaire. These expectations were considered by specifying an ESEM-model with the oblique target rotation procedure, meaning all item loadings were freely estimated, but cross-loadings contrary to the a priori expected factor structure were ‘targeted’ to be as close to zero as possible (Asparouhov & Muthén, 2009; Browne, 2001; Marsh et al., 2014). According to the results (see Supplementary Material IV and V) the ESEM-model failed the exact fit test (χ^2^ [116] = 166.66, *p*<.01) but had a good approximate fit (RMSEA [90% CI] = .06 [.04, .07]; SRMR = .04; CFI = .97; TLI = .96) according to commonly used guidelines (see Hu & Bentler, 1999), and especially according to the standardized root mean square residual (SRMR), which is robust to the estimation method used (Shi & Maydeu-Olivares, 2019). Furthermore, less than 3% of correlation residuals (which could indicate weak local model fit, see Kline, 2016) had absolute values above .10 and were without any systematic pattern.  Following Hair et al.'s (1998) guidance for assessing the practical relevance of standardized factor loadings for sample sizes of *n*≈150, we only selected significant items with factor loadings of λ≥.45 for the main analysis. In total, two items (TAQ_Pos_5, TAQ_Comp_1) were excluded from the main analysis due to low or non-significant factor loadings. Furthermore, some of the items were more closely associated with other factors (rather than their original target factor), which is not surprising given the sub-scales of the TAQ refer to conceptually related constructs. For example, the reversely-coded items ‘*I would get very bored if we went on a school trip that was only about technology*’ (TAQ_Exc_5R) and ‘*It often takes me a long time to learn how a new technological thing works*’ (TAQ_Comp_5R) were more closely associated with the *negative attitude* factor, while ‘*I'm excited when I can try out new technological things’* (TAQ_Exc_1) and ‘*It is easy for me to learn how to use a new technological thing*’ (TAQ_Comp_2) were more closely associated with the *positive attitude* factor. In total, seven items were re-assigned before the main analysis, and, after the re-assignment, all revised sub-scales had acceptable reliability estimates of internal consistency (*positive attitude*: α=.78; *negative attitude:* α=.87; *excitement*: α=.81; *competency*: α=.73; *total TAQ:* α=.78) as indicated by the ordinal coefficient alpha (Zumbo et al., 2007; Zumbo & Kroc, 2019). |
| *Notes.* Material discusses the structural validity and reliability of child-adjusted TAQ (see also Supplementary Material III, V and VI) |

| Supplementary Material V. ESEM goodness of fit statistics and factor loadings of all TAQ items | | | | | | | | | | | | | | | | | | | | | |
| --- | --- | --- | --- | --- | --- | --- | --- | --- | --- | --- | --- | --- | --- | --- | --- | --- | --- | --- | --- | --- | --- |
| Sub-scales | Item | ESEM Solution | | | | | | | | | | | | | | | | | | | |
|  |  | Goodness of fit statistics: | | | χ^2^ (DF) = 166.66 (116), *p*<.01 | | | | RMSEA (90% CI) = .06 (.04, .07) | | | | | SRMR = .04 | | CFI = .97 | | | | TLI = .96 | |
|  |  | Factor 1 (λ): *Positive Attitude* | | | | | Factor 2 (λ): *Negative* *Attitude* | | | | | Factor 3 (λ): *Excitement* | | | | | | Factor 4 (λ): *Competency* | | | |
|  |  | λ | (SE) | *p* | | λ | | (SE) | | *p* | λ | | (SE) | | *p* | | λ | | (SE) | | *p* |
| *Positive Attitude* | TAQ_Pos_1 | **.67** | **(.16)** | **<.01** | | .01 | | (.08) | | .92 | -.10 | | (.18) | | .57 | | .23 | | (.20) | | .27 |
|  | TAQ_Pos_2 | **.34** | **(.27)** | **.21** | | .04 | | (.07) | | .56 | .51 | | (.16) | | <.01 | | .06 | | (.10) | | .54 |
|  | TAQ_Pos_3 | **.52** | **(.10)** | **<.01** | | .02 | | (.09) | | .81 | .06 | | (.13) | | .64 | | .34 | | (.11) | | <.01 |
|  | TAQ_Pos_4 | **.37** | **(.34)** | **.27** | | -.09 | | (.09) | | .29 | .60 | | (.20) | | <.01 | | -.14 | | (.10) | | .16 |
|  | TAQ_Pos_5 | **.13** | **(.10)** | **.20** | | -.17 | | (.09) | | .05 | .17 | | (.09 | | .06 | | .34 | | (.12) | | <.01 |
| *Negative Attitude* | TAQ_Neg_1* | .17 | (.09) | .07 | | **.69** | | **(.06)** | | **<.01** | -.09 | | (.13) | | .50 | | -.05 | | (.11) | | .66 |
|  | TAQ_Neg_2* | -.09 | (.13) | .50 | | **.66** | | **(.06)** | | **<.01** | .11 | | (.10) | | .29 | | -.28 | | (.09) | | <.01 |
|  | TAQ_Neg_3* | -.02 | (.10) | .84 | | **.49** | | **(.08)** | | **<.01** | .01 | | (.10) | | .91 | | -.15 | | (.12) | | .22 |
|  | TAQ_Neg_4* | .27 | (.09) | <.01 | | **.67** | | **(.07)** | | **<.01** | -.15 | | (.15) | | .33 | | -.04 | | (.13) | | .73 |
|  | TAQ_Neg_5* | -.37 | (.11) | <.01 | | **.88** | | **(.07)** | | **<.01** | .03 | | (.17) | | .84 | | .19 | | (.14) | | .17 |
| *Excitement* | TAQ_Exc_1 | .62 | (.18) | <.01 | | .01 | | (.08) | | .86 | **.31** | | **(.28)** | | **.27** | | -.05 | | (.13) | | .70 |
|  | TAQ_Exc_2 | .35 | (.18) | .05 | | .06 | | (.07) | | .39 | **.45** | | **(.16)** | | **<.05** | | .14 | | (.08) | | .10 |
|  | TAQ_Exc_3 | -.02 | (.17) | .92 | | .04 | | (.07) | | .52 | **.67** | | **(.11)** | | **<.01** | | .34 | | (.16) | | <.05 |
|  | TAQ_Exc_4 | .11 | (.11) | .29 | | -.13 | | (.07) | | .06 | **.21** | | **(.10)** | | **<.05** | | .48 | | (.08) | | <.01 |
|  | TAQ_Exc_5* | .31 | (.10) | <.01 | | .68 | | (.07) | | <.01 | **-.14** | | **(.18)** | | **.43** | | -.02 | | (.16) | | .89 |
| *Competency* | TAQ_Comp_1 | -.03 | (.11) | .77 | | .09 | | (.07) | | .22 | .39 | | (.10) | | <.01 | | **.37** | | **(.14)** | | **<.01** |
|  | TAQ_Comp_2 | .52 | (.09) | <.01 | | -.04 | | (.07) | | .62 | .07 | | (.17) | | .69 | | **.23** | | **(.17)** | | **.18** |
|  | TAQ_Comp_3 | .15 | (.18) | .42 | | .05 | | (.06) | | .41 | .08 | | (.12) | | .51 | | **.74** | | **(.11)** | | **<.01** |
|  | TAQ_Comp_4 | .11 | (.08) | .18 | | -.07 | | (.08) | | .36 | .25 | | (.09) | | <.05 | | **.45** | | **(.11)** | | **<.01** |
|  | TAQ_Comp_5* | .10 | (.10) | .32 | | .57 | | (.07) | | <.01 | .01 | | (.14) | | .92 | | **-.30** | | **(.09)** | | **<.01** |
| *Notes.* Table shows goodness of fit statistics of the ESEM (χ^2^, RMSEA, SRMR, CFA), and fully standardized factor loadings (λ), standard errors (SE) and p-values (*p*) for each TAQ-item. Items labelled with * were reverse coded before the analysis. Target factor loadings are in bold. Due to categorical response data (5-point Likert scale), a robust mean- and variance-adjusted weighted least squares estimator (WLSMV) was used. The analysis converged normally to an admissible solution. The analysis was conducted in *Mplus,* version 8.5 (Mac). Sample size *n* =143 (no missing data). | | | | | | | | | | | | | | | | | | | | | |

| Supplementary Material VI. ESEM factor variances and covariances | | | | | | | | | | | | | | | | | | | | |
| --- | --- | --- | --- | --- | --- | --- | --- | --- | --- | --- | --- | --- | --- | --- | --- | --- | --- | --- | --- | --- |
|  |  | ESEM Solution | | | | | | | | | | | | | | | | | | |
|  |  | Goodness of fit statistics: | | | χ^2^ (DF) = 166.66 (116), *p*<.01 | | | | RMSEA (90% CI) = .06 (.04, .07) | | | | | SRMR = .04 | | | CFI = .97 | | TLI = .96 | |
|  |  | Factor 1 | | | | | Factor 2 | | | | | Factor 3 | | | | | Factor 4 | | | |
|  |  | *r* | (SE) | *p* | | *r* | | (SE) | | *p* | *r* | | (SE) | | *p* | *r* | | (SE) | | *p* |
| Factor 1: *Positive Attitude* | | 1.00 | (-) | (-) | |  | |  | |  |  | |  | |  |  | |  | |  |
| Factor 2: *Negative* *Attitude* | | .20 | .06 | <.01 | | 1.00 | | (-) | | (-) |  | |  | |  |  | |  | |  |
| Factor 3: *Excitement* | | .32 | .13 | <.05 | | -.11 | | .14 | | .45 | 1.00 | | (-) | | (-) |  | |  | |  |
| Factor 4: *Competency* | | .29 | .16 | .07 | | -.28 | | .15 | | .06 | .39 | | .07 | | <.01 | 1.00 | | (-) | | (-) |
| *Notes.* Table shows goodness of fit statistics of the ESEM (χ^2^, RMSEA, SRMR, CFA), and fully standardized factor variances and covariances (*r*), standard errors (SE) and p-values (*p*). All items targeted to factor 2 (*negative attitude*) were reverse coded before the analysis (i.e., higher values refer to a weaker negative attitude towards technology). Due to categorical response data (5-point Likert scale), a robust mean- and variance-adjusted weighted least squares estimator (WLSMV) was used. The analysis converged normally to an admissible solution. The analysis was conducted in *Mplus,* version 8.5 (Mac). Sample size *n* =143 (no missing data). | | | | | | | | | | | | | | | | | | | | |

| Supplementary Material VII. SPSS syntax |
| --- |
| *Authenticity screening.  DATASET ACTIVATE DataSet1.  USE ALL.  COMPUTE filter_$=(Authenticity_Check_Strict = 1).  VARIABLE LABELS filter_$ 'Authenticity_Check_Strict = 1 (FILTER)'.  VALUE LABELS filter_$ 0 'Not Selected' 1 'Selected'.  FORMATS filter_$ (f1.0).  FILTER BY filter_$.  EXECUTE.  *Sample Descriptives after authenticity screening.  FREQUENCIES VARIABLES=Child_gender_self Child_gender_parent Child_age_parent Child_age_self  Family_relationship Parent_education Parent_age_group Additional_languages Child_language_1 Child_language_2  Child_language_3 Child_language_4 Child_language_5 Child_language_6 Child_language_7  /HISTOGRAM  /ORDER=ANALYSIS.  DESCRIPTIVES VARIABLES=Child_age_parent Child_age_self  /STATISTICS=MEAN STDDEV MIN MAX.  RECODE Child_age_parent (Lowest thru 8=0) (9 thru Highest=1) INTO Child_age_group.  VARIABLE LABELS Child_age_group 'Younger children (7 to 8 years) vs older childre (9 to eleven '+  'years)'.  EXECUTE.  *Voice Assistant Exposure Measure.  RECODE DVA_P_usage_child (4=0) (999=0) (MISSING=0) (1 thru 3=1) INTO DVA_Exposure_P_Point_1.  VARIABLE LABELS DVA_Exposure_P_Point_1 "Child has used DVA at least once in the past, parental measure".  EXECUTE.  RECODE DVA_P_usage_child (1=1) (999=0) (MISSING=0) (2 thru 4=0) INTO DVA_Exposure_P_Point_2.  VARIABLE LABELS DVA_Exposure_P_Point_2 "DVA used by child regularly, parental measure".  EXECUTE.  RECODE DVA_P_usage_home (1=1) (3=0) (999=0) (MISSING=0) INTO DVA_Exposure_P_Point_3.  VARIABLE LABELS DVA_Exposure_P_Point_3 "DVA generally used at home, parental measure".  EXECUTE.  RECODE DVA_P_device_no (1=0) (MISSING=0) (2 thru 4=1) INTO DVA_Exposure_P_Point_4.  VARIABLE LABELS DVA_Exposure_P_Point_4 "DVA used on more than one device at home, parental measure".  EXECUTE.  RECODE DVA_P_device_locate_4 (1=1) (ELSE=0) INTO DVA_Exposure_P_Point_5.  VARIABLE LABELS DVA_Exposure_P_Point_5 "DVA installed in child's room, parental measure".  EXECUTE.  RECODE DVA_P_family_friends (1=1) (2=0) (999=0) (MISSING=0) INTO DVA_Exposure_P_Point_6.  VARIABLE LABELS DVA_Exposure_P_Point_6 "Presence of DVAs in child's broader circle of family"+  "and friends, parental measure".  EXECUTE.  RECODE DVA_P_media (1=1) (2=0) (999=0) (MISSING=0) INTO DVA_Exposure_P_Point_7.  VARIABLE LABELS DVA_Exposure_P_Point_7 'Child familiar with DVAs through media, parental measure'.  EXECUTE.  RECODE DVA_C_usage_child (999=0) (MISSING=0) (4=0) (1 thru 3=1) INTO DVA_Exposure_C_Point_1.  VARIABLE LABELS DVA_Exposure_C_Point_1 'Child has used DVA at least once in the past, child measure'.  EXECUTE.  RECODE DVA_C_usage_child (1=1) (MISSING=0) (2 thru 4=0) INTO DVA_Exposure_C_Point_2.  VARIABLE LABELS DVA_Exposure_C_Point_2 'DVA used by child regularly, child measure'.  EXECUTE.  RECODE DVA_C_usage_home (1=1) (MISSING=0) (2=0) (999=0) INTO DVA_Exposure_C_Point_3.  VARIABLE LABELS DVA_Exposure_C_Point_3 'DVA generally used at home, child measure'.  EXECUTE.  RECODE DVA_C_family_friends (1=1) (MISSING=0) (2=0) (999=0) INTO DVA_Exposure_C_Point_4.  VARIABLE LABELS DVA_Exposure_C_Point_4 "Presence of DVAs in child's broader circle of family "+  "and friends, child measure".  EXECUTE.  RECODE DVA_C_media (1=1) (MISSING=0) (2=0) (999=0) INTO DVA_Exposure_C_Point_5.  VARIABLE LABELS DVA_Exposure_C_Point_5 'Child familiar with DVAs through media, child measure'.  EXECUTE.  *Voice Assistant Exposure Scores.  COMPUTE DVA_Exposure_Score_P_Total=DVA_Exposure_P_Point_1 + DVA_Exposure_P_Point_2 +  DVA_Exposure_P_Point_3 + DVA_Exposure_P_Point_4 + DVA_Exposure_P_Point_5 + DVA_Exposure_P_Point_6 +  DVA_Exposure_P_Point_7.  VARIABLE LABELS DVA_Exposure_Score_P_Total 'Sum of Points 1 to 7'.  EXECUTE.  COMPUTE DVA_Exposure_Score_P_Reduced=DVA_Exposure_P_Point_1 + DVA_Exposure_P_Point_2 +  DVA_Exposure_P_Point_3 + DVA_Exposure_P_Point_6 + DVA_Exposure_P_Point_7.  VARIABLE LABELS DVA_Exposure_Score_P_Reduced 'Sum of Points 1, 2, 3, 6, and 7'.  EXECUTE.  COMPUTE DVA_Exposure_Score_C_Total= DVA_Exposure_C_Point_1 + DVA_Exposure_C_Point_2 +  DVA_Exposure_C_Point_3 + DVA_Exposure_C_Point_4 + DVA_Exposure_C_Point_5.  VARIABLE LABELS DVA_Exposure_Score_C_Total 'Sum of Points 1 to 5'.  EXECUTE.  *Voice Assistant Exposure Groups based on Median Split (Median value of 5).  RECODE DVA_Exposure_Score_P_Total (MISSING=SYSMIS) (0 thru 5=0) (6 thru 7=1) INTO  DVA_Exposure_Group.  VARIABLE LABELS DVA_Exposure_Group 'High vs. Low DVA exposure based on median split of 5'.  EXECUTE.  *Voice Assistant Exposure Scores Analysis.  FREQUENCIES VARIABLES=DVA_Exposure_Score_P_Total DVA_Exposure_Score_C_Total  /STATISTICS=MEDIAN MODE  /HISTOGRAM  /ORDER=ANALYSIS.  CORRELATIONS  /VARIABLES=DVA_Exposure_Score_P_Reduced DVA_Exposure_Score_C_Total  /PRINT=TWOTAIL NOSIG  /STATISTICS DESCRIPTIVES  /MISSING=PAIRWISE.  NONPAR CORR  /VARIABLES=DVA_Exposure_Score_P_Reduced DVA_Exposure_Score_C_Total  /PRINT=SPEARMAN TWOTAIL NOSIG  /MISSING=PAIRWISE.  CORRELATIONS  /VARIABLES=DVA_Exposure_Score_P_Total Child_age_parent  /PRINT=TWOTAIL NOSIG  /STATISTICS DESCRIPTIVES  /MISSING=PAIRWISE.  NONPAR CORR  /VARIABLES=DVA_Exposure_Score_P_Total Child_age_parent  /PRINT=SPEARMAN TWOTAIL NOSIG  /MISSING=PAIRWISE.  SORT CASES BY Child_gender_parent.  SPLIT FILE LAYERED BY Child_gender_parent.  CORRELATIONS  /VARIABLES=DVA_Exposure_Score_P_Total Child_age_parent  /PRINT=TWOTAIL NOSIG  /STATISTICS DESCRIPTIVES  /MISSING=PAIRWISE.  NONPAR CORR  /VARIABLES=DVA_Exposure_Score_P_Total Child_age_parent  /PRINT=SPEARMAN TWOTAIL NOSIG  /MISSING=PAIRWISE.  SPLIT FILE OFF.  *Voice Assistant Exposure Groups Analysis.  CROSSTABS  /TABLES=DVA_Exposure_Group BY Child_gender_parent  /FORMAT=AVALUE TABLES  /STATISTICS=CHISQ  /CELLS=COUNT EXPECTED  /COUNT ROUND CELL.  MEANS TABLES=Child_age_parent BY Child_gender_self  /CELLS=MEAN COUNT STDDEV.  SORT CASES BY DVA_Exposure_Group.  SPLIT FILE LAYERED BY DVA_Exposure_Group.    FREQUENCIES VARIABLES=Child_gender_self Child_gender_parent Child_age_parent Child_age_self  Family_relationship Parent_education Parent_age_group Additional_languages Child_language_1 Child_language_2  Child_language_3 Child_language_4 Child_language_5 Child_language_6 Child_language_7  /HISTOGRAM  /ORDER=ANALYSIS.  DESCRIPTIVES VARIABLES=Child_age_parent  /STATISTICS=MEAN STDDEV MIN MAX.  MEANS TABLES=Child_age_parent BY Child_gender_parent  /CELLS=MEAN COUNT STDDEV.  SPLIT FILE OFF.  UNIANOVA Child_age_parent BY DVA_Exposure_Group Child_gender_parent  /CONTRAST(DVA_Exposure_Group)=Difference  /CONTRAST(Child_gender_parent)=Difference  /METHOD=SSTYPE(3)  /INTERCEPT=INCLUDE  /PLOT=PROFILE(DVA_Exposure_Group*Child_gender_parent) TYPE=LINE ERRORBAR=CI MEANREFERENCE=NO  YAXIS=AUTO  /PRINT DESCRIPTIVE HOMOGENEITY  /CRITERIA=ALPHA(.05)  /DESIGN=DVA_Exposure_Group Child_gender_parent DVA_Exposure_Group*Child_gender_parent.  *Compute TAQ scores based on adjusted structure.  COMPUTE Average_Tech_Affinity_Score=(TACQ_Neg_1 + TACQ_Neg_2 + TACQ_Neg_3 + TACQ_Neg_4 + TACQ_Neg_5 + TACQ_Pos_1 + TACQ_Pos_2 + TACQ_Pos_3 + TACQ_Pos_4  + TACQ_Exc_1 + TACQ_Exc_2 + + TACQ_Exc_3 + + TACQ_Exc_4 + + TACQ_Exc_5 + TACQ_Comp_2 + TACQ_Comp_3 + TACQ_Comp_4 + TACQ_Comp_5) / 18.  EXECUTE.  COMPUTE Average_Tech_Pos_Score=(TACQ_Pos_1 + TACQ_Pos_3 + TACQ_Exc_1 + TACQ_Comp_2) / 4.  EXECUTE.  COMPUTE Average_Tech_Neg_Score=(TACQ_Neg_1 + TACQ_Neg_2 + TACQ_Neg_3 + + TACQ_Neg_4 + TACQ_Neg_5 +  TACQ_Exc_5 + TACQ_Comp_5 ) / 7.  EXECUTE.  COMPUTE Average_Tech_Exc_Score=(TACQ_Exc_2 + TACQ_Exc_3 + TACQ_Pos_2 + TACQ_Pos_4) / 4.  EXECUTE.  COMPUTE Average_Tech_Comp_Score=(TACQ_Comp_3 + TACQ_Comp_4 + TACQ_Exc_4) / 3.  EXECUTE.  FREQUENCIES VARIABLES=Average_Tech_Affinity_Score Average_Tech_Pos_Score Average_Tech_Neg_Score Average_Tech_Exc_Score  Average_Tech_Comp_Score  /STATISTICS=STDDEV MINIMUM MAXIMUM MEAN MEDIAN  /HISTOGRAM  /ORDER=ANALYSIS.  RECODE Average_Tech_Affinity_Score (0 thru 2.29=0) (2.30 thru 4=1) INTO  Tech_Affinity_Group.  EXECUTE.  RECODE Average_Tech_Pos_Score (0 thru 3.01=0) (3.02 thru 4=1) INTO  Tech_Affinity_Group_Pos.  EXECUTE.  RECODE Average_Tech_Neg_Score (0 thru 1.44=0) (1.45 thru 4=1) INTO  Tech_Affinity_Group_Neg.  EXECUTE.  RECODE Average_Tech_Exc_Score (0 thru 3.01=0) (3.02 thru 4=1) INTO  Tech_Affinity_Group_Exc.  EXECUTE.  RECODE Average_Tech_Comp_Score (0 thru 3.01=0) (3.02 thru 4=1) INTO  Tech_Affinity_Group_Comp.  EXECUTE.  FREQUENCIES VARIABLES=Tech_Affinity_Group Tech_Affinity_Group_Pos Tech_Affinity_Group_Neg  Tech_Affinity_Group_Exc Tech_Affinity_Group_Comp  /ORDER=ANALYSIS.  CORRELATIONS  /VARIABLES=Average_Tech_Affinity_Score Average_Tech_Pos_Score Average_Tech_Neg_Score  Average_Tech_Exc_Score Average_Tech_Comp_Score DVA_Exposure_Score_P_Total  /PRINT=TWOTAIL NOSIG  /MISSING=PAIRWISE.  NONPAR CORR  /VARIABLES=Average_Tech_Affinity_Score Average_Tech_Pos_Score Average_Tech_Neg_Score  Average_Tech_Exc_Score Average_Tech_Comp_Score DVA_Exposure_Score_P_Total  /PRINT=SPEARMAN TWOTAIL NOSIG  /MISSING=PAIRWISE.  SORT CASES BY Child_gender_self.  SPLIT FILE LAYERED BY Child_gender_self.  CORRELATIONS  /VARIABLES=Average_Tech_Affinity_Score Average_Tech_Pos_Score Average_Tech_Neg_Score  Average_Tech_Exc_Score Average_Tech_Comp_Score DVA_Exposure_Score_P_Total  /PRINT=TWOTAIL NOSIG  /MISSING=PAIRWISE.  NONPAR CORR  /VARIABLES=Average_Tech_Affinity_Score Average_Tech_Pos_Score Average_Tech_Neg_Score  Average_Tech_Exc_Score Average_Tech_Comp_Score DVA_Exposure_Score_P_Total  /PRINT=SPEARMAN TWOTAIL NOSIG  /MISSING=PAIRWISE.  SPLIT FILE OFF.  MEANS TABLES=Average_Tech_Affinity_Score Average_Tech_Pos_Score Average_Tech_Neg_Score  Average_Tech_Exc_Score Average_Tech_Comp_Score BY Child_gender_self  /CELLS=MEAN COUNT STDDEV.  SORT CASES BY DVA_Exposure_Group.  SPLIT FILE LAYERED BY DVA_Exposure_Group.  MEANS TABLES=Average_Tech_Affinity_Score Average_Tech_Pos_Score Average_Tech_Neg_Score  Average_Tech_Exc_Score Average_Tech_Comp_Score BY Child_gender_self  /CELLS=MEAN COUNT STDDEV.  SPLIT FILE OFF.  T-TEST GROUPS=Child_gender_self(0 1)  /MISSING=ANALYSIS  /VARIABLES=Average_Tech_Affinity_Score Average_Tech_Pos_Score Average_Tech_Neg_Score  Average_Tech_Exc_Score Average_Tech_Comp_Score  /CRITERIA=CI(.95).  SORT CASES BY DVA_Exposure_Group.  SPLIT FILE LAYERED BY DVA_Exposure_Group.  T-TEST GROUPS=Child_gender_self(0 1)  /MISSING=ANALYSIS  /VARIABLES=Average_Tech_Affinity_Score Average_Tech_Pos_Score Average_Tech_Neg_Score  Average_Tech_Exc_Score Average_Tech_Comp_Score  /CRITERIA=CI(.95).  SPLIT FILE OFF.  SORT CASES BY Child_gender_self.  SPLIT FILE LAYERED BY Child_gender_self.  T-TEST GROUPS=DVA_Exposure_Group(0 1)  /MISSING=ANALYSIS  /VARIABLES=Average_Tech_Affinity_Score Average_Tech_Pos_Score Average_Tech_Neg_Score  Average_Tech_Exc_Score Average_Tech_Comp_Score  /CRITERIA=CI(.95).  SPLIT FILE OFF.  CORRELATIONS  /VARIABLES=Average_Tech_Affinity_Score Average_Tech_Pos_Score Average_Tech_Neg_Score  Average_Tech_Exc_Score Average_Tech_Comp_Score Child_age_self  /PRINT=TWOTAIL NOSIG  /MISSING=PAIRWISE.  NONPAR CORR  /VARIABLES=Average_Tech_Affinity_Score Average_Tech_Pos_Score Average_Tech_Neg_Score  Average_Tech_Exc_Score Average_Tech_Comp_Score Child_age_self  /PRINT=SPEARMAN TWOTAIL NOSIG  /MISSING=PAIRWISE.  T-TEST GROUPS=Child_age_group (0 1)  /MISSING=ANALYSIS  /VARIABLES=Average_Tech_Affinity_Score Average_Tech_Pos_Score Average_Tech_Neg_Score  Average_Tech_Exc_Score Average_Tech_Comp_Score  /CRITERIA=CI(.95).  SORT CASES BY DVA_Exposure_Group.  SPLIT FILE LAYERED BY DVA_Exposure_Group.  CORRELATIONS  /VARIABLES=Average_Tech_Affinity_Score Average_Tech_Pos_Score Average_Tech_Neg_Score  Average_Tech_Exc_Score Average_Tech_Comp_Score Child_age_self  /PRINT=TWOTAIL NOSIG  /MISSING=PAIRWISE.  NONPAR CORR  /VARIABLES=Average_Tech_Affinity_Score Average_Tech_Pos_Score Average_Tech_Neg_Score  Average_Tech_Exc_Score Average_Tech_Comp_Score Child_age_self  /PRINT=SPEARMAN TWOTAIL NOSIG  /MISSING=PAIRWISE.  T-TEST GROUPS=Child_age_group (0 1)  /MISSING=ANALYSIS  /VARIABLES=Average_Tech_Affinity_Score Average_Tech_Pos_Score Average_Tech_Neg_Score  Average_Tech_Exc_Score Average_Tech_Comp_Score  /CRITERIA=CI(.95).  SPLIT FILE OFF.  SORT CASES BY Child_gender_self.  SPLIT FILE LAYERED BY Child_gender_self.  CORRELATIONS  /VARIABLES=Average_Tech_Affinity_Score Average_Tech_Pos_Score Average_Tech_Neg_Score  Average_Tech_Exc_Score Average_Tech_Comp_Score Child_age_self  /PRINT=TWOTAIL NOSIG  /MISSING=PAIRWISE.  NONPAR CORR  /VARIABLES=Average_Tech_Affinity_Score Average_Tech_Pos_Score Average_Tech_Neg_Score  Average_Tech_Exc_Score Average_Tech_Comp_Score Child_age_self  /PRINT=SPEARMAN TWOTAIL NOSIG  /MISSING=PAIRWISE.  SPLIT FILE OFF.  NONPAR CORR  /VARIABLES=DVA_Exposure_Score_P_Total Average_Tech_Affinity_Score Average_Tech_Pos_Score  Average_Tech_Exc_Score Average_Tech_Neg_Score Average_Tech_Comp_Score Parent_age_group Parent_education  /PRINT=SPEARMAN TWOTAIL NOSIG  /MISSING=PAIRWISE.  SORT CASES BY Child_gender_self.  SPLIT FILE LAYERED BY Child_gender_self.  NONPAR CORR  /VARIABLES=DVA_Exposure_Score_P_Total Average_Tech_Affinity_Score Average_Tech_Pos_Score  Average_Tech_Exc_Score Average_Tech_Neg_Score Average_Tech_Comp_Score Parent_age_group Parent_education  /PRINT=SPEARMAN TWOTAIL NOSIG  /MISSING=PAIRWISE.  SPLIT FILE OFF.  SORT CASES BY Child_age_group.  SPLIT FILE LAYERED BY Child_age_group.  NONPAR CORR  /VARIABLES=DVA_Exposure_Score_P_Total Average_Tech_Affinity_Score Average_Tech_Pos_Score  Average_Tech_Exc_Score Average_Tech_Neg_Score Average_Tech_Comp_Score Parent_age_group Parent_education  /PRINT=SPEARMAN TWOTAIL NOSIG  /MISSING=PAIRWISE.  SPLIT FILE OFF.  *Ontological Categorisation Scores for Entities.  RECODE OntCat_Bio_1_1 DVA_C_media OntCat_Bio_1_3 OntCat_Bio_1_4 OntCat_Bio_1_5 OntCat_Bio_1_6  OntCat_Bio_1_2 OntCat_Bio_1_7 OntCat_Bio_1_8 OntCat_Bio_1_9 OntCat_Bio_2_1 OntCat_Bio_2_3  OntCat_Bio_2_4 OntCat_Bio_2_5 OntCat_Bio_2_6 OntCat_Bio_2_2 OntCat_Bio_2_7 OntCat_Bio_2_8  OntCat_Bio_2_9 OntCat_Bio_3_1 OntCat_Bio_3_3 OntCat_Bio_3_4 OntCat_Bio_3_5 OntCat_Bio_3_6  OntCat_Bio_3_2 OntCat_Bio_3_7 OntCat_Bio_3_8 OntCat_Bio_3_9 OntCat_Bio_4_1 OntCat_Bio_4_3  OntCat_Bio_4_4 OntCat_Bio_4_5 OntCat_Bio_4_6 OntCat_Bio_4_2 OntCat_Bio_4_7 OntCat_Bio_4_8  OntCat_Bio_4_9 OntCat_Bio_5_1 OntCat_Bio_5_3 OntCat_Bio_5_4 OntCat_Bio_5_5 OntCat_Bio_5_6  OntCat_Bio_5_2 OntCat_Bio_5_7 OntCat_Bio_5_8 OntCat_Bio_5_9 OntCat_Int_1_1 OntCat_Int_1_3  OntCat_Int_1_4 OntCat_Int_1_5 OntCat_Int_1_6 OntCat_Int_1_2 OntCat_Int_1_7 OntCat_Int_1_8  OntCat_Int_1_9 OntCat_Int_2_1 OntCat_Int_2_3 OntCat_Int_2_4 OntCat_Int_2_5 OntCat_Int_2_6  OntCat_Int_2_2 OntCat_Int_2_7 OntCat_Int_2_8 OntCat_Int_2_9 OntCat_Int_3_1 OntCat_Int_3_3  OntCat_Int_3_4 OntCat_Int_3_5 OntCat_Int_3_6 OntCat_Int_3_2 OntCat_Int_3_7 OntCat_Int_3_8  OntCat_Int_3_9 OntCat_Int_4_1 OntCat_Int_4_3 OntCat_Int_4_4 OntCat_Int_4_5 OntCat_Int_4_6  OntCat_Int_4_2 OntCat_Int_4_7 OntCat_Int_4_8 OntCat_Int_4_9 OntCat_Int_5_1 OntCat_Int_5_3  OntCat_Int_5_4 OntCat_Int_5_5 OntCat_Int_5_6 OntCat_Int_5_2 OntCat_Int_5_7 OntCat_Int_5_8  OntCat_Int_5_9 OntCat_Psy_1_1 OntCat_Psy_1_3 OntCat_Psy_1_4 OntCat_Psy_1_5 OntCat_Psy_1_6  OntCat_Psy_1_2 OntCat_Psy_1_7 OntCat_Psy_1_8 OntCat_Psy_1_9 OntCat_Psy_2_1 OntCat_Psy_2_3  OntCat_Psy_2_4 OntCat_Psy_2_5 OntCat_Psy_2_6 OntCat_Psy_2_2 OntCat_Psy_2_7 OntCat_Psy_2_8  OntCat_Psy_2_9 OntCat_Psy_3_1 OntCat_Psy_3_3 OntCat_Psy_3_4 OntCat_Psy_3_5 OntCat_Psy_3_6  OntCat_Psy_3_2 OntCat_Psy_3_7 OntCat_Psy_3_8 OntCat_Psy_3_9 OntCat_Psy_4_1 OntCat_Psy_4_3  OntCat_Psy_4_4 OntCat_Psy_4_5 OntCat_Psy_4_6 OntCat_Psy_4_2 OntCat_Psy_4_7 OntCat_Psy_4_8  OntCat_Psy_4_9 OntCat_Psy_5_1 OntCat_Psy_5_3 OntCat_Psy_5_4 OntCat_Psy_5_5 OntCat_Psy_5_6  OntCat_Psy_5_2 OntCat_Psy_5_7 OntCat_Psy_5_8 OntCat_Psy_5_9 OntCat_Art_1_1 OntCat_Art_1_3  OntCat_Art_1_4 OntCat_Art_1_5 OntCat_Art_1_6 OntCat_Art_1_2 OntCat_Art_1_7 OntCat_Art_1_8  OntCat_Art_1_9 (1=1) (ELSE=0).  EXECUTE.  COMPUTE Humans_Bio_Score=OntCat_Bio_1_1 + OntCat_Bio_2_1 + OntCat_Bio_3_1 + OntCat_Bio_4_1 +  OntCat_Bio_5_1.  VARIABLE LABELS Humans_Bio_Score 'Number of biological qualities attributed to humans'.  EXECUTE.  COMPUTE Cats_Bio_Score=OntCat_Bio_1_2 + OntCat_Bio_2_2 + OntCat_Bio_3_2 + OntCat_Bio_4_2 +  OntCat_Bio_5_2.  VARIABLE LABELS Cats_Bio_Score 'Number of biological qualities attributed to cats'.  EXECUTE.  COMPUTE Voice_Assistants_Bio_Score=OntCat_Bio_1_3 + OntCat_Bio_2_3 + OntCat_Bio_3_3 + OntCat_Bio_4_3 +  OntCat_Bio_5_3.  VARIABLE LABELS Voice_Assistants_Bio_Score 'Number of biological qualities attributed to voice assistants'.  EXECUTE.  COMPUTE Computers_Bio_Score=OntCat_Bio_1_4 + OntCat_Bio_2_4 + OntCat_Bio_3_4 + OntCat_Bio_4_4 +  OntCat_Bio_5_4.  VARIABLE LABELS Computers_Bio_Score 'Number of biological qualities attributed to computers'.  EXECUTE.  COMPUTE Plants_Bio_Score=OntCat_Bio_1_5 + OntCat_Bio_2_5 + OntCat_Bio_3_5 + OntCat_Bio_4_5 +  OntCat_Bio_5_5.  VARIABLE LABELS Plants_Bio_Score 'Number of biological qualities attributed to plants'.  EXECUTE.  COMPUTE Smartphones_Bio_Score=OntCat_Bio_1_6 + OntCat_Bio_2_6 + OntCat_Bio_3_6 + OntCat_Bio_4_6 +  OntCat_Bio_5_6.  VARIABLE LABELS Smartphones_Bio_Score 'Number of biological qualities attributed to smartphones'.  EXECUTE.  COMPUTE Dolls_Bio_Score=OntCat_Bio_1_7 + OntCat_Bio_2_7 + OntCat_Bio_3_7 + OntCat_Bio_4_7 +  OntCat_Bio_5_7.  VARIABLE LABELS Dolls_Bio_Score 'Number of biological qualities attributed to dolls'.  EXECUTE.  COMPUTE Robots_Bio_Score=OntCat_Bio_1_8 + OntCat_Bio_2_8 + OntCat_Bio_3_8 + OntCat_Bio_4_8 +  OntCat_Bio_5_8.  VARIABLE LABELS Robots_Bio_Score 'Number of biological qualities attributed to robots'.  EXECUTE.  COMPUTE Drones_Bio_Score=OntCat_Bio_1_9 + OntCat_Bio_2_9 + OntCat_Bio_3_9 + OntCat_Bio_4_9 +  OntCat_Bio_5_9.  VARIABLE LABELS Drones_Bio_Score 'Number of biological qualities attributed to drones'.  EXECUTE.  COMPUTE Humans_Int_Score=OntCat_Int_1_1 + OntCat_Int_2_1 + OntCat_Int_3_1 + OntCat_Int_4_1 +  OntCat_Int_5_1.  VARIABLE LABELS Humans_Int_Score 'Number of intelligence qualities attributed to humans'.  EXECUTE.  COMPUTE Cats_Int_Score=OntCat_Int_1_2 + OntCat_Int_2_2 + OntCat_Int_3_2 + OntCat_Int_4_2 +  OntCat_Int_5_2.  VARIABLE LABELS Cats_Int_Score 'Number of intelligence qualities attributed to cats'.  EXECUTE.  COMPUTE Voice_Assistants_Int_Score=OntCat_Int_1_3 + OntCat_Int_2_3 + OntCat_Int_3_3 + OntCat_Int_4_3 +  OntCat_Int_5_3.  VARIABLE LABELS Voice_Assistants_Int_Score 'Number of intelligence qualities attributed to voice assistants'.  EXECUTE.  COMPUTE Computers_Int_Score=OntCat_Int_1_4 + OntCat_Int_2_4 + OntCat_Int_3_4 + OntCat_Int_4_4 +  OntCat_Int_5_4.  VARIABLE LABELS Computers_Int_Score 'Number of intelligence qualities attributed to computers'.  EXECUTE.  COMPUTE Plants_Int_Score=OntCat_Int_1_5 + OntCat_Int_2_5 + OntCat_Int_3_5 + OntCat_Int_4_5 +  OntCat_Int_5_5.  VARIABLE LABELS Plants_Int_Score 'Number of biological qualities attributed to plants'.  EXECUTE.  COMPUTE Smartphones_Int_Score=OntCat_Int_1_6 + OntCat_Int_2_6 + OntCat_Int_3_6 + OntCat_Int_4_6 +  OntCat_Int_5_6.  VARIABLE LABELS Smartphones_Int_Score 'Number of intelligence qualities attributed to smartphones'.  EXECUTE.  COMPUTE Dolls_Int_Score=OntCat_Int_1_7 + OntCat_Int_2_7 + OntCat_Int_3_7 + OntCat_Int_4_7 +  OntCat_Int_5_7.  VARIABLE LABELS Dolls_Int_Score 'Number of intelligence qualities attributed to dolls'.  EXECUTE.  COMPUTE Robots_Int_Score=OntCat_Int_1_8 + OntCat_Int_2_8 + OntCat_Int_3_8 + OntCat_Int_4_8 +  OntCat_Int_5_8.  VARIABLE LABELS Robots_Int_Score 'Number of intelligence qualities attributed to robots'.  EXECUTE.  COMPUTE Drones_Int_Score=OntCat_Int_1_9 + OntCat_Int_2_9 + OntCat_Int_3_9 + OntCat_Int_4_9 +  OntCat_Int_5_9.  VARIABLE LABELS Drones_Int_Score 'Number of intelligence qualities attributed to drones'.  EXECUTE.  COMPUTE Humans_Psy_Score=OntCat_Psy_1_1 + OntCat_Psy_2_1 + OntCat_Psy_3_1 + OntCat_Psy_4_1 +  OntCat_Psy_5_1.  VARIABLE LABELS Humans_Psy_Score 'Number of psychological qualities attributed to humans'.  EXECUTE.  COMPUTE Cats_Psy_Score=OntCat_Psy_1_2 + OntCat_Psy_2_2 + OntCat_Psy_3_2 + OntCat_Psy_4_2 +  OntCat_Psy_5_2.  VARIABLE LABELS Cats_Psy_Score 'Number of psychological qualities attributed to cats'.  EXECUTE.  COMPUTE Voice_Assistants_Psy_Score=OntCat_Psy_1_3 + OntCat_Psy_2_3 + OntCat_Psy_3_3 + OntCat_Psy_4_3 +  OntCat_Psy_5_3.  VARIABLE LABELS Voice_Assistants_Psy_Score 'Number of psychological qualities attributed to voice assistants'.  EXECUTE.  COMPUTE Computers_Psy_Score=OntCat_Psy_1_4 + OntCat_Psy_2_4 + OntCat_Psy_3_4 + OntCat_Psy_4_4 +  OntCat_Psy_5_4.  VARIABLE LABELS Computers_Psy_Score 'Number of psychological qualities attributed to computers'.  EXECUTE.  COMPUTE Plants_Psy_Score=OntCat_Psy_1_5 + OntCat_Psy_2_5 + OntCat_Psy_3_5 + OntCat_Psy_4_5 +  OntCat_Psy_5_5.  VARIABLE LABELS Plants_Psy_Score 'Number of psychological qualities attributed to plants'.  EXECUTE.  COMPUTE Smartphones_Psy_Score=OntCat_Psy_1_6 + OntCat_Psy_2_6 + OntCat_Psy_3_6 + OntCat_Psy_4_6 +  OntCat_Psy_5_6.  VARIABLE LABELS Smartphones_Psy_Score 'Number of psychological qualities attributed to smartphones'.  EXECUTE.  COMPUTE Dolls_Psy_Score=OntCat_Psy_1_7 + OntCat_Psy_2_7 + OntCat_Psy_3_7 + OntCat_Psy_4_7 +  OntCat_Psy_5_7.  VARIABLE LABELS Dolls_Psy_Score 'Number of psychological qualities attributed to dolls'.  EXECUTE.  COMPUTE Robots_Psy_Score=OntCat_Psy_1_8 + OntCat_Psy_2_8 + OntCat_Psy_3_8 + OntCat_Psy_4_8 +  OntCat_Psy_5_8.  VARIABLE LABELS Robots_Psy_Score 'Number of psychological qualities attributed to robots'.  EXECUTE.  COMPUTE Drones_Psy_Score=OntCat_Psy_1_9 + OntCat_Psy_2_9 + OntCat_Psy_3_9 + OntCat_Psy_4_9 +  OntCat_Psy_5_9.  VARIABLE LABELS Drones_Psy_Score 'Number of psychological qualities attributed to drones'.  EXECUTE.  COMPUTE Average_Technological_Int_Score=(Voice_Assistants_Int_Score + Computers_Int_Score +  Smartphones_Int_Score + Robots_Int_Score + Drones_Int_Score) / 5.  VARIABLE LABELS Average_Technological_Int_Score 'Average number of intelligence-related '+  'qualities attributed to technological entities'.  EXECUTE.  DESCRIPTIVES VARIABLES=Average_Technological_Int_Score  /STATISTICS=MEAN STDDEV MIN MAX.  COMPUTE Average_Technological_Psy_Score=(Voice_Assistants_Psy_Score + Computers_Psy_Score +  Smartphones_Psy_Score + Robots_Psy_Score + Drones_Psy_Score) / 5.  VARIABLE LABELS Average_Technological_Psy_Score 'Average number of psychological '+  'qualities attributed to technological entities'.  EXECUTE.  DESCRIPTIVES VARIABLES=Average_Technological_Psy_Score  /STATISTICS=MEAN STDDEV MIN MAX.  COMPUTE Average_Technological_Bio_Score=(Voice_Assistants_Bio_Score + Computers_Bio_Score +  Smartphones_Bio_Score + Robots_Bio_Score + Drones_Bio_Score) / 5.  VARIABLE LABELS Average_Technological_Bio_Score 'Average number of biological qualities '+  'attributed to technological entities'.  EXECUTE.  DESCRIPTIVES VARIABLES=Average_Technological_Bio_Score  /STATISTICS=MEAN STDDEV MIN MAX.  COMPUTE Average_Living_Int_Score=(Humans_Int_Score + Cats_Int_Score + Plants_Int_Score) / 3.  VARIABLE LABELS Average_Living_Int_Score 'Average number of intelligence-related '+  'qualities attributed to living entities'.  EXECUTE.  DESCRIPTIVES VARIABLES=Average_Living_Int_Score  /STATISTICS=MEAN STDDEV MIN MAX.  COMPUTE Average_Living_Psy_Score=(Humans_Psy_Score + Cats_Psy_Score + Plants_Psy_Score) / 3.  VARIABLE LABELS Average_Living_Psy_Score 'Average number of psychological '+  'qualities attributed to living entities'.  EXECUTE.  DESCRIPTIVES VARIABLES=Average_Living_Psy_Score  /STATISTICS=MEAN STDDEV MIN MAX.  COMPUTE Average_Living_Bio_Score=(Humans_Bio_Score + Cats_Bio_Score + Plants_Bio_Score) / 3.  VARIABLE LABELS Average_Living_Bio_Score 'Average number of biological qualities '+  'attributed to living entities'.  EXECUTE.  DESCRIPTIVES VARIABLES=Average_Living_Bio_Score  /STATISTICS=MEAN STDDEV MIN MAX.  *Ontological Categorisation Control Zero Scores for Humans and cats.  IF (Humans_Bio_Score = 0 \| Cats_Bio_Score = 0) CONTROL_Zero_Bio_Scores_Humans_Cats=1.  VARIABLE LABELS CONTROL_Zero_Bio_Scores_Humans_Cats 'Child attributed 0 biological qualities to human and/or cat'.  EXECUTE.  RECODE CONTROL_Zero_Bio_Scores_Humans_Cats (1=1) (ELSE=0).  EXECUTE.  T-TEST GROUPS=CONTROL_Zero_Bio_Scores_Humans_Cats(0 1)  /MISSING=ANALYSIS  /VARIABLES=Duration_OntCat_Average  /CRITERIA=CI(.95).  *Freqiencies ontological qualitites.  COMPUTE Bio1_Growth_Total_Living_Entities=OntCat_Bio_1_1 + OntCat_Bio_1_2 + OntCat_Bio_1_5.  EXECUTE.  COMPUTE Bio2_Mortality_Total_Living_Entities=OntCat_Bio_2_1 + OntCat_Bio_2_2 + OntCat_Bio_2_5.  EXECUTE.  COMPUTE Bio3_Aliveness_Total_Living_Entities=OntCat_Bio_3_1 + OntCat_Bio_3_2 + OntCat_Bio_3_5.  EXECUTE.  COMPUTE Bio4_Metabolism_Total_Living_Entities=OntCat_Bio_4_1 + OntCat_Bio_4_2 + OntCat_Bio_4_5.  EXECUTE.  COMPUTE Bio5_Movement_Total_Living_Entities=OntCat_Bio_5_1 + OntCat_Bio_5_2 + OntCat_Bio_5_5.  EXECUTE.  COMPUTE Int1_Intellegence_Total_Living_Entities=OntCat_Int_1_1 + OntCat_Int_1_2 + OntCat_Int_1_5.  EXECUTE.  COMPUTE Int2_Learning_Total_Living_Entities=OntCat_Int_2_1 + OntCat_Int_2_2 + OntCat_Int_2_5.  EXECUTE.  COMPUTE Int3_Planning_Total_Living_Entities=OntCat_Int_3_1 + OntCat_Int_3_2 + OntCat_Int_3_5.  EXECUTE.  COMPUTE Int4_Remembering_Total_Living_Entities=OntCat_Int_4_1 + OntCat_Int_4_2 + OntCat_Int_4_5.  EXECUTE.  COMPUTE Int5_Calculation_Total_Living_Entities=OntCat_Int_5_1 + OntCat_Int_5_2 + OntCat_Int_5_5.  EXECUTE.  COMPUTE Psy1_Emotionality_Total_Living_Entities=OntCat_Psy_1_1 + OntCat_Psy_1_2 + OntCat_Psy_1_5.  EXECUTE.  COMPUTE Psy2_Empathy_Total_Living_Entities=OntCat_Psy_2_1 + OntCat_Psy_2_2 + OntCat_Psy_2_5.  EXECUTE.  COMPUTE Psy3_Volition_Total_Living_Entities=OntCat_Psy_3_1 + OntCat_Psy_3_2 + OntCat_Psy_3_5.  EXECUTE.  COMPUTE Psy4_MoralValue_Total_Living_Entities=OntCat_Psy_4_1 + OntCat_Psy_4_2 + OntCat_Psy_4_5.  EXECUTE.  COMPUTE Psy5_MoralConsc_Total_Living_Entities=OntCat_Psy_5_1 + OntCat_Psy_5_2 + OntCat_Psy_5_5.  EXECUTE.  COMPUTE Bio1_Growth_Total_Technological_Entities=OntCat_Bio_1_3 + OntCat_Bio_1_4 + OntCat_Bio_1_6 + OntCat_Bio_1_8 + OntCat_Bio_1_9.  EXECUTE.  COMPUTE Bio2_Mortality_Total_Technological_Entities=OntCat_Bio_2_3 + OntCat_Bio_2_4 + OntCat_Bio_2_6 + OntCat_Bio_2_8 + OntCat_Bio_2_9.  EXECUTE.  COMPUTE Bio3_Aliveness_Total_Technological_Entities=OntCat_Bio_3_3 + OntCat_Bio_3_4 + OntCat_Bio_3_6 + OntCat_Bio_3_8 + OntCat_Bio_3_9.  EXECUTE.  COMPUTE Bio4_Metabolism_Total_Technological_Entities=OntCat_Bio_4_3 + OntCat_Bio_4_4 + OntCat_Bio_4_6 + OntCat_Bio_4_8 + OntCat_Bio_4_9.  EXECUTE.  COMPUTE Bio5_Movement_Total_Technological_Entities=OntCat_Bio_5_3 + OntCat_Bio_5_4 + OntCat_Bio_5_6 + OntCat_Bio_5_8 + OntCat_Bio_5_9.  EXECUTE.  COMPUTE Int1_Intellegence_Total_Technological_Entities=OntCat_Int_1_3 + OntCat_Int_1_4 + OntCat_Int_1_6 + OntCat_Int_1_8 + OntCat_Int_1_9.  EXECUTE.  COMPUTE Int2_Learning_Total_Technological_Entities=OntCat_Int_2_3 + OntCat_Int_2_4 + OntCat_Int_2_6 + OntCat_Int_2_8 + OntCat_Int_2_9.  EXECUTE.  COMPUTE Int3_Planning_Total_Technological_Entities=OntCat_Int_3_3 + OntCat_Int_3_4 + OntCat_Int_3_6 + OntCat_Int_3_8 + OntCat_Int_3_9.  EXECUTE.  COMPUTE Int4_Remembering_Technological_Entities=OntCat_Int_4_3 + OntCat_Int_4_4 + OntCat_Int_4_6 + OntCat_Int_4_8 + OntCat_Int_4_9.  EXECUTE.  COMPUTE Int5_Calculation_Technological_Entities=OntCat_Int_5_3 + OntCat_Int_5_4 + OntCat_Int_5_6 + OntCat_Int_5_8 + OntCat_Int_5_9.  EXECUTE.  COMPUTE Psy1_Emotionality_Technological_Entities=OntCat_Psy_1_3 + OntCat_Psy_1_4 + OntCat_Psy_1_6 + OntCat_Psy_1_8 + OntCat_Psy_1_9.  EXECUTE.  COMPUTE Psy2_Empathy_Total_Technological_Entities=OntCat_Psy_2_3 + OntCat_Psy_2_4 + OntCat_Psy_2_6 + OntCat_Psy_2_8 + OntCat_Psy_2_9.  EXECUTE.  COMPUTE Psy3_Volition_Total_Technological_Entities=OntCat_Psy_3_3 + OntCat_Psy_3_4 + OntCat_Psy_3_6 + OntCat_Psy_3_8 + OntCat_Psy_3_9.  EXECUTE.  COMPUTE Psy4_MoralValue_Total_Technological_Entities=OntCat_Psy_4_3 + OntCat_Psy_4_4 + OntCat_Psy_4_6 + OntCat_Psy_4_8 + OntCat_Psy_4_9.  EXECUTE.  COMPUTE Psy5_MoralConsc_Total_Technological_Entities=OntCat_Psy_5_3 + OntCat_Psy_5_4 + OntCat_Psy_5_6 + OntCat_Psy_5_8 + OntCat_Psy_5_9.  EXECUTE.  FREQUENCIES VARIABLES=Bio1_Growth_Total_Living_Entities Bio2_Mortality_Total_Living_Entities  Bio3_Aliveness_Total_Living_Entities Bio4_Metabolism_Total_Living_Entities  Bio5_Movement_Total_Living_Entities Int1_Intellegence_Total_Living_Entities  Int2_Learning_Total_Living_Entities Int3_Planning_Total_Living_Entities  Int4_Remembering_Total_Living_Entities Int5_Calculation_Total_Living_Entities  Psy1_Emotionality_Total_Living_Entities Psy2_Empathy_Total_Living_Entities  Psy3_Volition_Total_Living_Entities Psy4_MoralValue_Total_Living_Entities  Psy5_MoralConsc_Total_Living_Entities Bio1_Growth_Total_Technological_Entities  Bio2_Mortality_Total_Technological_Entities Bio3_Aliveness_Total_Technological_Entities  Bio4_Metabolism_Total_Technological_Entities Bio5_Movement_Total_Technological_Entities  Int1_Intellegence_Total_Technological_Entities Int2_Learning_Total_Technological_Entities  Int3_Planning_Total_Technological_Entities Int4_Remembering_Technological_Entities  Int5_Calculation_Technological_Entities Psy1_Emotionality_Technological_Entities  Psy2_Empathy_Total_Technological_Entities Psy3_Volition_Total_Technological_Entities  Psy4_MoralValue_Total_Technological_Entities Psy5_MoralConsc_Total_Technological_Entities  /STATISTICS=STDDEV MINIMUM MAXIMUM MEAN MEDIAN  /HISTOGRAM  /ORDER=ANALYSIS.  *General ontological conceptualization patterns.  FREQUENCIES VARIABLES= Humans_Bio_Score Humans_Int_Score Humans_Psy_Score Cats_Bio_Score  Cats_Int_Score Cats_Psy_Score Plants_Bio_Score Plants_Int_Score Plants_Psy_Score Voice_Assistants_Bio_Score  Voice_Assistants_Int_Score Voice_Assistants_Psy_Score Smartphones_Bio_Score Smartphones_Int_Score Smartphones_Psy_Score  Robots_Bio_Score Robots_Int_Score Robots_Psy_Score Computers_Bio_Score Computers_Int_Score Computers_Psy_Score  Drones_Bio_Score Drones_Int_Score Drones_Psy_Score Dolls_Bio_Score Dolls_Int_Score Dolls_Psy_Score  /STATISTICS=MEAN STDDEV  /HISTOGRAM  /ORDER=ANALYSIS.  SORT CASES BY DVA_Exposure_Group.  SPLIT FILE LAYERED BY DVA_Exposure_Group.  FREQUENCIES VARIABLES= Humans_Bio_Score Humans_Int_Score Humans_Psy_Score Cats_Bio_Score  Cats_Int_Score Cats_Psy_Score Plants_Bio_Score Plants_Int_Score Plants_Psy_Score Voice_Assistants_Bio_Score  Voice_Assistants_Int_Score Voice_Assistants_Psy_Score Smartphones_Bio_Score Smartphones_Int_Score Smartphones_Psy_Score  Robots_Bio_Score Robots_Int_Score Robots_Psy_Score Computers_Bio_Score Computers_Int_Score Computers_Psy_Score  Drones_Bio_Score Drones_Int_Score Drones_Psy_Score Dolls_Bio_Score Dolls_Int_Score Dolls_Psy_Score  /STATISTICS=MEAN STDDEV  /HISTOGRAM  /ORDER=ANALYSIS.  SPLIT FILE OFF.  SORT CASES BY Child_age_group.  SPLIT FILE LAYERED BY Child_age_group.  FREQUENCIES VARIABLES= Humans_Bio_Score Humans_Int_Score Humans_Psy_Score Cats_Bio_Score  Cats_Int_Score Cats_Psy_Score Plants_Bio_Score Plants_Int_Score Plants_Psy_Score Voice_Assistants_Bio_Score  Voice_Assistants_Int_Score Voice_Assistants_Psy_Score Smartphones_Bio_Score Smartphones_Int_Score Smartphones_Psy_Score  Robots_Bio_Score Robots_Int_Score Robots_Psy_Score Computers_Bio_Score Computers_Int_Score Computers_Psy_Score  Drones_Bio_Score Drones_Int_Score Drones_Psy_Score Dolls_Bio_Score Dolls_Int_Score Dolls_Psy_Score  /STATISTICS=MEAN STDDEV  /HISTOGRAM  /ORDER=ANALYSIS.  SPLIT FILE OFF.  FREQUENCIES VARIABLES=OntCat_Bio_1_1 OntCat_Bio_2_1 OntCat_Bio_3_1 OntCat_Bio_4_1 OntCat_Bio_5_1  /HISTOGRAM  /ORDER=ANALYSIS.  FREQUENCIES VARIABLES=OntCat_Bio_1_2 OntCat_Bio_2_2 OntCat_Bio_3_2 OntCat_Bio_4_2 OntCat_Bio_5_2  /HISTOGRAM  /ORDER=ANALYSIS.  FREQUENCIES VARIABLES=OntCat_Bio_1_8 OntCat_Bio_2_8 OntCat_Bio_3_8 OntCat_Bio_4_8 OntCat_Bio_5_8  /HISTOGRAM  /ORDER=ANALYSIS.  FREQUENCIES VARIABLES=OntCat_Bio_1_9 OntCat_Bio_2_9 OntCat_Bio_3_9 OntCat_Bio_4_9 OntCat_Bio_5_9  /HISTOGRAM  /ORDER=ANALYSIS.  FREQUENCIES VARIABLES=OntCat_Int_1_6 OntCat_Int_2_6 OntCat_Int_3_6 OntCat_Int_4_6 OntCat_Int_5_6  /ORDER=ANALYSIS.  FREQUENCIES VARIABLES=OntCat_Psy_1_8 OntCat_Psy_2_8 OntCat_Psy_3_8 OntCat_Psy_4_8 OntCat_Psy_5_8  /ORDER=ANALYSIS.  CORRELATIONS  /VARIABLES=Humans_Bio_Score Humans_Int_Score Humans_Psy_Score  /PRINT=TWOTAIL NOSIG  /MISSING=PAIRWISE.  CORRELATIONS  /VARIABLES=Cats_Bio_Score Cats_Int_Score Cats_Psy_Score  /PRINT=TWOTAIL NOSIG  /MISSING=PAIRWISE.  CORRELATIONS  /VARIABLES=Plants_Bio_Score Plants_Int_Score Plants_Psy_Score  /PRINT=TWOTAIL NOSIG  /MISSING=PAIRWISE.  CORRELATIONS  /VARIABLES=Voice_Assistants_Bio_Score Voice_Assistants_Int_Score Voice_Assistants_Psy_Score  /PRINT=TWOTAIL NOSIG  /MISSING=PAIRWISE.  CORRELATIONS  /VARIABLES=Smartphones_Bio_Score Smartphones_Int_Score Smartphones_Psy_Score  /PRINT=TWOTAIL NOSIG  /MISSING=PAIRWISE.  CORRELATIONS  /VARIABLES=Robots_Bio_Score Robots_Int_Score Robots_Psy_Score  /PRINT=TWOTAIL NOSIG  /MISSING=PAIRWISE.  CORRELATIONS  /VARIABLES=Computers_Bio_Score Computers_Int_Score Computers_Psy_Score  /PRINT=TWOTAIL NOSIG  /MISSING=PAIRWISE.  CORRELATIONS  /VARIABLES=Drones_Bio_Score Drones_Int_Score Drones_Psy_Score  /PRINT=TWOTAIL NOSIG  /MISSING=PAIRWISE.  CORRELATIONS  /VARIABLES=Dolls_Bio_Score Dolls_Int_Score Dolls_Psy_Score  /PRINT=TWOTAIL NOSIG  /MISSING=PAIRWISE.  GLM Humans_Bio_Score Humans_Int_Score Humans_Psy_Score  /WSFACTOR=Ontological_scores_humans 3 Simple  /METHOD=SSTYPE(3)  /PLOT=PROFILE(Ontological_scores_humans) TYPE=LINE ERRORBAR=CI  MEANREFERENCE=NO YAXIS=AUTO  /CRITERIA=ALPHA(.05)  /WSDESIGN=Ontological_scores_humans.  GLM Cats_Bio_Score Cats_Int_Score Cats_Psy_Score  /WSFACTOR=Ontological_scores_cats 3 Simple  /METHOD=SSTYPE(3)  /PLOT=PROFILE(Ontological_scores_cats) TYPE=LINE ERRORBAR=CI  MEANREFERENCE=NO YAXIS=AUTO  /CRITERIA=ALPHA(.05)  /WSDESIGN=Ontological_scores_cats.  GLM Plants_Bio_Score Plants_Int_Score Plants_Psy_Score  /WSFACTOR=Ontological_scores_plants 3 Simple  /METHOD=SSTYPE(3)  /PLOT=PROFILE(Ontological_scores_plants) TYPE=LINE ERRORBAR=CI  MEANREFERENCE=NO YAXIS=AUTO  /CRITERIA=ALPHA(.05)  /WSDESIGN=Ontological_scores_plants.  GLM Voice_Assistants_Bio_Score Voice_Assistants_Int_Score Voice_Assistants_Psy_Score  /WSFACTOR=Ontological_scores_voice_assistants 3 Simple  /METHOD=SSTYPE(3)  /PLOT=PROFILE(Ontological_scores_voice_assistants) TYPE=LINE ERRORBAR=CI  MEANREFERENCE=NO YAXIS=AUTO  /CRITERIA=ALPHA(.05)  /WSDESIGN=Ontological_scores_voice_assistants.  GLM Smartphones_Bio_Score Smartphones_Int_Score Smartphones_Psy_Score  /WSFACTOR=Ontological_scores_smartphones 3 Simple  /METHOD=SSTYPE(3)  /PLOT=PROFILE(Ontological_scores_smartphones) TYPE=LINE ERRORBAR=CI  MEANREFERENCE=NO YAXIS=AUTO  /CRITERIA=ALPHA(.05)  /WSDESIGN=Ontological_scores_smartphones.  GLM Robots_Bio_Score Robots_Int_Score Robots_Psy_Score  /WSFACTOR=Ontological_scores_humanoid_robots 3 Simple  /METHOD=SSTYPE(3)  /PLOT=PROFILE(Ontological_scores_humanoid_robots) TYPE=LINE ERRORBAR=CI  MEANREFERENCE=NO YAXIS=AUTO  /CRITERIA=ALPHA(.05)  /WSDESIGN=Ontological_scores_humanoid_robots.  GLM Computers_Bio_Score Computers_Int_Score Computers_Psy_Score  /WSFACTOR=Ontological_scores_computers 3 Simple  /METHOD=SSTYPE(3)  /PLOT=PROFILE(Ontological_scores_computers) TYPE=LINE ERRORBAR=CI  MEANREFERENCE=NO YAXIS=AUTO  /CRITERIA=ALPHA(.05)  /WSDESIGN=Ontological_scores_computers.  GLM Drones_Bio_Score Drones_Int_Score Drones_Psy_Score  /WSFACTOR=Ontological_scores_drones 3 Simple  /METHOD=SSTYPE(3)  /PLOT=PROFILE(Ontological_scores_drones) TYPE=LINE ERRORBAR=CI  MEANREFERENCE=NO YAXIS=AUTO  /CRITERIA=ALPHA(.05)  /WSDESIGN=Ontological_scores_drones.  GLM Dolls_Bio_Score Dolls_Int_Score Dolls_Psy_Score  /WSFACTOR=Ontological_scores_dolls 3 Simple  /METHOD=SSTYPE(3)  /PLOT=PROFILE(Ontological_scores_dolls) TYPE=LINE ERRORBAR=CI  MEANREFERENCE=NO YAXIS=AUTO  /CRITERIA=ALPHA(.05)  /WSDESIGN=Ontological_scores_dolls.  GLM Humans_Bio_Score Humans_Int_Score Humans_Psy_Score  /WSFACTOR=Ontological_scores_humans 3 Repeated  /METHOD=SSTYPE(3)  /PLOT=PROFILE(Ontological_scores_humans) TYPE=LINE ERRORBAR=CI  MEANREFERENCE=NO YAXIS=AUTO  /CRITERIA=ALPHA(.05)  /WSDESIGN=Ontological_scores_humans.  GLM Cats_Bio_Score Cats_Int_Score Cats_Psy_Score  /WSFACTOR=Ontological_scores_cats 3 Repeated  /METHOD=SSTYPE(3)  /PLOT=PROFILE(Ontological_scores_cats) TYPE=LINE ERRORBAR=CI  MEANREFERENCE=NO YAXIS=AUTO  /CRITERIA=ALPHA(.05)  /WSDESIGN=Ontological_scores_cats.  GLM Plants_Bio_Score Plants_Int_Score Plants_Psy_Score  /WSFACTOR=Ontological_scores_plants 3 Repeated  /METHOD=SSTYPE(3)  /PLOT=PROFILE(Ontological_scores_plants) TYPE=LINE ERRORBAR=CI  MEANREFERENCE=NO YAXIS=AUTO  /CRITERIA=ALPHA(.05)  /WSDESIGN=Ontological_scores_plants.  GLM Voice_Assistants_Bio_Score Voice_Assistants_Int_Score Voice_Assistants_Psy_Score  /WSFACTOR=Ontological_scores_voice_assistants 3 Repeated  /METHOD=SSTYPE(3)  /PLOT=PROFILE(Ontological_scores_voice_assistants) TYPE=LINE ERRORBAR=CI  MEANREFERENCE=NO YAXIS=AUTO  /CRITERIA=ALPHA(.05)  /WSDESIGN=Ontological_scores_voice_assistants.  GLM Smartphones_Bio_Score Smartphones_Int_Score Smartphones_Psy_Score  /WSFACTOR=Ontological_scores_smartphones 3 Repeated  /METHOD=SSTYPE(3)  /PLOT=PROFILE(Ontological_scores_smartphones) TYPE=LINE ERRORBAR=CI  MEANREFERENCE=NO YAXIS=AUTO  /CRITERIA=ALPHA(.05)  /WSDESIGN=Ontological_scores_smartphones.  GLM Robots_Bio_Score Robots_Int_Score Robots_Psy_Score  /WSFACTOR=Ontological_scores_humanoid_robots 3 Repeated  /METHOD=SSTYPE(3)  /PLOT=PROFILE(Ontological_scores_humanoid_robots) TYPE=LINE ERRORBAR=CI  MEANREFERENCE=NO YAXIS=AUTO  /CRITERIA=ALPHA(.05)  /WSDESIGN=Ontological_scores_humanoid_robots.  GLM Computers_Bio_Score Computers_Int_Score Computers_Psy_Score  /WSFACTOR=Ontological_scores_computers 3 Repeated  /METHOD=SSTYPE(3)  /PLOT=PROFILE(Ontological_scores_computers) TYPE=LINE ERRORBAR=CI  MEANREFERENCE=NO YAXIS=AUTO  /CRITERIA=ALPHA(.05)  /WSDESIGN=Ontological_scores_computers.  GLM Drones_Bio_Score Drones_Int_Score Drones_Psy_Score  /WSFACTOR=Ontological_scores_drones 3 Repeated  /METHOD=SSTYPE(3)  /PLOT=PROFILE(Ontological_scores_drones) TYPE=LINE ERRORBAR=CI  MEANREFERENCE=NO YAXIS=AUTO  /CRITERIA=ALPHA(.05)  /WSDESIGN=Ontological_scores_drones.  GLM Dolls_Bio_Score Dolls_Int_Score Dolls_Psy_Score  /WSFACTOR=Ontological_scores_dolls 3 Repeated  /METHOD=SSTYPE(3)  /PLOT=PROFILE(Ontological_scores_dolls) TYPE=LINE ERRORBAR=CI  MEANREFERENCE=NO YAXIS=AUTO  /CRITERIA=ALPHA(.05)  /WSDESIGN=Ontological_scores_dolls.  GLM Humans_Bio_Score Humans_Int_Score Humans_Psy_Score BY Child_age_group  /WSFACTOR=Ontological_scores_humans 3 Difference  /METHOD=SSTYPE(3)  /PLOT=PROFILE(Ontological_scores_humans*Child_age_group) TYPE=LINE ERRORBAR=CI  MEANREFERENCE=NO YAXIS=AUTO  /CRITERIA=ALPHA(.05)  /WSDESIGN=Ontological_scores_humans  /DESIGN=Child_age_group.  GLM Cats_Bio_Score Cats_Int_Score Cats_Psy_Score BY Child_age_group  /WSFACTOR=Ontological_scores_cats 3 Difference  /METHOD=SSTYPE(3)  /PLOT=PROFILE(Ontological_scores_cats*Child_age_group) TYPE=LINE ERRORBAR=CI  MEANREFERENCE=NO YAXIS=AUTO  /CRITERIA=ALPHA(.05)  /WSDESIGN=Ontological_scores_cats  /DESIGN=Child_age_group.  GLM Plants_Bio_Score Plants_Int_Score Plants_Psy_Score BY Child_age_group  /WSFACTOR=Ontological_scores_plants 3 Difference  /METHOD=SSTYPE(3)  /PLOT=PROFILE(Ontological_scores_plants*Child_age_group) TYPE=LINE ERRORBAR=CI  MEANREFERENCE=NO YAXIS=AUTO  /CRITERIA=ALPHA(.05)  /WSDESIGN=Ontological_scores_plants  /DESIGN=Child_age_group.  GLM Voice_Assistants_Bio_Score Voice_Assistants_Int_Score Voice_Assistants_Psy_Score BY Child_age_group  /WSFACTOR=Ontological_scores_voice_assistants 3 Difference  /METHOD=SSTYPE(3)  /PLOT=PROFILE(Ontological_scores_voice_assistants*Child_age_group) TYPE=LINE ERRORBAR=CI  MEANREFERENCE=NO YAXIS=AUTO  /CRITERIA=ALPHA(.05)  /WSDESIGN=Ontological_scores_voice_assistants  /DESIGN=Child_age_group.  GLM Smartphones_Bio_Score Smartphones_Int_Score Smartphones_Psy_Score BY Child_age_group  /WSFACTOR=Ontological_scores_smartphones 3 Difference  /METHOD=SSTYPE(3)  /PLOT=PROFILE(Ontological_scores_smartphones*Child_age_group) TYPE=LINE ERRORBAR=CI  MEANREFERENCE=NO YAXIS=AUTO  /CRITERIA=ALPHA(.05)  /WSDESIGN=Ontological_scores_smartphones  /DESIGN=Child_age_group.  GLM Robots_Bio_Score Robots_Int_Score Robots_Psy_Score BY Child_age_group  /WSFACTOR=Ontological_scores_humanoid_robots 3 Difference  /METHOD=SSTYPE(3)  /PLOT=PROFILE(Ontological_scores_humanoid_robots*Child_age_group) TYPE=LINE ERRORBAR=CI  MEANREFERENCE=NO YAXIS=AUTO  /CRITERIA=ALPHA(.05)  /WSDESIGN=Ontological_scores_humanoid_robots  /DESIGN=Child_age_group.  GLM Computers_Bio_Score Computers_Int_Score Computers_Psy_Score BY Child_age_group  /WSFACTOR=Ontological_scores_computers 3 Difference  /METHOD=SSTYPE(3)  /PLOT=PROFILE(Ontological_scores_computers*Child_age_group) TYPE=LINE ERRORBAR=CI  MEANREFERENCE=NO YAXIS=AUTO  /CRITERIA=ALPHA(.05)  /WSDESIGN=Ontological_scores_computers  /DESIGN=Child_age_group.  GLM Drones_Bio_Score Drones_Int_Score Drones_Psy_Score BY Child_age_group  /WSFACTOR=Ontological_scores_drones 3 Difference  /METHOD=SSTYPE(3)  /PLOT=PROFILE(Ontological_scores_drones*Child_age_group) TYPE=LINE ERRORBAR=CI  MEANREFERENCE=NO YAXIS=AUTO  /CRITERIA=ALPHA(.05)  /WSDESIGN=Ontological_scores_drones  /DESIGN=Child_age_group.  GLM Dolls_Bio_Score Dolls_Int_Score Dolls_Psy_Score BY Child_age_group  /WSFACTOR=Ontological_scores_dolls 3 Difference  /METHOD=SSTYPE(3)  /PLOT=PROFILE(Ontological_scores_dolls*Child_age_group) TYPE=LINE ERRORBAR=CI  MEANREFERENCE=NO YAXIS=AUTO  /CRITERIA=ALPHA(.05)  /WSDESIGN=Ontological_scores_dolls  /DESIGN=Child_age_group.  GLM Humans_Bio_Score Humans_Int_Score Humans_Psy_Score BY Child_gender_self  /WSFACTOR=Ontological_scores_humans 3 Difference  /METHOD=SSTYPE(3)  /PLOT=PROFILE(Ontological_scores_humans*Child_gender_self) TYPE=LINE ERRORBAR=CI  MEANREFERENCE=NO YAXIS=AUTO  /CRITERIA=ALPHA(.05)  /WSDESIGN=Ontological_scores_humans  /DESIGN=Child_gender_self.  GLM Cats_Bio_Score Cats_Int_Score Cats_Psy_Score BY Child_gender_self  /WSFACTOR=Ontological_scores_cats 3 Difference  /METHOD=SSTYPE(3)  /PLOT=PROFILE(Ontological_scores_cats*Child_gender_self) TYPE=LINE ERRORBAR=CI  MEANREFERENCE=NO YAXIS=AUTO  /CRITERIA=ALPHA(.05)  /WSDESIGN=Ontological_scores_cats  /DESIGN=Child_gender_self.  GLM Plants_Bio_Score Plants_Int_Score Plants_Psy_Score BY Child_gender_self  /WSFACTOR=Ontological_scores_plants 3 Difference  /METHOD=SSTYPE(3)  /PLOT=PROFILE(Ontological_scores_plants*Child_gender_self) TYPE=LINE ERRORBAR=CI  MEANREFERENCE=NO YAXIS=AUTO  /CRITERIA=ALPHA(.05)  /WSDESIGN=Ontological_scores_plants  /DESIGN=Child_gender_self.  GLM Voice_Assistants_Bio_Score Voice_Assistants_Int_Score Voice_Assistants_Psy_Score BY Child_gender_self  /WSFACTOR=Ontological_scores_voice_assistants 3 Difference  /METHOD=SSTYPE(3)  /PLOT=PROFILE(Ontological_scores_voice_assistants*Child_gender_self) TYPE=LINE ERRORBAR=CI  MEANREFERENCE=NO YAXIS=AUTO  /CRITERIA=ALPHA(.05)  /WSDESIGN=Ontological_scores_voice_assistants  /DESIGN=Child_gender_self.  GLM Smartphones_Bio_Score Smartphones_Int_Score Smartphones_Psy_Score BY Child_gender_self  /WSFACTOR=Ontological_scores_smartphones 3 Difference  /METHOD=SSTYPE(3)  /PLOT=PROFILE(Ontological_scores_smartphones*Child_gender_self) TYPE=LINE ERRORBAR=CI  MEANREFERENCE=NO YAXIS=AUTO  /CRITERIA=ALPHA(.05)  /WSDESIGN=Ontological_scores_smartphones  /DESIGN=Child_gender_self.  GLM Robots_Bio_Score Robots_Int_Score Robots_Psy_Score BY Child_gender_self  /WSFACTOR=Ontological_scores_humanoid_robots 3 Difference  /METHOD=SSTYPE(3)  /PLOT=PROFILE(Ontological_scores_humanoid_robots*Child_gender_self) TYPE=LINE ERRORBAR=CI  MEANREFERENCE=NO YAXIS=AUTO  /CRITERIA=ALPHA(.05)  /WSDESIGN=Ontological_scores_humanoid_robots  /DESIGN=Child_gender_self.  GLM Computers_Bio_Score Computers_Int_Score Computers_Psy_Score BY Child_gender_self  /WSFACTOR=Ontological_scores_computers 3 Difference  /METHOD=SSTYPE(3)  /PLOT=PROFILE(Ontological_scores_computers*Child_gender_self) TYPE=LINE ERRORBAR=CI  MEANREFERENCE=NO YAXIS=AUTO  /CRITERIA=ALPHA(.05)  /WSDESIGN=Ontological_scores_computers  /DESIGN=Child_gender_self.  GLM Drones_Bio_Score Drones_Int_Score Drones_Psy_Score BY Child_gender_self  /WSFACTOR=Ontological_scores_drones 3 Difference  /METHOD=SSTYPE(3)  /PLOT=PROFILE(Ontological_scores_drones*Child_gender_self) TYPE=LINE ERRORBAR=CI  MEANREFERENCE=NO YAXIS=AUTO  /CRITERIA=ALPHA(.05)  /WSDESIGN=Ontological_scores_drones  /DESIGN=Child_gender_self.  GLM Dolls_Bio_Score Dolls_Int_Score Dolls_Psy_Score BY Child_gender_self  /WSFACTOR=Ontological_scores_dolls 3 Difference  /METHOD=SSTYPE(3)  /PLOT=PROFILE(Ontological_scores_dolls*Child_gender_self) TYPE=LINE ERRORBAR=CI  MEANREFERENCE=NO YAXIS=AUTO  /CRITERIA=ALPHA(.05)  /WSDESIGN=Ontological_scores_dolls  /DESIGN=Child_gender_self.  T-TEST PAIRS=Robots_Bio_Score Robots_Int_Score Robots_Psy_Score WITH Drones_Bio_Score  Computers_Int_Score Voice_Assistants_Psy_Score (PAIRED)  /CRITERIA=CI(.9500)  /MISSING=ANALYSIS.  SORT CASES BY Child_age_group.  SPLIT FILE LAYERED BY Child_age_group.  T-TEST PAIRS=Robots_Bio_Score Robots_Int_Score Robots_Psy_Score WITH Drones_Bio_Score  Computers_Int_Score Voice_Assistants_Psy_Score (PAIRED)  /CRITERIA=CI(.9500)  /MISSING=ANALYSIS.  SPLIT FILE off.  CORRELATIONS  /VARIABLES=Voice_Assistants_Bio_Score Voice_Assistants_Int_Score Voice_Assistants_Psy_Score  Computers_Bio_Score Computers_Int_Score Computers_Psy_Score Smartphones_Bio_Score  Smartphones_Int_Score Smartphones_Psy_Score Robots_Bio_Score Robots_Int_Score Robots_Psy_Score  Drones_Bio_Score Drones_Int_Score Drones_Psy_Score Humans_Bio_Score Humans_Int_Score Humans_Psy_Score  Cats_Bio_Score Cats_Int_Score Cats_Psy_Score Plants_Bio_Score Plants_Int_Score Plants_Psy_Score Dolls_Bio_Score  Dolls_Int_Score Dolls_Psy_Score Humans_Bio_Score Humans_Int_Score Humans_Psy_Score Cats_Bio_Score Cats_Int_Score  Cats_Psy_Score Plants_Bio_Score Plants_Int_Score Plants_Psy_Score Dolls_Bio_Score Dolls_Int_Score Dolls_Psy_Score  Child_age_self  /PRINT=TWOTAIL NOSIG  /MISSING=PAIRWISE.  NONPAR CORR  /VARIABLES=Voice_Assistants_Bio_Score Voice_Assistants_Int_Score Voice_Assistants_Psy_Score  Computers_Bio_Score Computers_Int_Score Computers_Psy_Score Smartphones_Bio_Score  Smartphones_Int_Score Smartphones_Psy_Score Robots_Bio_Score Robots_Int_Score Robots_Psy_Score  Drones_Bio_Score Drones_Int_Score Drones_Psy_Score Humans_Bio_Score Humans_Int_Score Humans_Psy_Score  Cats_Bio_Score Cats_Int_Score Cats_Psy_Score Plants_Bio_Score Plants_Int_Score Plants_Psy_Score Dolls_Bio_Score  Dolls_Int_Score Dolls_Psy_Score Humans_Bio_Score Humans_Int_Score Humans_Psy_Score Cats_Bio_Score Cats_Int_Score  Cats_Psy_Score Plants_Bio_Score Plants_Int_Score Plants_Psy_Score Dolls_Bio_Score Dolls_Int_Score Dolls_Psy_Score  Parent_education Parent_age_group  /PRINT=SPEARMAN TWOTAIL NOSIG  /MISSING=PAIRWISE.  CORRELATIONS  /VARIABLES=Humans_Bio_Score Humans_Int_Score Humans_Psy_Score Cats_Bio_Score Cats_Int_Score Cats_Psy_Score Plants_Bio_Score  Plants_Int_score Plants_Psy_Score Child_age_self  /PRINT=TWOTAIL NOSIG  /MISSING=PAIRWISE.  *Children’s DVA-exposure and their ontological conceptualization patterns.  CORRELATIONS  /VARIABLES=Average_Living_Bio_Score Average_Living_Int_Score Average_Living_Psy_Score  Average_Technological_Bio_Score Average_Technological_Int_Score Average_Technological_Psy_Score  DVA_Exposure_Score_P_Total Average_Tech_Affinity_Score Average_Tech_Pos_Score Average_Tech_Neg_Score Average_Tech_Exc_Score Average_Tech_Comp_Score  /PRINT=TWOTAIL NOSIG  /MISSING=PAIRWISE.  DESCRIPTIVES VARIABLES=Average_Living_Bio_Score Average_Living_Int_Score Average_Living_Psy_Score  Average_Technological_Bio_Score Average_Technological_Int_Score Average_Technological_Psy_Score  /STATISTICS=MEAN STDDEV.  COMPUTE Interaction_DVA_Exposure_TechAffinity=DVA_Exposure_Score_P_Total * Average_Tech_Affinity_Score.  EXECUTE.  COMPUTE Interaction_DVA_Exposure_TechAffinity_Pos=DVA_Exposure_Score_P_Total * Average_Tech_Pos_Score.  EXECUTE.  COMPUTE Interaction_DVA_Exposure_TechAffinity_Neg=DVA_Exposure_Score_P_Total * Average_Tech_Neg_Score.  EXECUTE.  COMPUTE Interaction_DVA_Exposure_TechAffinity_Exc=DVA_Exposure_Score_P_Total * Average_Tech_Exc_Score.  EXECUTE.  COMPUTE Interaction_DVA_Exposure_TechAffinity_Comp=DVA_Exposure_Score_P_Total * Average_Tech_Comp_Score.  EXECUTE.  REGRESSION  /MISSING LISTWISE  /STATISTICS COEFF OUTS CI(95) R ANOVA COLLIN TOL CHANGE  /CRITERIA=PIN(.05) POUT(.10)  /NOORIGIN  /DEPENDENT Average_Living_Bio_Score  /METHOD=ENTER Child_gender_self Child_age_self  /METHOD=ENTER DVA_Exposure_Score_P_Total  /METHOD=ENTER Average_Tech_Affinity_Score  /METHOD=ENTER Interaction_DVA_Exposure_TechAffinity.  REGRESSION  /MISSING LISTWISE  /STATISTICS COEFF OUTS CI(95) R ANOVA COLLIN TOL CHANGE  /CRITERIA=PIN(.05) POUT(.10)  /NOORIGIN  /DEPENDENT Average_Living_Bio_Score  /METHOD=ENTER Child_gender_self Child_age_self  /METHOD=ENTER DVA_Exposure_Score_P_Total  /METHOD=ENTER Average_Tech_Pos_Score  /METHOD=ENTER Interaction_DVA_Exposure_TechAffinity_Pos.  REGRESSION  /MISSING LISTWISE  /STATISTICS COEFF OUTS CI(95) R ANOVA COLLIN TOL CHANGE  /CRITERIA=PIN(.05) POUT(.10)  /NOORIGIN  /DEPENDENT Average_Living_Bio_Score  /METHOD=ENTER Child_gender_self Child_age_self  /METHOD=ENTER DVA_Exposure_Score_P_Total  /METHOD=ENTER Average_Tech_Neg_Score  /METHOD=ENTER Interaction_DVA_Exposure_TechAffinity_Neg.  REGRESSION  /MISSING LISTWISE  /STATISTICS COEFF OUTS CI(95) R ANOVA COLLIN TOL CHANGE  /CRITERIA=PIN(.05) POUT(.10)  /NOORIGIN  /DEPENDENT Average_Living_Bio_Score  /METHOD=ENTER Child_gender_self Child_age_self  /METHOD=ENTER DVA_Exposure_Score_P_Total  /METHOD=ENTER Average_Tech_Exc_Score  /METHOD=ENTER Interaction_DVA_Exposure_TechAffinity_Exc.  REGRESSION  /MISSING LISTWISE  /STATISTICS COEFF OUTS CI(95) R ANOVA COLLIN TOL CHANGE  /CRITERIA=PIN(.05) POUT(.10)  /NOORIGIN  /DEPENDENT Average_Living_Bio_Score  /METHOD=ENTER Child_gender_self Child_age_self  /METHOD=ENTER DVA_Exposure_Score_P_Total  /METHOD=ENTER Average_Tech_Comp_Score  /METHOD=ENTER Interaction_DVA_Exposure_TechAffinity_Comp.  REGRESSION  /MISSING LISTWISE  /STATISTICS COEFF OUTS CI(95) R ANOVA COLLIN TOL CHANGE  /CRITERIA=PIN(.05) POUT(.10)  /NOORIGIN  /DEPENDENT Average_Living_Int_Score  /METHOD=ENTER Child_gender_self Child_age_self  /METHOD=ENTER DVA_Exposure_Score_P_Total  /METHOD=ENTER Average_Tech_Affinity_Score  /METHOD=ENTER Interaction_DVA_Exposure_TechAffinity.  REGRESSION  /MISSING LISTWISE  /STATISTICS COEFF OUTS CI(95) R ANOVA COLLIN TOL CHANGE  /CRITERIA=PIN(.05) POUT(.10)  /NOORIGIN  /DEPENDENT Average_Living_Int_Score  /METHOD=ENTER Child_gender_self Child_age_self  /METHOD=ENTER DVA_Exposure_Score_P_Total  /METHOD=ENTER Average_Tech_Pos_Score  /METHOD=ENTER Interaction_DVA_Exposure_TechAffinity_Pos.  REGRESSION  /MISSING LISTWISE  /STATISTICS COEFF OUTS CI(95) R ANOVA COLLIN TOL CHANGE  /CRITERIA=PIN(.05) POUT(.10)  /NOORIGIN  /DEPENDENT Average_Living_Int_Score  /METHOD=ENTER Child_gender_self Child_age_self  /METHOD=ENTER DVA_Exposure_Score_P_Total  /METHOD=ENTER Average_Tech_Neg_Score  /METHOD=ENTER Interaction_DVA_Exposure_TechAffinity_Neg.  REGRESSION  /MISSING LISTWISE  /STATISTICS COEFF OUTS CI(95) R ANOVA COLLIN TOL CHANGE  /CRITERIA=PIN(.05) POUT(.10)  /NOORIGIN  /DEPENDENT Average_Living_Int_Score  /METHOD=ENTER Child_gender_self Child_age_self  /METHOD=ENTER DVA_Exposure_Score_P_Total  /METHOD=ENTER Average_Tech_Exc_Score  /METHOD=ENTER Interaction_DVA_Exposure_TechAffinity_Exc.  REGRESSION  /MISSING LISTWISE  /STATISTICS COEFF OUTS CI(95) R ANOVA COLLIN TOL CHANGE  /CRITERIA=PIN(.05) POUT(.10)  /NOORIGIN  /DEPENDENT Average_Living_Int_Score  /METHOD=ENTER Child_gender_self Child_age_self  /METHOD=ENTER DVA_Exposure_Score_P_Total  /METHOD=ENTER Average_Tech_Comp_Score  /METHOD=ENTER Interaction_DVA_Exposure_TechAffinity_Comp.  REGRESSION  /MISSING LISTWISE  /STATISTICS COEFF OUTS CI(95) R ANOVA COLLIN TOL CHANGE  /CRITERIA=PIN(.05) POUT(.10)  /NOORIGIN  /DEPENDENT Average_Living_Psy_Score  /METHOD=ENTER Child_gender_self Child_age_self  /METHOD=ENTER DVA_Exposure_Score_P_Total  /METHOD=ENTER Average_Tech_Affinity_Score  /METHOD=ENTER Interaction_DVA_Exposure_TechAffinity.  REGRESSION  /MISSING LISTWISE  /STATISTICS COEFF OUTS CI(95) R ANOVA COLLIN TOL CHANGE  /CRITERIA=PIN(.05) POUT(.10)  /NOORIGIN  /DEPENDENT Average_Living_Psy_Score  /METHOD=ENTER Child_gender_self Child_age_self  /METHOD=ENTER DVA_Exposure_Score_P_Total  /METHOD=ENTER Average_Tech_Pos_Score  /METHOD=ENTER Interaction_DVA_Exposure_TechAffinity_Pos.  REGRESSION  /MISSING LISTWISE  /STATISTICS COEFF OUTS CI(95) R ANOVA COLLIN TOL CHANGE  /CRITERIA=PIN(.05) POUT(.10)  /NOORIGIN  /DEPENDENT Average_Living_Psy_Score  /METHOD=ENTER Child_gender_self Child_age_self  /METHOD=ENTER DVA_Exposure_Score_P_Total  /METHOD=ENTER Average_Tech_Neg_Score  /METHOD=ENTER Interaction_DVA_Exposure_TechAffinity_Neg.  REGRESSION  /MISSING LISTWISE  /STATISTICS COEFF OUTS CI(95) R ANOVA COLLIN TOL CHANGE  /CRITERIA=PIN(.05) POUT(.10)  /NOORIGIN  /DEPENDENT Average_Living_Psy_Score  /METHOD=ENTER Child_gender_self Child_age_self  /METHOD=ENTER DVA_Exposure_Score_P_Total  /METHOD=ENTER Average_Tech_Exc_Score  /METHOD=ENTER Interaction_DVA_Exposure_TechAffinity_Exc.  REGRESSION  /MISSING LISTWISE  /STATISTICS COEFF OUTS CI(95) R ANOVA COLLIN TOL CHANGE  /CRITERIA=PIN(.05) POUT(.10)  /NOORIGIN  /DEPENDENT Average_Living_Psy_Score  /METHOD=ENTER Child_gender_self Child_age_self  /METHOD=ENTER DVA_Exposure_Score_P_Total  /METHOD=ENTER Average_Tech_Comp_Score  /METHOD=ENTER Interaction_DVA_Exposure_TechAffinity_Comp.  REGRESSION  /MISSING LISTWISE  /STATISTICS COEFF OUTS CI(95) R ANOVA COLLIN TOL CHANGE  /CRITERIA=PIN(.05) POUT(.10)  /NOORIGIN  /DEPENDENT Average_Technological_Bio_Score  /METHOD=ENTER Child_gender_self Child_age_self  /METHOD=ENTER DVA_Exposure_Score_P_Total  /METHOD=ENTER Average_Tech_Affinity_Score  /METHOD=ENTER Interaction_DVA_Exposure_TechAffinity.  REGRESSION  /MISSING LISTWISE  /STATISTICS COEFF OUTS CI(95) R ANOVA COLLIN TOL CHANGE  /CRITERIA=PIN(.05) POUT(.10)  /NOORIGIN  /DEPENDENT Average_Technological_Bio_Score  /METHOD=ENTER Child_gender_self Child_age_self  /METHOD=ENTER DVA_Exposure_Score_P_Total  /METHOD=ENTER Average_Tech_Pos_Score  /METHOD=ENTER Interaction_DVA_Exposure_TechAffinity_Pos.  REGRESSION  /MISSING LISTWISE  /STATISTICS COEFF OUTS CI(95) R ANOVA COLLIN TOL CHANGE  /CRITERIA=PIN(.05) POUT(.10)  /NOORIGIN  /DEPENDENT Average_Technological_Bio_Score  /METHOD=ENTER Child_gender_self Child_age_self  /METHOD=ENTER DVA_Exposure_Score_P_Total  /METHOD=ENTER Average_Tech_Neg_Score  /METHOD=ENTER Interaction_DVA_Exposure_TechAffinity_Neg.  REGRESSION  /MISSING LISTWISE  /STATISTICS COEFF OUTS CI(95) R ANOVA COLLIN TOL CHANGE  /CRITERIA=PIN(.05) POUT(.10)  /NOORIGIN  /DEPENDENT Average_Technological_Bio_Score  /METHOD=ENTER Child_gender_self Child_age_self  /METHOD=ENTER DVA_Exposure_Score_P_Total  /METHOD=ENTER Average_Tech_Exc_Score  /METHOD=ENTER Interaction_DVA_Exposure_TechAffinity_Exc.  REGRESSION  /MISSING LISTWISE  /STATISTICS COEFF OUTS CI(95) R ANOVA COLLIN TOL CHANGE  /CRITERIA=PIN(.05) POUT(.10)  /NOORIGIN  /DEPENDENT Average_Technological_Bio_Score  /METHOD=ENTER Child_gender_self Child_age_self  /METHOD=ENTER DVA_Exposure_Score_P_Total  /METHOD=ENTER Average_Tech_Comp_Score  /METHOD=ENTER Interaction_DVA_Exposure_TechAffinity_Comp.  REGRESSION  /MISSING LISTWISE  /STATISTICS COEFF OUTS CI(95) R ANOVA COLLIN TOL CHANGE  /CRITERIA=PIN(.05) POUT(.10)  /NOORIGIN  /DEPENDENT Average_Technological_Int_Score  /METHOD=ENTER Child_gender_self Child_age_self  /METHOD=ENTER DVA_Exposure_Score_P_Total  /METHOD=ENTER Average_Tech_Affinity_Score  /METHOD=ENTER Interaction_DVA_Exposure_TechAffinity.  REGRESSION  /MISSING LISTWISE  /STATISTICS COEFF OUTS CI(95) R ANOVA COLLIN TOL CHANGE  /CRITERIA=PIN(.05) POUT(.10)  /NOORIGIN  /DEPENDENT Average_Technological_Int_Score  /METHOD=ENTER Child_gender_self Child_age_self  /METHOD=ENTER DVA_Exposure_Score_P_Total  /METHOD=ENTER Average_Tech_Pos_Score  /METHOD=ENTER Interaction_DVA_Exposure_TechAffinity_Pos.  REGRESSION  /MISSING LISTWISE  /STATISTICS COEFF OUTS CI(95) R ANOVA COLLIN TOL CHANGE  /CRITERIA=PIN(.05) POUT(.10)  /NOORIGIN  /DEPENDENT Average_Technological_Int_Score  /METHOD=ENTER Child_gender_self Child_age_self  /METHOD=ENTER DVA_Exposure_Score_P_Total  /METHOD=ENTER Average_Tech_Neg_Score  /METHOD=ENTER Interaction_DVA_Exposure_TechAffinity_Neg.  REGRESSION  /MISSING LISTWISE  /STATISTICS COEFF OUTS CI(95) R ANOVA COLLIN TOL CHANGE  /CRITERIA=PIN(.05) POUT(.10)  /NOORIGIN  /DEPENDENT Average_Technological_Int_Score  /METHOD=ENTER Child_gender_self Child_age_self  /METHOD=ENTER DVA_Exposure_Score_P_Total  /METHOD=ENTER Average_Tech_Exc_Score  /METHOD=ENTER Interaction_DVA_Exposure_TechAffinity_Exc.  REGRESSION  /MISSING LISTWISE  /STATISTICS COEFF OUTS CI(95) R ANOVA COLLIN TOL CHANGE  /CRITERIA=PIN(.05) POUT(.10)  /NOORIGIN  /DEPENDENT Average_Technological_Int_Score  /METHOD=ENTER Child_gender_self Child_age_self  /METHOD=ENTER DVA_Exposure_Score_P_Total  /METHOD=ENTER Average_Tech_Comp_Score  /METHOD=ENTER Interaction_DVA_Exposure_TechAffinity_Comp.  REGRESSION  /MISSING LISTWISE  /STATISTICS COEFF OUTS CI(95) R ANOVA COLLIN TOL CHANGE  /CRITERIA=PIN(.05) POUT(.10)  /NOORIGIN  /DEPENDENT Average_Technological_Psy_Score  /METHOD=ENTER Child_gender_self Child_age_self  /METHOD=ENTER DVA_Exposure_Score_P_Total  /METHOD=ENTER Average_Tech_Affinity_Score  /METHOD=ENTER Interaction_DVA_Exposure_TechAffinity.  REGRESSION  /MISSING LISTWISE  /STATISTICS COEFF OUTS CI(95) R ANOVA COLLIN TOL CHANGE  /CRITERIA=PIN(.05) POUT(.10)  /NOORIGIN  /DEPENDENT Average_Technological_Psy_Score  /METHOD=ENTER Child_gender_self Child_age_self  /METHOD=ENTER DVA_Exposure_Score_P_Total  /METHOD=ENTER Average_Tech_Pos_Score  /METHOD=ENTER Interaction_DVA_Exposure_TechAffinity_Pos.  REGRESSION  /MISSING LISTWISE  /STATISTICS COEFF OUTS CI(95) R ANOVA COLLIN TOL CHANGE  /CRITERIA=PIN(.05) POUT(.10)  /NOORIGIN  /DEPENDENT Average_Technological_Psy_Score  /METHOD=ENTER Child_gender_self Child_age_self  /METHOD=ENTER DVA_Exposure_Score_P_Total  /METHOD=ENTER Average_Tech_Neg_Score  /METHOD=ENTER Interaction_DVA_Exposure_TechAffinity_Neg.  REGRESSION  /MISSING LISTWISE  /STATISTICS COEFF OUTS CI(95) R ANOVA COLLIN TOL CHANGE  /CRITERIA=PIN(.05) POUT(.10)  /NOORIGIN  /DEPENDENT Average_Technological_Psy_Score  /METHOD=ENTER Child_gender_self Child_age_self  /METHOD=ENTER DVA_Exposure_Score_P_Total  /METHOD=ENTER Average_Tech_Exc_Score  /METHOD=ENTER Interaction_DVA_Exposure_TechAffinity_Exc.  REGRESSION  /MISSING LISTWISE  /STATISTICS COEFF OUTS CI(95) R ANOVA COLLIN TOL CHANGE  /CRITERIA=PIN(.05) POUT(.10)  /NOORIGIN  /DEPENDENT Average_Technological_Psy_Score  /METHOD=ENTER Child_gender_self Child_age_self  /METHOD=ENTER DVA_Exposure_Score_P_Total  /METHOD=ENTER Average_Tech_Comp_Score  /METHOD=ENTER Interaction_DVA_Exposure_TechAffinity_Comp.  *Children’s DVA-exposure and their ontological conceptualization patterns (supplementary analysis).  COMPUTE Interaction_DVA_Exposure_Gender=DVA_Exposure_Score_P_Total * Child_gender_self.  EXECUTE.  COMPUTE Interaction_TechAffinity_Gender=Child_gender_self * Average_Tech_Affinity_Score.  EXECUTE.  COMPUTE Interaction_DVA_Exposure_Age=DVA_Exposure_Score_P_Total * Child_age_self.  EXECUTE.  COMPUTE Interaction_TechAffinity_Age=Child_age_self * Average_Tech_Affinity_Score.  EXECUTE.  REGRESSION  /MISSING LISTWISE  /STATISTICS COEFF OUTS CI(95) R ANOVA COLLIN TOL CHANGE  /CRITERIA=PIN(.05) POUT(.10)  /NOORIGIN  /DEPENDENT Average_Living_Bio_Score  /METHOD=ENTER Child_gender_self Child_age_self  /METHOD=ENTER DVA_Exposure_Score_P_Total  /METHOD=ENTER Average_Tech_Affinity_Score  /METHOD=ENTER Interaction_DVA_Exposure_TechAffinity  /METHOD=ENTER Interaction_DVA_Exposure_Gender Interaction_TechAffinity_Gender  Interaction_DVA_Exposure_Age Interaction_TechAffinity_Age.  REGRESSION  /MISSING LISTWISE  /STATISTICS COEFF OUTS CI(95) R ANOVA COLLIN TOL CHANGE  /CRITERIA=PIN(.05) POUT(.10)  /NOORIGIN  /DEPENDENT Average_Living_Int_Score  /METHOD=ENTER Child_gender_self Child_age_self  /METHOD=ENTER DVA_Exposure_Score_P_Total  /METHOD=ENTER Average_Tech_Affinity_Score  /METHOD=ENTER Interaction_DVA_Exposure_TechAffinity  /METHOD=ENTER Interaction_DVA_Exposure_Gender Interaction_TechAffinity_Gender  Interaction_DVA_Exposure_Age Interaction_TechAffinity_Age.  REGRESSION  /MISSING LISTWISE  /STATISTICS COEFF OUTS CI(95) R ANOVA COLLIN TOL CHANGE  /CRITERIA=PIN(.05) POUT(.10)  /NOORIGIN  /DEPENDENT Average_Living_Psy_Score  /METHOD=ENTER Child_gender_self Child_age_self  /METHOD=ENTER DVA_Exposure_Score_P_Total  /METHOD=ENTER Average_Tech_Affinity_Score  /METHOD=ENTER Interaction_DVA_Exposure_TechAffinity  /METHOD=ENTER Interaction_DVA_Exposure_Gender Interaction_TechAffinity_Gender  Interaction_DVA_Exposure_Age Interaction_TechAffinity_Age.  REGRESSION  /MISSING LISTWISE  /STATISTICS COEFF OUTS CI(95) R ANOVA COLLIN TOL CHANGE  /CRITERIA=PIN(.05) POUT(.10)  /NOORIGIN  /DEPENDENT Average_Technological_Bio_Score  /METHOD=ENTER Child_gender_self Child_age_self  /METHOD=ENTER DVA_Exposure_Score_P_Total  /METHOD=ENTER Average_Tech_Affinity_Score  /METHOD=ENTER Interaction_DVA_Exposure_TechAffinity  /METHOD=ENTER Interaction_DVA_Exposure_Gender Interaction_TechAffinity_Gender  Interaction_DVA_Exposure_Age Interaction_TechAffinity_Age.  REGRESSION  /MISSING LISTWISE  /STATISTICS COEFF OUTS CI(95) R ANOVA COLLIN TOL CHANGE  /CRITERIA=PIN(.05) POUT(.10)  /NOORIGIN  /DEPENDENT Average_Technological_Int_Score  /METHOD=ENTER Child_gender_self Child_age_self  /METHOD=ENTER DVA_Exposure_Score_P_Total  /METHOD=ENTER Average_Tech_Affinity_Score  /METHOD=ENTER Interaction_DVA_Exposure_TechAffinity  /METHOD=ENTER Interaction_DVA_Exposure_Gender Interaction_TechAffinity_Gender  Interaction_DVA_Exposure_Age Interaction_TechAffinity_Age.  REGRESSION  /MISSING LISTWISE  /STATISTICS COEFF OUTS CI(95) R ANOVA COLLIN TOL CHANGE  /CRITERIA=PIN(.05) POUT(.10)  /NOORIGIN  /DEPENDENT Average_Technological_Psy_Score  /METHOD=ENTER Child_gender_self Child_age_self  /METHOD=ENTER DVA_Exposure_Score_P_Total  /METHOD=ENTER Average_Tech_Affinity_Score  /METHOD=ENTER Interaction_DVA_Exposure_TechAffinity  /METHOD=ENTER Interaction_DVA_Exposure_Gender Interaction_TechAffinity_Gender  Interaction_DVA_Exposure_Age Interaction_TechAffinity_Age.  RECODE Parent_education (1=1) (2=1) (ELSE=0) INTO Parent_education_low.  VARIABLE LABELS Parent_education_low 'Less than high school or high school degree'.  EXECUTE.  RECODE Parent_education (3=1) (ELSE=0) INTO Parent_education_medium.  VARIABLE LABELS Parent_education_medium 'Undergraduate college degree'.  EXECUTE.  RECODE Parent_education (4=1) (5=1) (6=1) (ELSE=0) INTO Parent_education_high.  VARIABLE LABELS Parent_education_high 'Postgraduate college degree, professional degree or doctorate'.  EXECUTE.  COMPUTE Interaction_DVA_Exposure_Parent_education_low=DVA_Exposure_Score_P_Total * Parent_education_low.  EXECUTE.  COMPUTE Interaction_DVA_Exposure_Parent_education_medium=DVA_Exposure_Score_P_Total * Parent_education_medium.  EXECUTE.  COMPUTE Interaction_DVA_Exposure_Parent_education_high=DVA_Exposure_Score_P_Total * Parent_education_high.  EXECUTE.  COMPUTE Interaction_TechAffinity_Parent_education_low=Parent_education_low * Average_Tech_Affinity_Score.  EXECUTE.  COMPUTE Interaction_TechAffinity_Parent_education_medium=Parent_education_medium * Average_Tech_Affinity_Score.  EXECUTE.  COMPUTE Interaction_TechAffinity_Parent_education_high=Parent_education_high * Average_Tech_Affinity_Score.  EXECUTE.  REGRESSION  /MISSING LISTWISE  /STATISTICS COEFF OUTS CI(95) R ANOVA COLLIN TOL CHANGE  /CRITERIA=PIN(.05) POUT(.10)  /NOORIGIN  /DEPENDENT Average_Living_Bio_Score  /METHOD=ENTER Child_gender_self Child_age_self Parent_education_medium Parent_education_high  /METHOD=ENTER DVA_Exposure_Score_P_Total  /METHOD=ENTER Average_Tech_Affinity_Score  /METHOD=ENTER Interaction_DVA_Exposure_TechAffinity  /METHOD=ENTER Interaction_DVA_Exposure_Parent_education_medium  Interaction_DVA_Exposure_Parent_education_high Interaction_TechAffinity_Parent_education_medium  Interaction_TechAffinity_Parent_education_high.  REGRESSION  /MISSING LISTWISE  /STATISTICS COEFF OUTS CI(95) R ANOVA COLLIN TOL CHANGE  /CRITERIA=PIN(.05) POUT(.10)  /NOORIGIN  /DEPENDENT Average_Living_Int_Score  /METHOD=ENTER Child_gender_self Child_age_self Parent_education_medium Parent_education_high  /METHOD=ENTER DVA_Exposure_Score_P_Total  /METHOD=ENTER Average_Tech_Affinity_Score  /METHOD=ENTER Interaction_DVA_Exposure_TechAffinity  /METHOD=ENTER Interaction_DVA_Exposure_Parent_education_medium  Interaction_DVA_Exposure_Parent_education_high Interaction_TechAffinity_Parent_education_medium  Interaction_TechAffinity_Parent_education_high.  REGRESSION  /MISSING LISTWISE  /STATISTICS COEFF OUTS CI(95) R ANOVA COLLIN TOL CHANGE  /CRITERIA=PIN(.05) POUT(.10)  /NOORIGIN  /DEPENDENT Average_Living_Psy_Score  /METHOD=ENTER Child_gender_self Child_age_self Parent_education_medium Parent_education_high  /METHOD=ENTER DVA_Exposure_Score_P_Total  /METHOD=ENTER Average_Tech_Affinity_Score  /METHOD=ENTER Interaction_DVA_Exposure_TechAffinity  /METHOD=ENTER Interaction_DVA_Exposure_Parent_education_medium  Interaction_DVA_Exposure_Parent_education_high Interaction_TechAffinity_Parent_education_medium  Interaction_TechAffinity_Parent_education_high.  REGRESSION  /MISSING LISTWISE  /STATISTICS COEFF OUTS CI(95) R ANOVA COLLIN TOL CHANGE  /CRITERIA=PIN(.05) POUT(.10)  /NOORIGIN  /DEPENDENT Average_Technological_Bio_Score  /METHOD=ENTER Child_gender_self Child_age_self Parent_education_medium Parent_education_high  /METHOD=ENTER DVA_Exposure_Score_P_Total  /METHOD=ENTER Average_Tech_Affinity_Score  /METHOD=ENTER Interaction_DVA_Exposure_TechAffinity  /METHOD=ENTER Interaction_DVA_Exposure_Parent_education_medium  Interaction_DVA_Exposure_Parent_education_high Interaction_TechAffinity_Parent_education_medium  Interaction_TechAffinity_Parent_education_high.  REGRESSION  /MISSING LISTWISE  /STATISTICS COEFF OUTS CI(95) R ANOVA COLLIN TOL CHANGE  /CRITERIA=PIN(.05) POUT(.10)  /NOORIGIN  /DEPENDENT Average_Technological_Int_Score  /METHOD=ENTER Child_gender_self Child_age_self Parent_education_medium Parent_education_high  /METHOD=ENTER DVA_Exposure_Score_P_Total  /METHOD=ENTER Average_Tech_Affinity_Score  /METHOD=ENTER Interaction_DVA_Exposure_TechAffinity  /METHOD=ENTER Interaction_DVA_Exposure_Parent_education_medium  Interaction_DVA_Exposure_Parent_education_high Interaction_TechAffinity_Parent_education_medium  Interaction_TechAffinity_Parent_education_high.  REGRESSION  /MISSING LISTWISE  /STATISTICS COEFF OUTS CI(95) R ANOVA COLLIN TOL CHANGE  /CRITERIA=PIN(.05) POUT(.10)  /NOORIGIN  /DEPENDENT Average_Technological_Psy_Score  /METHOD=ENTER Child_gender_self Child_age_self Parent_education_medium Parent_education_high  /METHOD=ENTER DVA_Exposure_Score_P_Total  /METHOD=ENTER Average_Tech_Affinity_Score  /METHOD=ENTER Interaction_DVA_Exposure_TechAffinity  /METHOD=ENTER Interaction_DVA_Exposure_Parent_education_medium  Interaction_DVA_Exposure_Parent_education_high Interaction_TechAffinity_Parent_education_medium  Interaction_TechAffinity_Parent_education_high.  *Ontological differentiations between biological and technological entities.  DESCRIPTIVES VARIABLES=Humans_Bio_Score Cats_Bio_Score Voice_Assistants_Bio_Score  Computers_Bio_Score Plants_Bio_Score Smartphones_Bio_Score Dolls_Bio_Score Robots_Bio_Score  Drones_Bio_Score Humans_Int_Score Cats_Int_Score Voice_Assistants_Int_Score Computers_Int_Score  Plants_Int_Score Smartphones_Int_Score Dolls_Int_Score Robots_Int_Score Drones_Int_Score  Humans_Psy_Score Cats_Psy_Score Voice_Assistants_Psy_Score Computers_Psy_Score Plants_Psy_Score  Smartphones_Psy_Score Dolls_Psy_Score Robots_Psy_Score Drones_Psy_Score  /SAVE.  COMPUTE Average_Living_Int_Score_STANDARDIZED=(ZHumans_Int_Score + ZCats_Int_Score + ZPlants_Int_Score) / 3.  EXECUTE.  COMPUTE Average_Living_Psy_Score_STANDARDIZED=(ZHumans_Psy_Score + ZCats_Psy_Score + ZPlants_Psy_Score) / 3.  EXECUTE.  COMPUTE Average_Living_Bio_Score_STANDARDIZED=(ZHumans_Bio_Score + ZCats_Bio_Score + ZPlants_Bio_Score) / 3.  EXECUTE.  COMPUTE Average_Technological_Int_Score_STANDARDIZED=(ZVoice_Assistants_Int_Score + ZComputers_Int_Score +  ZSmartphones_Int_Score + ZRobots_Int_Score + ZDrones_Int_Score) / 5.  EXECUTE.  COMPUTE Average_Technological_Psy_Score_STANDARDIZED=(ZVoice_Assistants_Psy_Score + ZComputers_Psy_Score +  ZSmartphones_Psy_Score + ZRobots_Psy_Score + ZDrones_Psy_Score) / 5.  EXECUTE.  COMPUTE Average_Technological_Bio_Score_STANDARDIZED=(ZVoice_Assistants_Bio_Score + ZComputers_Bio_Score +  ZSmartphones_Bio_Score + ZRobots_Bio_Score + ZDrones_Bio_Score) / 5.  EXECUTE.  COMPUTE Diff_LivTech_Int_Scores=ABS(Average_Living_Int_Score_STANDARDIZED - Average_Technological_Int_Score_STANDARDIZED).  EXECUTE.  COMPUTE Diff_LivTech_Psy_Scores=ABS(Average_Living_Psy_Score_STANDARDIZED - Average_Technological_Psy_Score_STANDARDIZED).  EXECUTE.  COMPUTE Diff_LivTech_Bio_Scores=ABS(Average_Living_Bio_Score_STANDARDIZED - Average_Technological_Bio_Score_STANDARDIZED).  EXECUTE.  DESCRIPTIVES VARIABLES=Average_Living_Bio_Score Average_Living_Int_Score Average_Living_Psy_Score  Average_Technological_Bio_Score Average_Technological_Int_Score Average_Technological_Psy_Score  /SAVE.  COMPUTE Diff_LivTech_Int_Scores_2=ABS(ZAverage_Living_Int_Score - ZAverage_Technological_Int_Score).  EXECUTE.  COMPUTE Diff_LivTech_Psy_Scores_2=ABS(ZAverage_Living_Psy_Score - ZAverage_Technological_Psy_Score).  EXECUTE.  COMPUTE Diff_LivTech_Bio_Scores_2=ABS(ZAverage_Living_Bio_Score - ZAverage_Technological_Bio_Score).  EXECUTE.  GLM Diff_LivTech_Int_Scores Diff_LivTech_Psy_Scores Diff_LivTech_Bio_Scores  /WSFACTOR=Ont_Differences 3 Repeated  /METHOD=SSTYPE(3)  /PLOT=PROFILE(Ont_Differences) TYPE=LINE ERRORBAR=CI MEANREFERENCE=NO YAXIS=AUTO  /CRITERIA=ALPHA(.05)  /WSDESIGN=Ont_Differences.  GLM Diff_LivTech_Int_Scores_2 Diff_LivTech_Psy_Scores_2 Diff_LivTech_Bio_Scores_2  /WSFACTOR=Ont_Differences 3 Repeated  /METHOD=SSTYPE(3)  /PLOT=PROFILE(Ont_Differences) TYPE=LINE ERRORBAR=CI MEANREFERENCE=NO YAXIS=AUTO  /CRITERIA=ALPHA(.05)  /WSDESIGN=Ont_Differences.  GLM Diff_LivTech_Int_Scores Diff_LivTech_Psy_Scores Diff_LivTech_Bio_Scores BY DVA_Exposure_Group  /WSFACTOR=Ont_Differences 3 Repeated  /METHOD=SSTYPE(3)  /PLOT=PROFILE(Ont_Differences*DVA_Exposure_Group) TYPE=LINE ERRORBAR=CI MEANREFERENCE=NO  YAXIS=AUTO  /CRITERIA=ALPHA(.05)  /WSDESIGN=Ont_Differences  /DESIGN=DVA_Exposure_Group.  GLM Diff_LivTech_Int_Scores_2 Diff_LivTech_Psy_Scores_2 Diff_LivTech_Bio_Scores_2 BY DVA_Exposure_Group  /WSFACTOR=Ont_Differences 3 Repeated  /METHOD=SSTYPE(3)  /PLOT=PROFILE(Ont_Differences*DVA_Exposure_Group) TYPE=LINE ERRORBAR=CI MEANREFERENCE=NO  YAXIS=AUTO  /CRITERIA=ALPHA(.05)  /WSDESIGN=Ont_Differences  /DESIGN=DVA_Exposure_Group.  T-TEST GROUPS=DVA_Exposure_Group(0 1)  /MISSING=ANALYSIS  /VARIABLES=Diff_LivTech_Bio_Scores_2  /CRITERIA=CI(.95).  T-TEST GROUPS=DVA_Exposure_Group(0 1)  /MISSING=ANALYSIS  /VARIABLES=Diff_LivTech_Int_Scores_2  /CRITERIA=CI(.95).  T-TEST GROUPS=DVA_Exposure_Group(0 1)  /MISSING=ANALYSIS  /VARIABLES=Diff_LivTech_Psy_Scores_2  /CRITERIA=CI(.95).  T-TEST GROUPS=DVA_Exposure_Group(0 1)  /MISSING=ANALYSIS  /VARIABLES=ZAverage_Living_Psy_Score  /CRITERIA=CI(.95).  T-TEST GROUPS=DVA_Exposure_Group(0 1)  /MISSING=ANALYSIS  /VARIABLES=ZAverage_Technological_Psy_Score  /CRITERIA=CI(.95).  GLM Diff_LivTech_Int_Scores Diff_LivTech_Psy_Scores Diff_LivTech_Bio_Scores BY DVA_Exposure_Group  WITH Average_Tech_Affinity_Score  /WSFACTOR=Ont_Differences 3 Repeated  /CONTRAST(DVA_Exposure_Group)=Repeated  /METHOD=SSTYPE(3)  /PLOT=PROFILE(Ont_Differences*DVA_Exposure_Group) TYPE=LINE ERRORBAR=CI MEANREFERENCE=NO  YAXIS=AUTO  /CRITERIA=ALPHA(.05)  /WSDESIGN=Ont_Differences  /DESIGN=Average_Tech_Affinity_Score DVA_Exposure_Group.  GLM Diff_LivTech_Int_Scores_2 Diff_LivTech_Psy_Scores_2 Diff_LivTech_Bio_Scores_2 BY DVA_Exposure_Group  WITH Average_Tech_Affinity_Score  /WSFACTOR=Ont_Differences 3 Repeated  /CONTRAST(DVA_Exposure_Group)=Repeated  /METHOD=SSTYPE(3)  /PLOT=PROFILE(Ont_Differences*DVA_Exposure_Group) TYPE=LINE ERRORBAR=CI MEANREFERENCE=NO  YAXIS=AUTO  /CRITERIA=ALPHA(.05)  /WSDESIGN=Ont_Differences  /DESIGN=Average_Tech_Affinity_Score DVA_Exposure_Group.  REGRESSION  /MISSING LISTWISE  /STATISTICS COEFF OUTS CI(95) R ANOVA COLLIN TOL CHANGE  /CRITERIA=PIN(.05) POUT(.10)  /NOORIGIN  /DEPENDENT Diff_LivTech_Bio_Scores_2  /METHOD=ENTER Child_gender_self Child_age_self  /METHOD=ENTER DVA_Exposure_Score_P_Total  /METHOD=ENTER Average_Tech_Affinity_Score  /METHOD=ENTER Interaction_DVA_Exposure_TechAffinity.  REGRESSION  /MISSING LISTWISE  /STATISTICS COEFF OUTS CI(95) R ANOVA COLLIN TOL CHANGE  /CRITERIA=PIN(.05) POUT(.10)  /NOORIGIN  /DEPENDENT Diff_LivTech_Int_Scores_2  /METHOD=ENTER Child_gender_self Child_age_self  /METHOD=ENTER DVA_Exposure_Score_P_Total  /METHOD=ENTER Average_Tech_Affinity_Score  /METHOD=ENTER Interaction_DVA_Exposure_TechAffinity.  REGRESSION  /MISSING LISTWISE  /STATISTICS COEFF OUTS CI(95) R ANOVA COLLIN TOL CHANGE  /CRITERIA=PIN(.05) POUT(.10)  /NOORIGIN  /DEPENDENT Diff_LivTech_Psy_Scores_2  /METHOD=ENTER Child_gender_self Child_age_self  /METHOD=ENTER DVA_Exposure_Score_P_Total  /METHOD=ENTER Average_Tech_Affinity_Score  /METHOD=ENTER Interaction_DVA_Exposure_TechAffinity.  COMPUTE Group_Interaction_DVA_Exposure_TechAffinity=DVA_Exposure_Group * Average_Tech_Affinity_Score.  EXECUTE.  REGRESSION  /MISSING LISTWISE  /STATISTICS COEFF OUTS CI(95) R ANOVA COLLIN TOL CHANGE  /CRITERIA=PIN(.05) POUT(.10)  /NOORIGIN  /DEPENDENT Diff_LivTech_Bio_Scores_2  /METHOD=ENTER Child_gender_self Child_age_self  /METHOD=ENTER DVA_Exposure_Group  /METHOD=ENTER Average_Tech_Affinity_Score  /METHOD=ENTER Group_Interaction_DVA_Exposure_TechAffinity.  REGRESSION  /MISSING LISTWISE  /STATISTICS COEFF OUTS CI(95) R ANOVA COLLIN TOL CHANGE  /CRITERIA=PIN(.05) POUT(.10)  /NOORIGIN  /DEPENDENT Diff_LivTech_Int_Scores_2  /METHOD=ENTER Child_gender_self Child_age_self  /METHOD=ENTER DVA_Exposure_Group  /METHOD=ENTER Average_Tech_Affinity_Score  /METHOD=ENTER Group_Interaction_DVA_Exposure_TechAffinity.  REGRESSION  /MISSING LISTWISE  /STATISTICS COEFF OUTS CI(95) R ANOVA COLLIN TOL CHANGE  /CRITERIA=PIN(.05) POUT(.10)  /NOORIGIN  /DEPENDENT Diff_LivTech_Psy_Scores_2  /METHOD=ENTER Child_gender_self Child_age_self  /METHOD=ENTER DVA_Exposure_Group  /METHOD=ENTER Average_Tech_Affinity_Score  /METHOD=ENTER Group_Interaction_DVA_Exposure_TechAffinity.  REGRESSION  /MISSING LISTWISE  /STATISTICS COEFF OUTS CI(95) R ANOVA COLLIN TOL CHANGE  /CRITERIA=PIN(.05) POUT(.10)  /NOORIGIN  /DEPENDENT Diff_LivTech_Bio_Scores  /METHOD=ENTER Child_gender_self Child_age_self  /METHOD=ENTER DVA_Exposure_Score_P_Total  /METHOD=ENTER Average_Tech_Affinity_Score  /METHOD=ENTER Interaction_DVA_Exposure_TechAffinity.  REGRESSION  /MISSING LISTWISE  /STATISTICS COEFF OUTS CI(95) R ANOVA COLLIN TOL CHANGE  /CRITERIA=PIN(.05) POUT(.10)  /NOORIGIN  /DEPENDENT Diff_LivTech_Int_Scores  /METHOD=ENTER Child_gender_self Child_age_self  /METHOD=ENTER DVA_Exposure_Score_P_Total  /METHOD=ENTER Average_Tech_Affinity_Score  /METHOD=ENTER Interaction_DVA_Exposure_TechAffinity.  REGRESSION  /MISSING LISTWISE  /STATISTICS COEFF OUTS CI(95) R ANOVA COLLIN TOL CHANGE  /CRITERIA=PIN(.05) POUT(.10)  /NOORIGIN  /DEPENDENT Diff_LivTech_Psy_Scores  /METHOD=ENTER Child_gender_self Child_age_self  /METHOD=ENTER DVA_Exposure_Score_P_Total  /METHOD=ENTER Average_Tech_Affinity_Score  /METHOD=ENTER Interaction_DVA_Exposure_TechAffinity.  REGRESSION  /MISSING LISTWISE  /STATISTICS COEFF OUTS CI(95) R ANOVA COLLIN TOL CHANGE  /CRITERIA=PIN(.05) POUT(.10)  /NOORIGIN  /DEPENDENT Diff_LivTech_Bio_Scores  /METHOD=ENTER Child_gender_self Child_age_self  /METHOD=ENTER DVA_Exposure_Group  /METHOD=ENTER Average_Tech_Affinity_Score  /METHOD=ENTER Group_Interaction_DVA_Exposure_TechAffinity.  REGRESSION  /MISSING LISTWISE  /STATISTICS COEFF OUTS CI(95) R ANOVA COLLIN TOL CHANGE  /CRITERIA=PIN(.05) POUT(.10)  /NOORIGIN  /DEPENDENT Diff_LivTech_Int_Scores  /METHOD=ENTER Child_gender_self Child_age_self  /METHOD=ENTER DVA_Exposure_Group  /METHOD=ENTER Average_Tech_Affinity_Score  /METHOD=ENTER Group_Interaction_DVA_Exposure_TechAffinity.  REGRESSION  /MISSING LISTWISE  /STATISTICS COEFF OUTS CI(95) R ANOVA COLLIN TOL CHANGE  /CRITERIA=PIN(.05) POUT(.10)  /NOORIGIN  /DEPENDENT Diff_LivTech_Psy_Scores  /METHOD=ENTER Child_gender_self Child_age_self  /METHOD=ENTER DVA_Exposure_Group  /METHOD=ENTER Average_Tech_Affinity_Score  /METHOD=ENTER Group_Interaction_DVA_Exposure_TechAffinity.  *Ontological differentiations between biological and technological entities (supplementary analysis).  REGRESSION  /MISSING LISTWISE  /STATISTICS COEFF OUTS CI(95) R ANOVA COLLIN TOL CHANGE  /CRITERIA=PIN(.05) POUT(.10)  /NOORIGIN  /DEPENDENT Diff_LivTech_Bio_Scores_2  /METHOD=ENTER Child_gender_self Child_age_self  /METHOD=ENTER DVA_Exposure_Score_P_Total  /METHOD=ENTER Average_Tech_Affinity_Score  /METHOD=ENTER Interaction_DVA_Exposure_TechAffinity  /METHOD=ENTER Interaction_DVA_Exposure_Gender Interaction_TechAffinity_Gender  Interaction_DVA_Exposure_Age Interaction_TechAffinity_Age.  REGRESSION  /MISSING LISTWISE  /STATISTICS COEFF OUTS CI(95) R ANOVA COLLIN TOL CHANGE  /CRITERIA=PIN(.05) POUT(.10)  /NOORIGIN  /DEPENDENT Diff_LivTech_Int_Scores_2  /METHOD=ENTER Child_gender_self Child_age_self  /METHOD=ENTER DVA_Exposure_Score_P_Total  /METHOD=ENTER Average_Tech_Affinity_Score  /METHOD=ENTER Interaction_DVA_Exposure_TechAffinity  /METHOD=ENTER Interaction_DVA_Exposure_Gender Interaction_TechAffinity_Gender  Interaction_DVA_Exposure_Age Interaction_TechAffinity_Age.  REGRESSION  /MISSING LISTWISE  /STATISTICS COEFF OUTS CI(95) R ANOVA COLLIN TOL CHANGE  /CRITERIA=PIN(.05) POUT(.10)  /NOORIGIN  /DEPENDENT Diff_LivTech_Psy_Scores_2  /METHOD=ENTER Child_gender_self Child_age_self  /METHOD=ENTER DVA_Exposure_Score_P_Total  /METHOD=ENTER Average_Tech_Affinity_Score  /METHOD=ENTER Interaction_DVA_Exposure_TechAffinity  /METHOD=ENTER Interaction_DVA_Exposure_Gender Interaction_TechAffinity_Gender  Interaction_DVA_Exposure_Age Interaction_TechAffinity_Age.  REGRESSION  /MISSING LISTWISE  /STATISTICS COEFF OUTS CI(95) R ANOVA COLLIN TOL CHANGE  /CRITERIA=PIN(.05) POUT(.10)  /NOORIGIN  /DEPENDENT Diff_LivTech_Bio_Scores  /METHOD=ENTER Child_gender_self Child_age_self  /METHOD=ENTER DVA_Exposure_Score_P_Total  /METHOD=ENTER Average_Tech_Affinity_Score  /METHOD=ENTER Interaction_DVA_Exposure_TechAffinity  /METHOD=ENTER Interaction_DVA_Exposure_Gender Interaction_TechAffinity_Gender  Interaction_DVA_Exposure_Age Interaction_TechAffinity_Age.  REGRESSION  /MISSING LISTWISE  /STATISTICS COEFF OUTS CI(95) R ANOVA COLLIN TOL CHANGE  /CRITERIA=PIN(.05) POUT(.10)  /NOORIGIN  /DEPENDENT Diff_LivTech_Int_Scores  /METHOD=ENTER Child_gender_self Child_age_self  /METHOD=ENTER DVA_Exposure_Score_P_Total  /METHOD=ENTER Average_Tech_Affinity_Score  /METHOD=ENTER Interaction_DVA_Exposure_TechAffinity  /METHOD=ENTER Interaction_DVA_Exposure_Gender Interaction_TechAffinity_Gender  Interaction_DVA_Exposure_Age Interaction_TechAffinity_Age.  REGRESSION  /MISSING LISTWISE  /STATISTICS COEFF OUTS CI(95) R ANOVA COLLIN TOL CHANGE  /CRITERIA=PIN(.05) POUT(.10)  /NOORIGIN  /DEPENDENT Diff_LivTech_Psy_Scores  /METHOD=ENTER Child_gender_self Child_age_self  /METHOD=ENTER DVA_Exposure_Score_P_Total  /METHOD=ENTER Average_Tech_Affinity_Score  /METHOD=ENTER Interaction_DVA_Exposure_TechAffinity  /METHOD=ENTER Interaction_DVA_Exposure_Gender Interaction_TechAffinity_Gender  Interaction_DVA_Exposure_Age Interaction_TechAffinity_Age.  REGRESSION  /MISSING LISTWISE  /STATISTICS COEFF OUTS CI(95) R ANOVA COLLIN TOL CHANGE  /CRITERIA=PIN(.05) POUT(.10)  /NOORIGIN  /DEPENDENT Diff_LivTech_Bio_Scores_2  /METHOD=ENTER Child_gender_self Child_age_self Parent_education_medium Parent_education_high  /METHOD=ENTER DVA_Exposure_Score_P_Total  /METHOD=ENTER Average_Tech_Affinity_Score  /METHOD=ENTER Interaction_DVA_Exposure_TechAffinity  /METHOD=ENTER Interaction_DVA_Exposure_Parent_education_medium  Interaction_DVA_Exposure_Parent_education_high Interaction_TechAffinity_Parent_education_medium  Interaction_TechAffinity_Parent_education_high.  REGRESSION  /MISSING LISTWISE  /STATISTICS COEFF OUTS CI(95) R ANOVA COLLIN TOL CHANGE  /CRITERIA=PIN(.05) POUT(.10)  /NOORIGIN  /DEPENDENT Diff_LivTech_Int_Scores_2  /METHOD=ENTER Child_gender_self Child_age_self Parent_education_medium Parent_education_high  /METHOD=ENTER DVA_Exposure_Score_P_Total  /METHOD=ENTER Average_Tech_Affinity_Score  /METHOD=ENTER Interaction_DVA_Exposure_TechAffinity  /METHOD=ENTER Interaction_DVA_Exposure_Parent_education_medium  Interaction_DVA_Exposure_Parent_education_high Interaction_TechAffinity_Parent_education_medium  Interaction_TechAffinity_Parent_education_high.  REGRESSION  /MISSING LISTWISE  /STATISTICS COEFF OUTS CI(95) R ANOVA COLLIN TOL CHANGE  /CRITERIA=PIN(.05) POUT(.10)  /NOORIGIN  /DEPENDENT Diff_LivTech_Psy_Scores_2  /METHOD=ENTER Child_gender_self Child_age_self Parent_education_medium Parent_education_high  /METHOD=ENTER DVA_Exposure_Score_P_Total  /METHOD=ENTER Average_Tech_Affinity_Score  /METHOD=ENTER Interaction_DVA_Exposure_TechAffinity  /METHOD=ENTER Interaction_DVA_Exposure_Parent_education_medium  Interaction_DVA_Exposure_Parent_education_high Interaction_TechAffinity_Parent_education_medium  Interaction_TechAffinity_Parent_education_high.  REGRESSION  /MISSING LISTWISE  /STATISTICS COEFF OUTS CI(95) R ANOVA COLLIN TOL CHANGE  /CRITERIA=PIN(.05) POUT(.10)  /NOORIGIN  /DEPENDENT Diff_LivTech_Bio_Scores  /METHOD=ENTER Child_gender_self Child_age_self Parent_education_medium Parent_education_high  /METHOD=ENTER DVA_Exposure_Score_P_Total  /METHOD=ENTER Average_Tech_Affinity_Score  /METHOD=ENTER Interaction_DVA_Exposure_TechAffinity  /METHOD=ENTER Interaction_DVA_Exposure_Parent_education_medium  Interaction_DVA_Exposure_Parent_education_high Interaction_TechAffinity_Parent_education_medium  Interaction_TechAffinity_Parent_education_high.  REGRESSION  /MISSING LISTWISE  /STATISTICS COEFF OUTS CI(95) R ANOVA COLLIN TOL CHANGE  /CRITERIA=PIN(.05) POUT(.10)  /NOORIGIN  /DEPENDENT Diff_LivTech_Int_Scores  /METHOD=ENTER Child_gender_self Child_age_self Parent_education_medium Parent_education_high  /METHOD=ENTER DVA_Exposure_Score_P_Total  /METHOD=ENTER Average_Tech_Affinity_Score  /METHOD=ENTER Interaction_DVA_Exposure_TechAffinity  /METHOD=ENTER Interaction_DVA_Exposure_Parent_education_medium  Interaction_DVA_Exposure_Parent_education_high Interaction_TechAffinity_Parent_education_medium  Interaction_TechAffinity_Parent_education_high.  REGRESSION  /MISSING LISTWISE  /STATISTICS COEFF OUTS CI(95) R ANOVA COLLIN TOL CHANGE  /CRITERIA=PIN(.05) POUT(.10)  /NOORIGIN  /DEPENDENT Diff_LivTech_Psy_Scores  /METHOD=ENTER Child_gender_self Child_age_self Parent_education_medium Parent_education_high  /METHOD=ENTER DVA_Exposure_Score_P_Total  /METHOD=ENTER Average_Tech_Affinity_Score  /METHOD=ENTER Interaction_DVA_Exposure_TechAffinity  /METHOD=ENTER Interaction_DVA_Exposure_Parent_education_medium  Interaction_DVA_Exposure_Parent_education_high Interaction_TechAffinity_Parent_education_medium  Interaction_TechAffinity_Parent_education_high. |
| *Notes.* Material shows syntax used for data analysis in *IBM SPSS Statistics 27.0.* |

| Supplementary Material VIII. SPSS syntax |
| --- |
| TITLE: TAQ ESEM Analysis  DATA: FILE = VFS_1_MTURK_Survey_Edited_Data_N143_TAQ.csv;  VARIABLE: NAMES = TAQNeg1-TAQNeg5 TAQPos1-TAQPos5 TAQExc1-TAQExc5 TAQComp1-TAQComp5  DVAEXGR;  USEVARIABLES = TAQNeg1-TAQNeg5 TAQPos1-TAQPos5 TAQExc1-TAQExc5  TAQComp1-TAQComp5;  CATEGORICAL = TAQNeg1-TAQNeg5 TAQPos1-TAQPos5 TAQExc1-TAQExc5  TAQComp1-TAQComp5;  MODEL: Positive_Attitude by  TAQPos1-TAQPos5 TAQNeg1-TAQNeg5~0 TAQExc1-TAQExc5~0 TAQComp1-TAQComp5~0 (*1);  Negative_Attitude by  TAQPos1-TAQPos5~0 TAQNeg1-TAQNeg5 TAQExc1-TAQExc5~0 TAQComp1-TAQComp5~0 (*1);  Excitement by  TAQPos1-TAQPos5~0 TAQNeg1-TAQNeg5~0 TAQExc1-TAQExc5 TAQComp1-TAQComp5~0 (*1);  Competency by  TAQPos1-TAQPos5~0 TAQNeg1-TAQNeg5~0 TAQExc1-TAQExc5~0 TAQComp1-TAQComp5 (*1);  ANALYSIS: ESTIMATOR = WLSMV;  ROTATION = TARGET;  OUTPUT: SAMPSTAT STANDARDIZED RESIDUAL TECH1; |
| *Notes.* Material shows syntax used for data analysis in *Mplus* version 8.5 (Mac). |

References

Asparouhov, T., & Muthén, B. (2009). Exploratory Structural Equation Modeling. *Structural Equation Modeling*, *16*(3), 397–438. https://doi.org/10.1080/10705510903008204

Browne, M. W. (2001). An Overview of Analytic Rotation in Exploratory Factor Analysis. *Multivariate Behavioral Research*, *36*(1), 111–150. https://doi.org/10.1207/S15327906MBR3601_05

Hair, J. F., Tatham, R. L., Anderson, R. E., & Black, W. (1998). *Multivariate Data Analysis* (Fifth). Prentice-Hall International.

Hu, L., & Bentler, P. M. (1999). Cutoff criteria for fit indexes in covariance structure analysis: Conventional criteria versus new alternatives. *Structural Equation Modeling: A Multidisciplinary Journal*, *6*(1), 1–55. https://doi.org/10.1080/10705519909540118

Karrer, K., Glaser, C., Clemens, C., & Bruder, C. (2009). Technikaffinität erfassen: Der Fragebogen TA-EG. *Der Mensch Im Mittelpunkt Technischer Systeme : 8. Berliner Werkstatt Mensch-Maschine-Systeme, 7. Bis 9. Oktober 2009*, 196–201.

Kline, R. B. (2016). *Principles and Practice of Structural Equation Modeling* (Fourth). The Guilford Press.

Marsh, H. W., Morin, A. J. S., Parker, P. D., & Kaur, G. (2014). Exploratory Structural Equation Modeling: An Integration of the Best Features of Exploratory and Confirmatory Factor Analysis. *Annual Review Clinical Psychology*, *10*(1), 85–110. https://doi.org/10.1146/annurev-clinpsy-032813-153700

Morin, A. J. S., Maiano, C., White, R. L., Owen, K. B., Tracey, D., Mascret, N., & Lonsdale, C. (2016). English validation of the short form of the Physical Self-Inventory (PSI-S). *Psychology of Sport and Exercise*, *27*, 180–194. https://doi.org/10.1016/j.psychsport.2016.08.016

Shi, D., & Maydeu-Olivares, A. (2019). The Effect of Estimation Methods on SEM Fit Indices. *Educational and Psychological Measurement*, *80*(3), 421–445. https://doi.org/10.1177/0013164419885164

Tóth-Király, I., Bõthe, B., Rigó, A., & Orosz, G. (2017). An Illustration of the Exploratory Structural Equation Modeling (ESEM) Framework on the Passion Scale. *Frontiers in Psychology*, *8*. https://doi.org/10.3389/fpsyg.2017.01968

Zumbo, B. D., Gadermann, A. M., & Zeisser, C. (2007). Ordinal Versions of Coefficients Alpha and Theta for Likert Rating Scales. *Journal of Modern Applied Statistical Methods*, *6*(1), 4. https://doi.org/10.22237/jmasm/1177992180

Zumbo, B. D., & Kroc, E. (2019). A Measurement Is a Choice and Stevens’ Scales of Measurement Do Not Help Make It: A Response to Chalmers. *Educational and Psychological Measurement*, *79*(6), 1184–1197. https://doi.org/10.1177/0013164419844305
